# Interpreting Functional Impact of Genetic Variations by Network QTL for Genotype–Phenotype Association Study

**DOI:** 10.3389/fcell.2021.720321

**Published:** 2022-01-26

**Authors:** Kai Yuan, Tao Zeng, Luonan Chen

**Affiliations:** ^1^ Key Laboratory of Systems Biology, Shanghai Institute of Biochemistry and Cell Biology, Center for Excellence in Molecular Cell Science, Chinese Academy of Sciences, Shanghai, China; ^2^ Bio-Med Big Data Center, Shanghai Institute of Nutrition and Health, University of Chinese Academy of Sciences, Chinese Academy of Sciences, Shanghai, China; ^3^ Guangzhou Laboratory, Guangzhou, China; ^4^ Key Laboratory of Systems Health Science of Zhejiang Province, Hangzhou Institute for Advanced Study, University of Chinese Academy of Sciences, Chinese Academy of Sciences, Hangzhou, China; ^5^ School of Life Science and Technology, ShanghaiTech University, Shanghai, China; ^6^ Center for Excellence in Animal Evolution and Genetics, Chinese Academy of Sciences, Kunming, China

**Keywords:** genetic variation, expression quantitative trait loci, network quantitative trait loci, data integration, single-sample network, single cell, network trait, network signature

## Abstract

An enormous challenge in the post-genome era is to annotate and resolve the consequences of genetic variation on diverse phenotypes. The genome-wide association study (GWAS) is a well-known method to identify potential genetic loci for complex traits from huge genetic variations, following which it is crucial to identify expression quantitative trait loci (eQTL). However, the conventional eQTL methods usually disregard the systematical role of single-nucleotide polymorphisms (SNPs) or genes, thereby overlooking many network-associated phenotypic determinates. Such a problem motivates us to recognize the network-based quantitative trait loci (QTL), i.e., network QTL (nQTL), which is to detect the cascade association as genotype → network → phenotype rather than conventional genotype → expression → phenotype in eQTL. Specifically, we develop the nQTL framework on the theory and approach of single-sample networks, which can identify not only network traits (e.g., the gene subnetwork associated with genotype) for analyzing complex biological processes but also network signatures (e.g., the interactive gene biomarker candidates screened from network traits) for characterizing targeted phenotype and corresponding subtypes. Our results show that the nQTL framework can efficiently capture associations between SNPs and network traits (i.e., edge traits) in various simulated data scenarios, compared with traditional eQTL methods. Furthermore, we have carried out nQTL analysis on diverse biological and biomedical datasets. Our analysis is effective in detecting network traits for various biological problems and can discover many network signatures for discriminating phenotypes, which can help interpret the influence of nQTL on disease subtyping, disease prognosis, drug response, and pathogen factor association. Particularly, in contrast to the conventional approaches, the nQTL framework could also identify many network traits from human bulk expression data, validated by matched single-cell RNA-seq data in an independent or unsupervised manner. All these results strongly support that nQTL and its detection framework can simultaneously explore the global genotype–network–phenotype associations and the underlying network traits or network signatures with functional impact and importance.

## Introduction

An enormous challenge in the post-genome era is to annotate and resolve the consequences of diverse genetic variations (Lynch and Hsiao, 2019; Strober et al., 2019; Young et al., 2019), particularly within the context of human diseases (Gibbs et al., 2010; Liang et al., 2013; Zeggini et al., 2019). The genome-wide association study (GWAS) is a well-known method to identify potential genetic loci for complex diseases from a large number of genetic variations (Gamazon et al., 2010); however, the target genes and underlying mechanisms remain massively unknown due to absent functional information for the vast majority of loci (Zhu et al., 2016; Mason et al., 2018). As a crucial mechanism of genetic variants that affect gene expression (Kang et al., 2012; Peters et al., 2016; Strunz et al., 2018), expression quantitative trait loci (eQTL) indicate genomic loci that contribute to variations in gene expression levels, which reveals the connection between single-nucleotide polymorphisms (SNPs) and genes on functions rather than on sequences (Li et al., 2016), supplying detailed functional explanations of GWAS outcomes (Michaelson et al., 2009; Holloway et al., 2011; Peterson et al., 2016; Joehanes et al., 2017; Son et al., 2017; Guo et al., 2018).

To date, the eQTL method can be carried out across different genetic populations (Chen et al., 2020) in a cell-type-specific manner (Westra et al., 2015; Kasela et al., 2017; Vuckovic et al., 2020), which can generate specific molecular hypotheses (Wu et al., 2008; Thibodeau et al., 2015), e.g., the lineage-specific regulators (Liu et al., 2015; O'Brien et al., 2018) or additional pathway members (Kabakchiev and Silverberg, 2013; Zhang et al., 2018). However, the efficiency issues of eQTL methods are still widely focused in many methodology studies, such as how to illuminate the full structure of the eQTL data (Huang et al., 2009); how to distinguish true causal polymorphisms or causal factors (Suthram et al., 2008; Lee et al., 2009; Chipman and Singh, 2011; Wang and Zhang, 2011); how to implement multiple-comparison adjustment (Chen et al., 2008) or confounding factor removal (Ju et al., 2017; Yuan et al., 2017); how to detect group-wise and individual associations between SNPs and expression traits (Cheng et al., 2015; 2016); and how to calculate fast for the computationally intensive part of the eQTL identification algorithm (Shabalin, 2012). Particularly, an urgent task nowadays is to capture the functional impact of detected eQTLs. Although many of the conventional eQTL methods tend to use the network concept or model to interpret the biological or biomedical significance of their discovery (Sun et al., 2007; Verbeke et al., 2013; Cheng et al., 2014; Ho et al., 2014; Zhang and Kim, 2014; De Maeyer et al., 2016), most of them derive the associations between SNP and gene groups rather than between SNP and gene-pair/edge groups (networks); i.e., they usually disregard the systematical role of those discovered SNPs or genes, thereby overlooking many network-associated phenotypic determinates.

Such a problem motivates us to investigate network QTL (nQTL), which indicates the cascade association of genotype → network → phenotype rather than traditional genotype → expression → phenotype in eQTL. Of note, conventional SNP–gene–gene triplets (Fraser et al., 2010; Budach et al., 2016) would directly contain two SNP–gene pairs as eQTLs. By contrast, nQTL consists of associated genotype on the SNP variant and network on gene co-expression. Several recent works have combined the eQTL and gene co-expression (module) (Kugler et al., 2013; Saha et al., 2017; Kolberg et al., 2020; Cui et al., 2021). Dissimilar to them, the SNP–gene involved in nQTL is not strictly limited to eQTL; thus, the identification of nQTL would be more general to model the genotype–phenotype association on the gene network level, which might be disregarded on the gene level.

**FIGURE 1:**
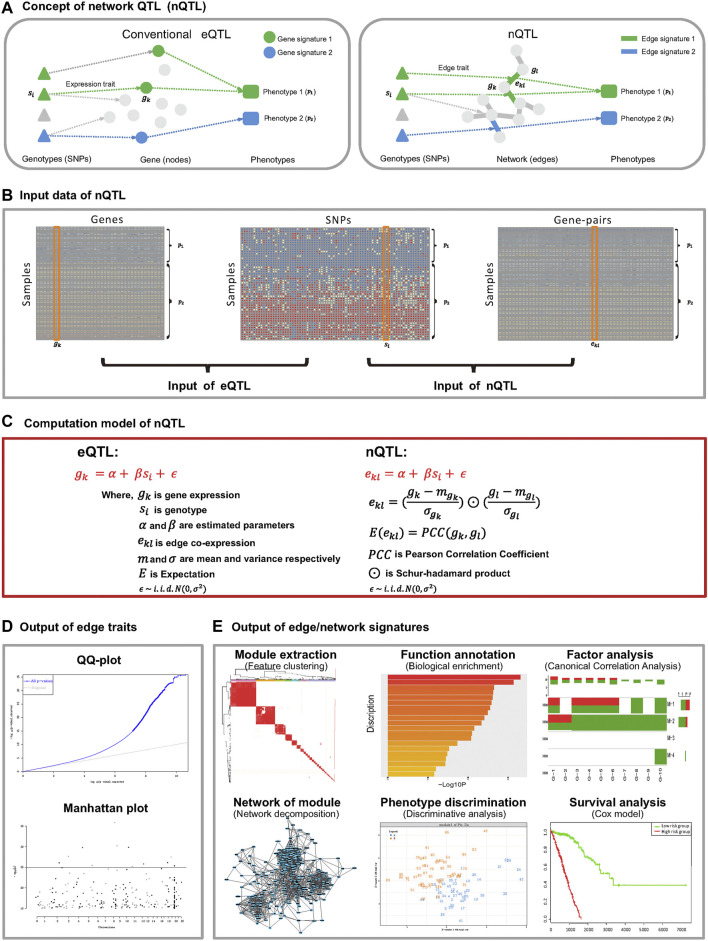
Flow chart of the nQTL framework. It includes upstream nQTL identification and downstream biological and biomedical significance analyses of nQTL gene, network, and module. **(A)** Concept of nQTL compared to conventional eQTL. **(B)** Organization of input data for eQTL and nQTL analyses. **(C)** Computational model for eQTL and nQTL analyses. **(D)** The output of nQTL analysis as a network trait, which is different from the conventional expression/gene trait of eQTL analysis. **(E)** Downstream output of network signatures in the nQTL analysis framework, including nQTL module identification, biological enrichment of module, correlation between module and relevant factors, network structure of module, discriminative model, and Cox model based on the nQTL module.

In this work, we propose a new nQTL analysis framework to study the associations from genotypes to networks and further to phenotypes at a system level (Figure 1A). The first stage is to convert the transcriptome data of individual samples from genes’ expression data into gene-pairs’ correlation-like data based on the single-sample network (SSN) theory and method (Zhang et al., 2014; Hu et al., 2018) (see Figure 1B and step 1 of the nQTL framework in Methods); then the second stage is to apply the MatrixEQTL (Shabalin, 2012) approach to capture the edge/network traits, i.e., associations between SNPs and edges/gene-pairs across samples (see Figure 1C and steps (ii) and (iii) of the nQTL framework in Methods), in contrast with expression/gene traits identified by conventional eQTL methods; the third stage is to extract the edge/network signatures (i.e., the network signature generally includes a set of edge signatures where one edge signature is an edge/gene-pair corresponding to network traits), anchoring with hot SNPs (see Figure 1D and steps (iv) and (v) of the nQTL framework in Methods); in particular, an edge trait (a gene-pair trait) of nQTL in a specific sample includes additional association information from other samples (same type or phenotype), different from traditional co-expression gene-pair (with the information of only this specific sample) due to our edge-like correlation scheme. And the last stage is to infer the links/associations between network signatures and targeted (interested) phenotypes/factors, which construct the complete cascade associations of genotype → network → phenotype (see Figure 1E and steps (vi) and (vii) of the nQTL framework in Methods). The simulation studies demonstrate the efficiency of the nQTL framework to capture network traits, compared with traditional eQTL methods. And several case studies by the nQTL framework have been carried out for various real datasets. The first case study on lung cancer data detected lung-cancer-associated network signatures, whose efficiency was validated in an independent survival analysis. The second case study on Cancer Cell Line Encyclopedia (CCLE) tumor cell line data supports the efficiency of the nQTL framework again by providing additional evidence of network signature interpretation on both disease and drug treatment phenotypes. Importantly, the third case study on both healthy human bulk and single-cell RNA-seq data identified immune-associated network traits, network signatures, and *a posteriori* targeted phenotypes (e.g., inflammatory factors) with the corresponding genotypes at the single cell level. Collectively, these results show that the nQTL framework as a novel computational tool can detect the global genotype–network–phenotype associations, which reveals the underlying network traits and network signatures, indicating the significantly functional impacts of nQTL on biology and biomedicine.

## Materials and Methods

### Edges in nQTL

For nQTL analysis, the input data in concept consists of two data matrices. One is the sequence variant data matrix (e.g., SNP data of each sample), and the other one is the network data matrix (e.g., co-expression data of individual samples), where a row represents a gene-pair/edge, a column represents the matched sample as same as that in the sequence variant data matrix, and every element represents the co-expression level of one gene-pair/edge in one sample. Notably, the sample-specific co-expression is a key to achieve nQTL analysis. Recent studies have supplied a few methods to estimate such network measurements in one sample. Considering the generality of nQTL analysis, the single-sample Pearson correlation coefficient (PCC) is adopted in this work by edge-like transformation (Zeng et al., 2014) and can be viewed as a correlation-like expression at an individual sample level because the summation of such information for all single-samples is actually the PCC in terms of mathematical representation. This kind of measurement is originally developed to explain the heterogeneity of cancer samples (Zhang et al., 2014; Yu et al., 2015; Zhang et al., 2015) and has also been applied in some other fields recently (Hu et al., 2018; Lu et al., 2019). Given the original gene expression matrix, the *PCC* between genes *i* and *j* in population samples can be calculated as follows:

$$PCC\left(i,j\right)=\frac{1}{n}\overset{n}{\underset{k=1}{\sum }}\left(\frac{x_{ik}-\mu_{i}}{\sigma_{i}}\cdot \frac{x_{jk}-\mu_{j}}{\sigma_{j}}\right)=\frac{1}{n}\overset{n}{\underset{k=1}{\sum }}e_{ijk}$$

where there are *n* samples; for sample *k*, its expressions on genes *i* and *j* are *x*_*ik*_ and *x*_*jk*_, respectively; the average expression and variance for gene *i* (or gene *j*) on population samples are *μ*_*i*_ and *σ*_*i*_ (or *μ*_*j*_ and *σ*_*j*_), respectively. The strength of each edge is obtained by transforming the expressions of two genes (i.e., *x*_*ik*_ and *x*_*jk*_) to the edge-like correlation of the gene-pair (i.e., *e*_*ijk*_) in sample *k*. Clearly, as indicated by the above equation, the mean of the edge-like correlation of a gene-pair in all samples is just the PCC (i.e., *PCC*(*i*, *j*)) on all *n* samples, so that this measurement has an equivalent numerical meaning for any sample. Repeating this data transformation for candidate gene-pairs (e.g., all matched gene-pairs in numeric or prior selected gene-pairs in a biological context), we obtain the network data matrix. Finally, MatrixEQTL can be applied to infer the association between SNPs and gene-pairs (i.e., network trait) directly and efficiently. And as mentioned above, many other alternative sample-specific network construction methods could be applied in the nQTL framework (Liu et al., 2016; Yu et al., 2017; Dai et al., 2019; Kuijjer et al., 2019; Tanaka et al., 2020), although they would hold by particular mathematical/biological hypothesis different from conventional QTL analysis. For example, the SSN approach would associate genotype with sample-specific network changes due to its different quantifications on Pearson correlation change of gene-pairs (Liu et al., 2016; Yu et al., 2017), and the cell-specific network (CSN) approach could link genotype to nonlinear correlation of gene-pairs on the basis of mutual information (Dai et al., 2019).

### nQTL Framework

Based on our nQTL concept and model, the whole nQTL framework is designed and implemented to carry out the association cascade analysis among genotype → network → phenotype. It includes several calculation steps (where the front three steps are identifying nQTL and the remaining steps are evaluating the biological or biomedical significance corresponding to genes, networks, and modules involved in nQTL):

(i) Preparing the datasets for nQTL analysis, e.g., genotype dataset, molecular phenotype dataset, physiological phenotype dataset, and some optional datasets (e.g., covariates). The network (phenotype) dataset, especially, can be produced from the molecular phenotype dataset (Figure 1), and it is a new data matrix whose row represents the gene-pair, column represents the sample, and matrix element indicates the single-sample co-expression level of one gene-pair on one sample. As required in such a study, all types of data should have values on the same samples; e.g., the samples and their ID labels should be matched in all datasets;
(ii) Carrying out the nQTL analysis for edge traits as introduced above, where an edge trait is the significant association between a SNP and an edge/gene-pair across samples and an expression/gene trait is the significant association between a SNP and a gene across samples;
(iii) Filtering the nQTL or eQTL associations (i.e., edge traits or expression traits) according to hot SNPs and hot gene-pairs/edges (or hot genes); e.g., the SNPs with significantly more interactive gene-pairs relative to other SNPs or the gene-pairs with significantly more interactive SNPs compared with other gene-pairs are kept in the following analysis (thus, their association matrix would be more dense than the original one);
(iv) Obtaining the network signatures (e.g., gene/gene-pair modules) based on their association profiles with SNPs, e.g., the so-called nQTL module from hierarchical clustering on the nQTL association matrix, where a network signature is a cluster of edges corresponding to their edge traits (an edge signature is an edge corresponding to its edge trait) and a gene signature is a gene corresponding to its expression/gene trait;
(v) Estimating the functional enrichment of each network signature, e.g., GO or KEGG enrichment;
(vi) Inferring the link/association between network signatures (e.g., gene-pair co-expression measurements) and physiological phenotypes (e.g., clinical indices or factors) by partial least squares discriminant analysis (PLSDA) or canonical correlation analysis (CCA); for instance, in the following formula, when we expect to infer the association between a cluster of gene-pairs and a set of clinical indices (e.g., several markers), the co-expression data matrix of gene-pairs (e.g., *X*) and the phenotypic data matrix of indices (e.g., *Y*) on a group of same samples can be applied to calculate the canonical correlation (e.g., *cca*(*X*, *Y*)).

$$X^{\prime }=a^{T}X$$

$$Y^{\prime }=b^{T}Y$$

$$\text{cca}\left(X,Y\right)=\underset{a,b}{\max}\frac{cov\left(X^{\prime },Y^{\prime }\right)}{\sqrt{D\left(X^{\prime }\right)}\sqrt{D\left(Y^{\prime }\right)}}$$

(vii) Ranking and selecting the association cascades on genotype → network → phenotype based on significance of network trait, functional enrichment, and phenotypic relevance of network signatures together.

Besides, the “guilt-by-association” analysis can be carried out with nQTL, such as the comparison and combination with results from conventional eQTL, WGCNA, PCA, etc.

Note that an edge (gene-pair) is represented by its correlation strength (*e*_*ijk*_ for genes *i* and *j* on sample *k*), while a gene (node) is represented by its expression (*x*_*ik*_ for gene *i* on sample *k*) in this paper.

### Synthetic Data Production and Method Assessment

For conventional eQTL analysis, the required data usually consist of two data matrices. One is the sequence variant data matrix (e.g., SNP data), where each row represents a SNP, each column represents a sample, and each element represents the genotype of one SNP in one sample. The other one is the expression data matrix (e.g., gene expression data), where one row represents a gene, one column represents the matched sample as same as that in the sequence variant data matrix (i.e., matched samples), and each element represents the expression level of one gene in one sample.

The general eQTL model of the association between SNPs and genes (i.e., expression trait) can be calculated as a simple linear regression or ANOVA. On the one hand, for each gene–SNP pair, with SNP encoded by the variant frequency, the linear association between gene expression *g* and genotype *s* is modeled as

$$\text{g}=\text{α}+\text{β}\cdot \text{s}+\varepsilon \;,\;where\;\varepsilon \in \text{i}.\text{i}.\text{d}.\text{ N}\left(0,\sigma^{2}\right)$$

On the other hand, for each gene–SNP pair, with SNP encoded by the categorical dominant effect, the ANOVA model for a linear regression between gene expression *g* and genotype *s* (e.g., *s*_1_ and *s*_2_ are dummy variables) is described as

$$\text{g}=\text{α}+{\text{β}}_{1}\cdot {\text{s}}_{1}+{\text{β}}_{2}\cdot {\text{s}}_{2}+\varepsilon ,\;where\;\varepsilon \in \text{i}.\text{i}.\text{d}.\text{ N}\left(0,\sigma^{2}\right)$$

To effectively solve these models and handle covariate issues, the MatrixEQTL has been implemented to analyze different types of eQTL problems (Shabalin, 2012). Of note, in the above two models, the ε is measured for noise.

We generated eight sets of simulated datasets for identifying eQTL and nQTL, as supplied in MatrixEQTL (Shabalin, 2012).

For the first four sets of simulated data, the relation between gene and SNP (i.e., expression trait) is implemented by following formula:

pop=0.2∗rnorm(n)

snps=rnorm(n∗nss)+pop

$$\text{ind}=\text{snps}\left[,\text{sample}\left(1:\text{nss},\text{ngs}\right)\right]\text{∗}\left(1:\text{ngs}\right)/{\text{ngs}}^{9/2}$$

gene=rnorm(n∗ngs)+pop+ind

where *n* is the number of samples, *ngs* is the number of genes, *nss* is the number of SNPs, *pop* is the common signal in all variables, and *ind* is the individual genetic signal to gene expression. We set the sample number as 100, the gene number as 2,000 and 5,000, and the SNP number as 2,000 and 200,000. Obviously, there are four kinds of data combinations from gene and SNP. Of note, in order to make comparable eQTL evaluation with MatrixEQTL, we adopted the above data simulation approach as available through MatrixEQTL.

For the other four sets of synthetic data, SNP and gene data are generated randomly, and we associate gene-pair data with SNP (i.e., edge trait) according to the following formula:

 gene=rnorm(n∗ngs);

cordata=cor+pop+ind;

where the additional variable *cor* means the value of gene-pairs. Then, we set the sample size as 100, the gene and gene-pair number as (2,000, 1,999*1,000) and (5,000, 4,999*2,500), and the SNP number as 2,000 and 200,000; again, there are four kinds of data combinations from gene/gene-pair and SNP.

### Data Collection and Organization of Lung Cancer Dataset

As reported in the original study of Data GSE28571 (Micke et al., 2011), the surgically treated patients with primary non-small-cell lung cancer (NSCLC) were selected with fresh frozen NSCLC tissues, and patients with a history of other cancers or neoadjuvant treatment were excluded. Array experiments were performed according to the standard protocols for Affymetrix GeneChip Mapping 250K Nsp I arrays. In our reanalysis on these data, we have chosen 97 samples (as listed in Supplementary Table S1) because they have tested both the matched expression and SNP data with 261,549 SNPs and 23,322 annotated genes. These samples also include short-term (<20 months; *n* = 52) and long-term survivors (>58 months; *n* = 45), which can be used as a clinical phenotype index. And the protein–protein interaction (PPI) network from the STRING database was applied to select gene-pairs for this, and following nQTL analysis on human data, where we have chosen the gene-pairs whose PPI score is >900. Thus, three preprocessed data matrices were used for conventional eQTL and new nQTL analyses. The SNP data *S* as a matrix consists of 261,549 rows and 97 columns, and each element indicates the SNP mutation type or score of one SNP in one sample. The expression data *E* consists of 23,322 rows and 97 columns, and each element indicates one gene’s expression level in one sample. The co-expression data *C* consists of 147,645 rows and 97 columns, and each element indicates one gene-pair’s co-expression level in one sample. Thus, *S* and *E* were the inputs of conventional eQTL analysis; *S* and *C* were the inputs of the new nQTL analysis.

### Data Collection and Organization of CCLE Hematopoietic Data

We collected the CCLE: a compilation of gene expression, chromosomal copy number, and massively parallel sequencing data from 947 human cancer cell lines (Barretina et al., 2012). Coupled with pharmacological profiles for 24 anticancer drugs across 479 of these cell lines, this collection allowed identification of genetic, lineage, and gene-expression-based predictors of drug sensitivity. Genotyping/copy number analysis was performed by Affymetrix Genome-Wide Human SNP Array 6.0, and expression analysis used the GeneChip Human Genome U133 Plus 2.0 Array. Eight-point dose–response curves were generated for 24 anticancer drugs by an automated compound-screening platform.

We chose the hematopoietic and lymphoid tissue as a case study of nonsolid tumor. In this CCLE hematopoietic data, we selected 21,544 gene expression data and 906,600 SNP data for 151 matched samples (as listed in Supplementary Table S1). Of note, we focused on the nQTL compared to conventional eQTL, where the expression level of mRNA/protein is proportional to the quantitative trait and the prior-known gene/protein network are abundant. Thus, the data of annotated long noncoding RNA (ncRNA) or micro-RNA in the original CCLE hematopoietic data were not used in this study. In addition to these eQTLs and nQTLs in protein-coding genes, the QTLs in ncRNAs would also be attractive in future work, which could reveal the association between genotype and regulation of diverse ncRNAs (Ye et al., 2020).

### Data Collection and Organization of the Human Peripheral Blood Mononuclear Cell (PBMC) Dataset

The original study focused on the cell-type-specific effects of genetic variation on genome-wide gene expression, which generated scRNA-seq data of ∼25,000 PBMCs from 45 donors of the population-based cohort study Lifelines Deep (van der Wijst et al., 2018). To assure sufficient analysis power, cell types were merged to a more general classification: CD4^+^ T cells, CD8^+^ T cells, NK cells (CD56^dim^ CD16^+^ and CD56^bright^ CD16^+/−^), monocytes (CD14^bright^ CD16^–^ cMonocyte and CD14^dim^ CD16^+^ ncMonocyte), B cells and DCs (CD1C^+^ myeloid, mDC, and plasmacytoid, pDC). In detail, the original study had proposed the concepts of co-expression QTLs by associating the SNP type and gene-pair co-expression based on single-cell data (van der Wijst et al., 2018), which can be thought of as a supervised way to infer the SNP and gene-pair association. It prepared the gene expression data of one individual/sample by averaging his/her single cells’ gene expression, which could be thought of as a mimic bulk expression of individuals; and it also generated the gene co-expression data of one individual/sample by calculating the gene expression correlation of two genes on single cells from this individual; and then it carried out the eQTL analysis by combining the SNP *vs.* individual matrix and mimic bulk expression *vs.* individual matrix; it also detected co-expression QTL through the eQTL model on SNP *vs.* individual matrix and co-expression *vs.* individual matrix. Similar to such a study, in this work, we carried out eQTL and nQTL analyses, based on the expression data of CD4^+^ T cells. Thus, in the 45 individuals, there are 7,478 genes in the expression matrix and 4,017,251 SNPs in the genotype matrix.

## Results

### Assessment of nQTL Framework on Synthetic Datasets for Gene and Network Traits

Similar to the simulation experiment used by a conventional eQTL study (Shabalin, 2012), we generated eight sets of simulated datasets. The parameter setting includes the following: (1) for the first four datasets with preset expression traits, the sample size is 100, the gene number is from 2,000 to 5,000, and the SNP number is from 2,000 to 200,000; (2) for the latter four datasets with preset edge/network traits, the sample size is 100, the gene and gene-pair numbers are from (2,000, 1,999*1,000) to (5,000, 4,999*2,500), and the SNP number is from 2,000 to 200,000.

**FIGURE 2:**
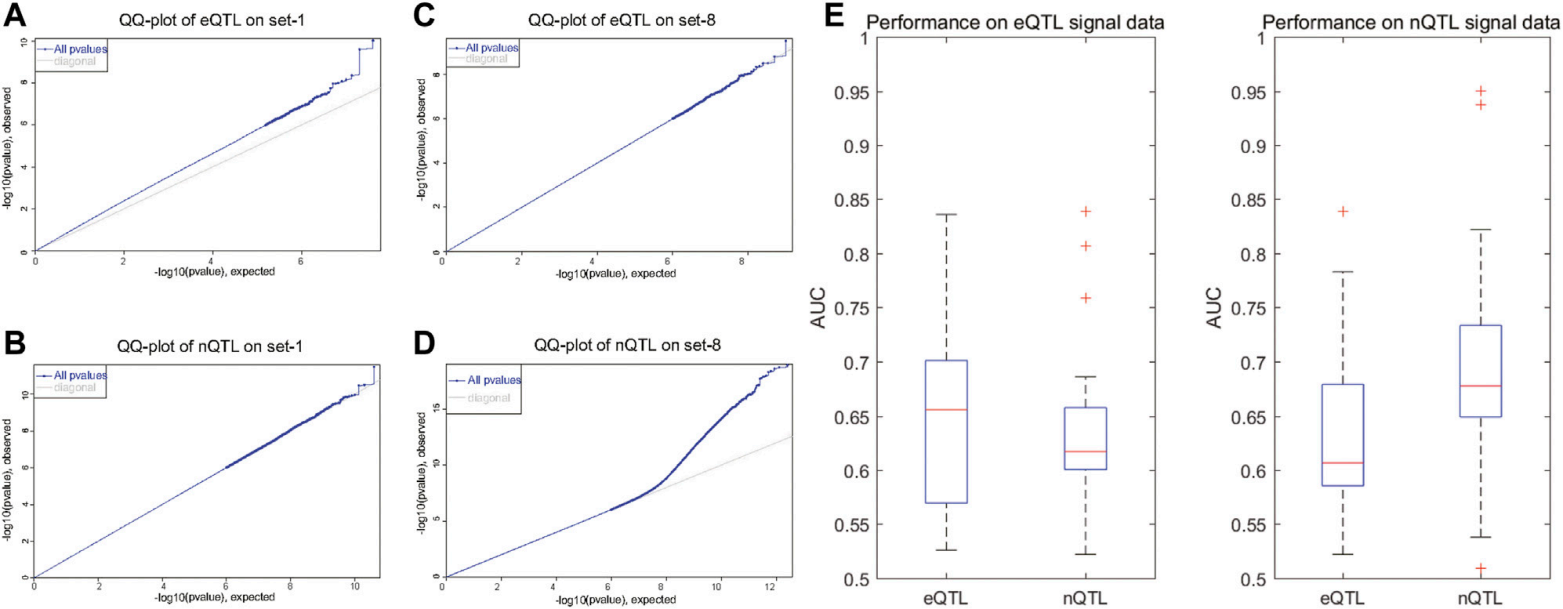
Comparison of eQTL and nQTL identification on synthetic datasets based on expression traits and network traits. **(A**,**B)** QQ-plot of eQTL and nQTL, respectively, on a simulated data with preset expression traits. **(C**,**D)** QQ-plot of eQTL and nQTL, respectively, on a simulated data with preset edge traits. **(E)** AUC measurement comparison between nQTL and eQTL identifications on multiple datasets with preset expression traits. **(F)** AUC measurement comparison between nQTL and eQTL identifications on multiple datasets with preset edge traits.

As known in GWAS, there will be a large deviation at one SNP site in the QQ-plot, suggesting that the deviation of the observed value of this SNP site is caused by the genetic effects of this SNP mutation. With the results shown in the QQ-plot, it is obvious that the eQTL analysis can find significant SNP–gene associations in the first four datasets with preset expression/node traits rather than other datasets with preset edge traits (Figure 2A and Supplementary Figure S1); the eQTL analysis can find the SNPs which have a bigger deviation; meanwhile, the nQTL analysis is comparable in spite of less deviation (Figure 2B and Supplementary Figure S1). By contrast, the nQTL analysis can detect significant SNP–(gene-pair) associations in the latter four datasets with preset edge traits (Figure 2D and Supplementary Figure S1), but the eQTL analysis only can detect a limited number of associations (Figure 2C and Supplementary Figure S1). In further quantitative comparison, the area under the curve (AUC) measurement was applied to compare the method performance, which is an accuracy measurement considering the identification specificity and sensitivity under different threshold adoptions. As shown in Figure 2E, on multiple datasets with preset expression/node traits, the nQTL analysis has slightly small but comparable AUC values to those of eQTL (i.e., nonsignificant difference observed, *p* = 0.3463); meanwhile, as shown in Figure 2F, on multiple datasets with preset edge traits, nQTL analysis indeed has larger AUC values than those of eQTL analysis (i.e., significant difference observed, *p* = 0.031). These results on synthetic datasets support the ability and efficiency of the nQTL framework to detect novel network traits, in which each edge trait means an association between a genotype and a gene-pair co-expression (or an edge in network) and an expression trait (or a gene trait) means an association between a genotype and a gene expression (or a node in network).

### nQTL Framework Identified Network Traits and Network Signatures Effective for Phenotype Discrimination and Disease Prognosis on Lung Cancer

We carried out the nQTL analysis on the public data GSE28571 (Micke et al., 2011) to detect new genotype–phenotype associations of lung cancer on a network level, with a comparison of conventional eQTL analysis.

**FIGURE 3:**
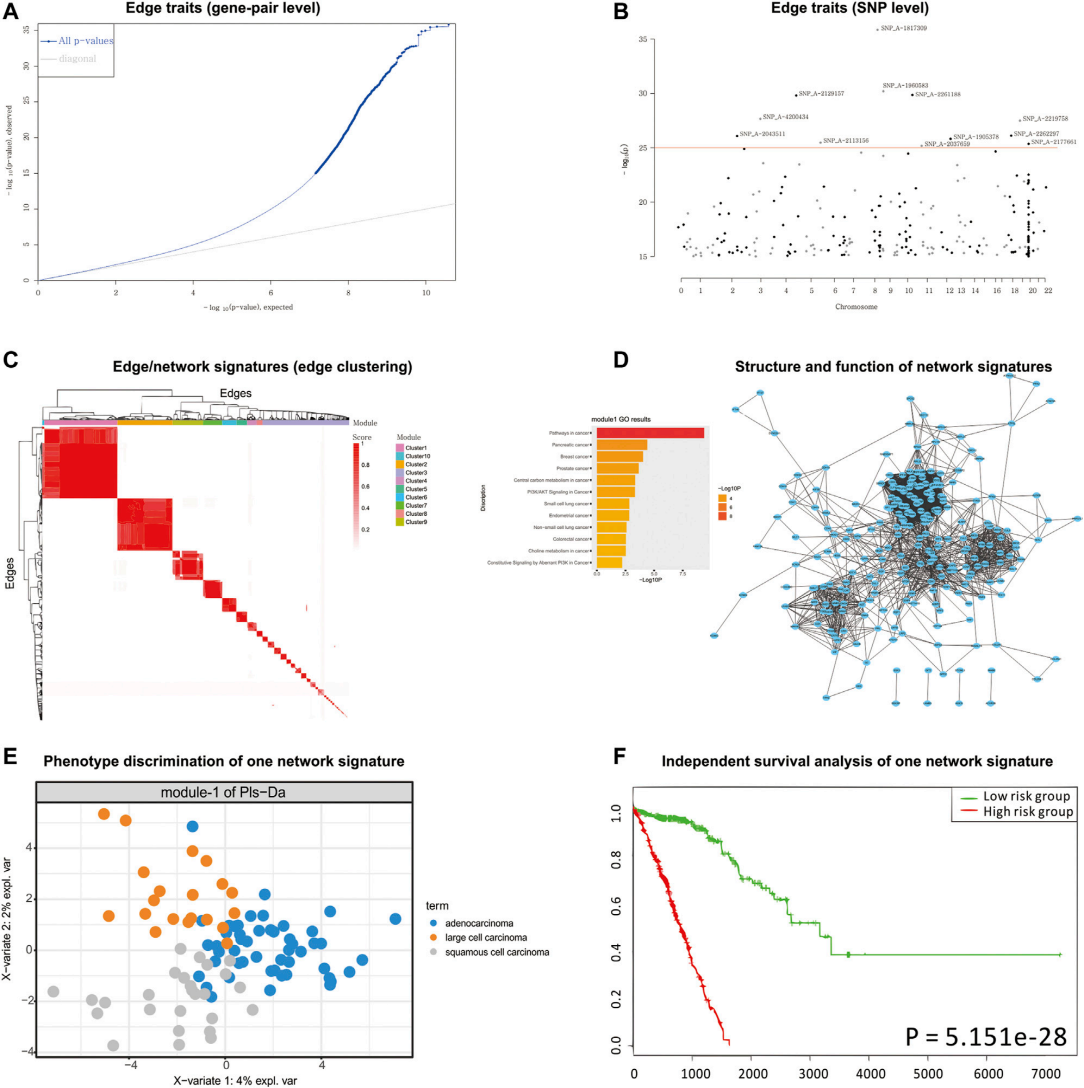
nQTL analysis results from lung cancer dataset. **(A)** QQ-plot of edge trait on the gene level. **(B)** Manhattan plot of edge trait on the SNP level. **(C)** Network signature from edge/gene-pair clustering. **(D)** The network structure and function of one network signature. **(E)** PLSDA of one significant network signature for subtyping a disease. **(F)** Survival analysis and validation of one significant network signature.

According to the detected eQTLs (Supplementary Table S2) and nQTLs (Supplementary Table S3) with their QQ-plot (Figure 3A and Supplementary Figure S2), we found that the nQTL framework obviously tends to have more significant discoveries on edge traits. Several extremely significant SNPs, especially, can be filtered and shown in the Manhattan plot (Figure 3B and Supplementary Figure S3) by nQTL rather than eQTL analysis. Although these two methods can significantly detect similar SNPs and genes (Supplementary Figure S4), nQTL analysis can provide more alternative candidates than eQTL analysis. Of note, in this and following case studies, there is no more additional preprocessing or control (Micke et al., 2006; Nguyen et al., 2006; Botling et al., 2009; Platig et al., 2016) on the SNPs involved in nQTL or eQTL, which can reserve sufficient association information for downstream network and function analysis.

For the downstream analysis of the nQTL framework, the top-ranked 1,000 gene-pairs/edges (hot edges) with many SNP connections (i.e., each gene-pair can associate with more than 330 SNPs) are selected. Their association matrix can be extracted and clustered as in Figure 3C and Supplementary Figure S5A, and those edges can be obviously grouped into several network signatures (i.e., edge/gene-pair clusters in Supplementary Figure S5B). Many network properties of PPI subnetworks corresponding to such network signatures (e.g., Supplementary Figures S6 and S7) indicate that nQTL identification can effectively detect the SNPs associated with gene networks; by contrast, the gene cluster based on eQTL analysis would produce lots of small gene groups or even individual genes, which cannot reflect the biological network underlying QTL (Supplementary Figure S7). Here, a network signature is a gene-pair/edge cluster in terms of not their expressions but their correlations, whereas a gene signature is a gene in terms of its expression.

Then, according to the enriched biological functions of network signatures by enrichment analysis (Supplementary Figure S8), we found that nine network signatures have many enriched pathways or functions related to lung cancer, and especially one signature with our nQTL Module 1 (Figure 3D) is significantly associated with lung cancer. We also can find pathways in cancer (Takayama et al., 2006), small-cell lung cancer (Sanchez-Cespedes, 2003), and NSCLC (Breuer et al., 2005), which support that network signatures accurately detect the phenotype-associated common functions across samples. Furthermore, some detailed signaling-related functions are also found, such as the phosphatidylinositol 3′-kinase (PI3K)-Akt signaling pathway (Hers et al., 2011), which is a key pathway associated with distinct metabolic phenotype characterized by low oxidative phosphorylation in small-cell lung cancer (SCLC) (Tripathi et al., 2017) or tumor angiogenesis in NSCLC (Zhou et al., 2017); Ras signaling pathway, which has been reported to be related with the proliferation, migration, and invasion of NSCLC cells (Levallet et al., 2012; Abdel-Rahman, 2016; Zheng et al., 2017); and MAPK signaling pathway whose activation would regulate lung cancer growth (Tang et al., 2016) and promote cell invasion and metastasis in NSCLC (Levallet et al., 2012). Besides, we also observed two well-known functions like the p53 signaling pathway and cell cycle pathway. The most recent studies have revealed that the suppression of the p53 signaling pathway could accelerate tumor progression in lung cancer (Hao et al., 2018) and that the cell cycle pathway is involved in leptomeningeal metastasis of NSCLC (Fan et al., 2018).

Finally, based on the subtypes of lung cancer available in the original study (e.g., large cell carcinoma, adenocarcinoma, and squamous cell carcinoma) (Micke et al., 2011), PLSDA can be executed and visualized on each network signature (Figure 3E and Supplementary Figure S9), and most network signatures showed significant phenotype discriminations on different subtypes. Besides, based on the binary survival data available in the original study (e.g., two groups of patients with long survival time or short survival time) (Micke et al., 2011), network signatures display satisfactory discrimination power again by PLSDA (Figure 3E, Supplementary Figures S9 and S10, and Supplementary Table S4). In addition, we also carried out independent survival analysis by nQTL network signatures’ genes on independent lung cancer datasets from SurvExpress (Aguirre-Gamboa et al., 2013) (Figure 3F and Supplementary Figure S10), where all network signatures have *p*-values less than 0.05. Of note, compared to traditional methods using gene expression alone (e.g., WGCNA co-expression modules), nQTL analysis would be more efficient (Supplementary Table S5), where the nQTL framework has found six modules with a *p*-value <1E−10 and three modules with a *p*-value <1E−20; meanwhile, WGCNA has found five modules with a *p*-value <1E−10 and two modules with a *p*-value <1E−20. Thus, nQTL analysis can build functional association cascade as genotype → network (module) → phenotype. Furthermore, the nQTL framework also displays competitive and robust performance on distinguishing different cancer subtypes (Supplementary Figures S11 and S13), because all WGCNA modules had worse effects on large cell carcinoma identification (Supplementary Figures S12 and S14), indicating potential discovery bias of conventional methods. For example, the fifth WGCNA module can only separate samples from adenocarcinoma and squamous cell carcinoma but can mix samples from large cell carcinoma (Supplementary Figure S12); meanwhile, the AUC of the corresponding PLSDA (e.g., large cell carcinoma *vs.* others) is lower at 0.6284 (Supplementary Figure S14). Besides, on another large independent survival analysis on TCGA data (Gao et al., 2013), we have found one nQTL module with a *p*-value of 2.03E−03, which is below the Bonferroni significance threshold when doing 10 tests; by contrast, no WGCNA modules show such significance (Supplementary Table S5). Of course, there are indeed many ways to factorize or cluster expression data, and the additional benchmark comparison in the future would recover more characteristics of gene clusters or gene subnetworks associated with genotype, e.g., nQTL modules.

### nQTL Framework Identified Network Traits and Network Signatures Effective for Drug Response Phenotype Discrimination and Interpretation on Cancer Cell Line

In this second case, we carried out nQTL analysis on the public CCLE dataset (Barretina et al., 2012) to detect new genotype–phenotype associations in different cancer cell lines on a network level, and we chose the hematopoietic and lymphoid tissues as an example of nonsolid tumor to illustrate the results.

**FIGURE 4:**
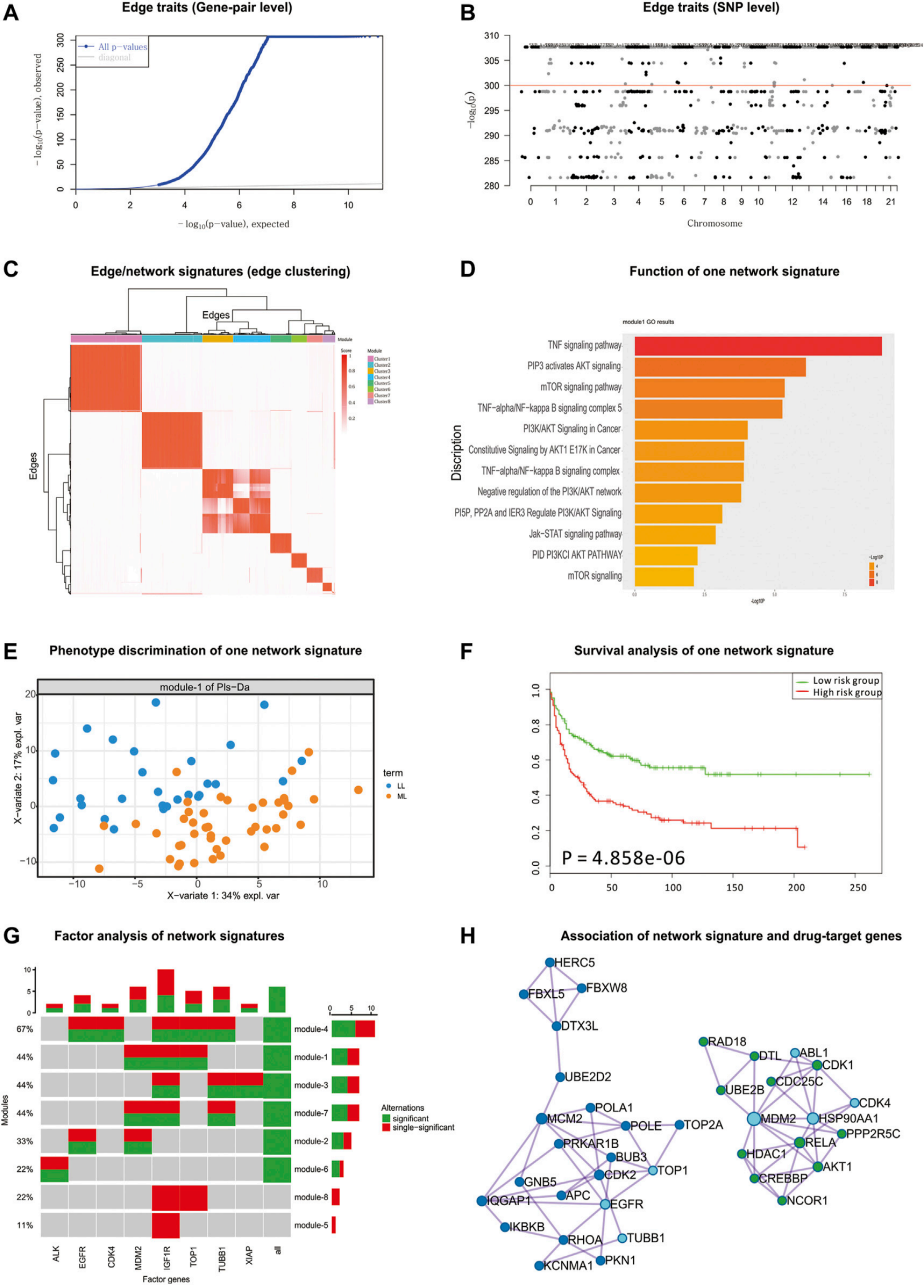
nQTL analysis results from CCLE hematopoietic data. **(A)** QQ-plot of edge trait on the gene level. **(B)** Manhattan plot of edge trait on the SNP level. **(C)** Edge/network signature from edge/gene-pair clustering. **(D)** The biological function of one significant network signature. **(E)** PLSDA of one significant network signature for two groups of patients with myelogenous leukemia and lymphocytic leukemia (i.e., M *vs.* L). **(F)** Survival analysis and validation of one significant network signature. **(G)** CCA of network signatures and drug-target genes. **(H)** Association among network signature genes and drug-target genes on the STRING network, where nodes in dark blue and green are two groups of signature genes and nodes in light blue are drug-target genes.

According to the detected eQTLs (Supplementary Table S6) and nQTLs (Supplementary Table S7) with their QQ-plot (Figure 4A and Supplementary Figure S15), we found that nQTL analysis obviously tends to have more significant discoveries on edge traits and concentrate on less genes than eQTL analysis, although these methods have significant overlap on the detected SNPs and genes (Figure 4B and Supplementary Figures S16 and S17).

By the downstream analysis of the nQTL framework on the hematopoietic and lymphoid data, the top-ranked 1,000 edges (hot edges) with many SNP connections (more than 2,000) are selected. Their association matrix can be extracted and clustered as in Figure 4C and Supplementary Figure S18A, and those edges can be obviously grouped into several network signatures (Supplementary Figure S18B). Again, many network properties of PPI subnetworks corresponding to these network signatures (e.g., Supplementary Figure S19) indicate that nQTL identification can effectively detect the SNPs associated with interacting genes; meanwhile, the gene cluster based on eQTL analysis would only detect separate or incomplete structures of biological networks underlying QTL (Supplementary Figure S19).

Next, considering the enriched biological functions of network signatures (Supplementary Figure S20), we found they have similar enriched pathways or functions relevant to lymphoid cancer and leukemia, and especially the signature Module-1 (Figure 4D) is significantly associated with lymphoid cancer. We also found pathways in cancer, lymphocyte differentiation, and lymphocyte migration, which support that network signatures are able to detect the phenotype-associated shared functions across samples efficiently. Some detailed signaling-related functions were also found. The PI3K-Akt signaling pathway is activated by many types of cellular stimuli or toxic insults and regulates fundamental cellular functions such as transcription, translation, proliferation, growth, and survival (Engelman et al., 2006). The mammalian (mechanistic) target of rapamycin (mTOR) is a highly conserved serine/threonine protein kinase, and NPM-ALK induces GC resistance in lymphoid cells through the activation of the mTOR signaling pathway (Gu et al., 2008). The tumor necrosis factor (TNF), as a critical cytokine, can induce a wide range of intracellular signaling pathways including apoptosis and cell survival as well as inflammation and immunity (MacEwan, 2002).

Then, based on the binary category of cell lines derived from disease samples available in the original study (e.g., two groups of patients with acute leukemia and chronic leukemia (i.e., A *vs.* C) or two groups of patients with myelogenous leukemia and lymphocytic leukemia (i.e., M *vs.* L)), PLSDA can be executed, evaluated, and visualized for each network signatures’ association with phenotypes (Figure 4E and Supplementary Figure S21). Many network signatures (i.e., correlations between genes) actually showed significant phenotype discriminations on two groups of samples (Supplementary Figure S8). Furthermore, we carried out independent survival analysis for these network signatures’ genes by using independent data of chronic lymphocytic leukemia (Ramsay et al., 2013) (Figure 4F and Supplementary Figure S22), where seven of eight network signatures have *p*-values less than 0.05 and half of eight modules are still significant after multiple testing corrections, which indicates the efficiency of the nQTL framework and its network signature discovery for disease subtyping and prognosis potential.

Considering mainly two different hematopoietic and lymphoid cell lines, we have summarized the differentially expressed genes between different types of cell lines. We found that on average only 7% of genes in the nQTL module were differentially expressed. Thus, cell type would be a partial driver of the conventional co-expression module; however, the nQTL approach could discover many new modules relevant to genotype independent of cell type.

Finally, according to the drug and drug-target information from the CCLE database, we collected a drug-target gene list: IGF1R, TUBB1, MDM2, TOP1, EGFR, XIAP, CKD4, ALK, etc. By CCA, we detected the significant expression associations among our network signature genes and these drug-target genes. Interestingly, there are few associations between sole gene and network signatures; by contrast, the network signatures tend to associate with a combination of drug-target genes (Figure 4G). This is also supported by the STRING subnetwork among network signature genes and drug-target genes (Figure 4H), where TUBB1, TOP1, and EGFR can interact with a group of network signature genes enriched in the functional pathway “Pathways in cancer” and MDM2 and CKD4 can cooperate with another group of network-neighbor genes enriched in the functional pathway “Chronic myeloid leukemia”.

### nQTL Framework Identified Network Traits and Network Signatures Effective for Healthy Immunity Phenotype Discrimination on Both Human Bulk and Single-Cell Transcriptomes

In this third case, we conducted nQTL analysis on scRNA-seq-induced bulk expression data corresponding to CD4^+^ T cells (van der Wijst et al., 2018), and we especially validated our discovered edge/network traits on the original scRNA-seq data in a unsupervised manner, which should be more efficient than the original method “co-expression QTL” (van der Wijst et al., 2018) when single-cell data are not available. Here, the supervision is different from that in the conventional classification model. Although co-expression QTL analysis did not require *a priori* selection of specific genes to test, it did require *a priori* selection of specific edges by a full scan of significant modifiers of edges. In other words, the co-expressions of edges are directly calculated from additional single-cell data, which can be considered as certain supervised information from external sources. By contrast, nQTL analysis is able to infer a similar association between SNPs and the correlated edges on the basis of bulk expression data, which would be validated by single-cell data independently. Thus, as summarized and shown in Supplementary Figure S39, we consider that nQTL analysis can make association analysis in an unsupervised manner, different from co-expression QTL analysis.

Of note, different from the disease study with prior-targeted phenotype, the healthy human data might have many potential phenotypes of interest. And we could focus on the nQTL analysis relevant to a post-targeted phenotype (i.e., immune factors or inflammatory factors), although some significant results might be relevant to other phenotypes.

**FIGURE 5:**
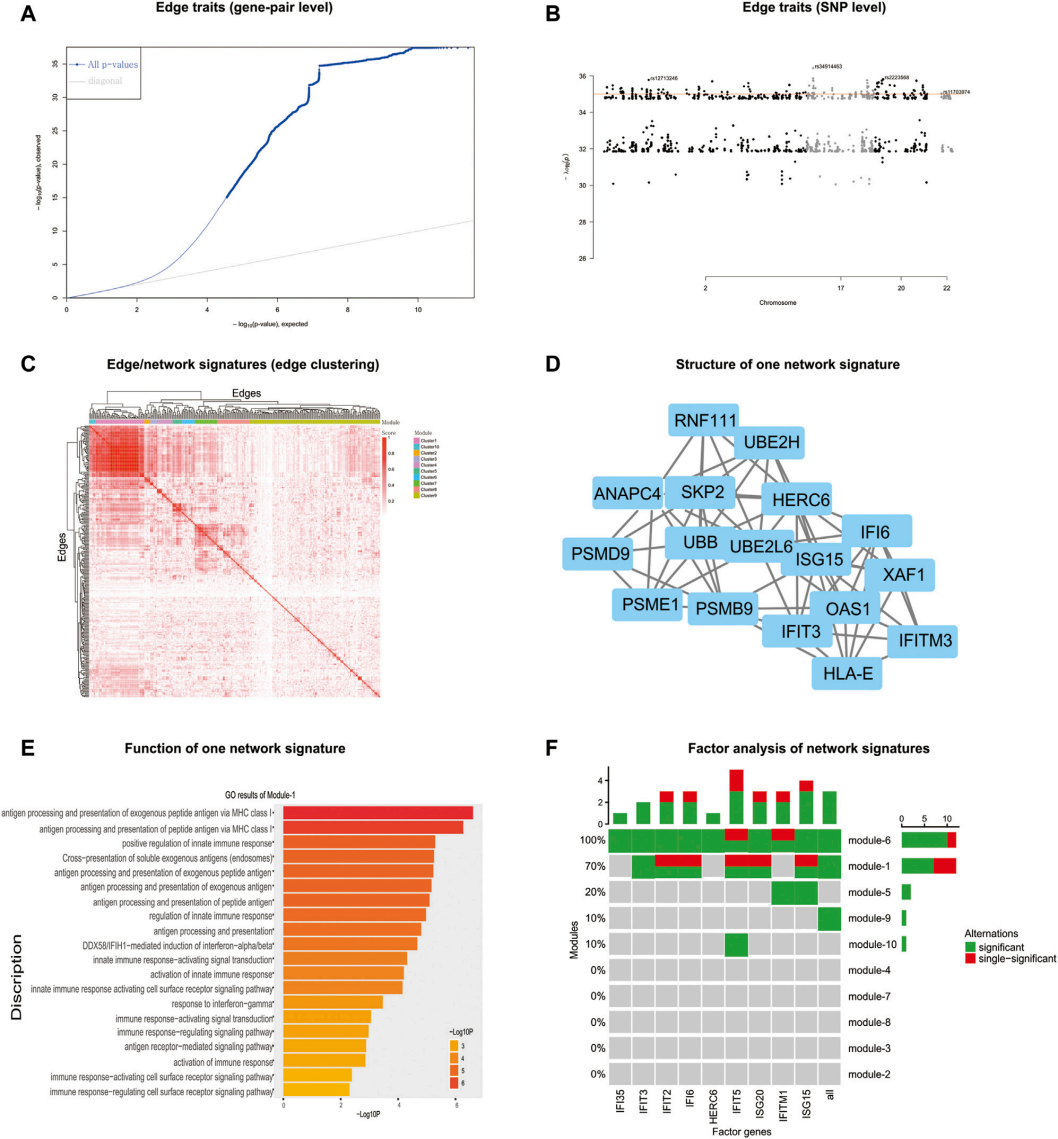
nQTL analysis results from the human PBMC dataset. **(A)** QQ-plot of edge trait on the gene level. **(B)** Manhattan plot of edge trait on the SNP level. **(C)** Network signature from edge/gene-pair clustering. **(D)** The network structure of one network signature. **(E)** GO analysis of one significant network signature. **(F)** CCA of network signatures and immune factors or inflammatory factors.

Similarly, the obtained eQTLs (Supplementary Table S9), nQTLs (Supplementary Table S10), and their QQ-plots (Figure 5A and Supplementary Figure S23) suggest that nQTL analysis is able to identify more significant discoveries on edge traits and that nQTL analysis indeed can detect more phenotype-associated SNPs than eQTL analysis according to the extremely significant SNPs filtered (Figure 5B and Supplementary Figure S24). Meanwhile, nQTL analysis can provide more alternative candidates than eQTL analysis although these two methods can find significantly overlapping SNPs and genes (Supplementary Figure S25).

Next, by selecting the top-ranked gene-pairs (hot edges) with significantly more associations to all SNPs than other edges, the association matrix can be extracted and clustered (Figure 5C and Supplementary Figure S26A), based on which those edges/gene-pairs can be grouped into a few network signatures (Supplementary Figure S26B). The network properties of PPI subnetworks induced from nQTL modules corresponding to these network signatures reveal larger network centralities than those induced from eQTL analysis (Supplementary Figures S27 and S28). Thus, the nQTL framework can effectively detect the SNP-associated gene networks, and the network signatures (Figure 5D) would be more general on annotating biological functions rather than simply considering network structure. In accordance with the biological background of the data, we observed several pathways relevant to immunity, viruses, and antigens in the biological context of PBMC. Enriched in antigen processing and presentation pathway, the genes encoding MHC class II molecules are transcribed according to a strict cell-type-specific and quantitatively modulated pattern, which is pivotal for the adaptive immune system by guiding the development and activation of CD4^+^ T-helper cells MHC class II molecules (Reith et al., 2005). And enriched in the autoimmune thyroid disease pathway, both autoantibodies and thyroid-specific cytotoxic T lymphocytes (CTLs) have been proposed to be responsible for autoimmune thyrocyte depletion (Stassi and De Maria, 2002), and self-reactive CD4^+^ T lymphocytes (Th) recruit B cells and CD8^+^ T cells (CTL) into the thyroid (Tsatsoulis, 2006).

**FIGURE 6:**
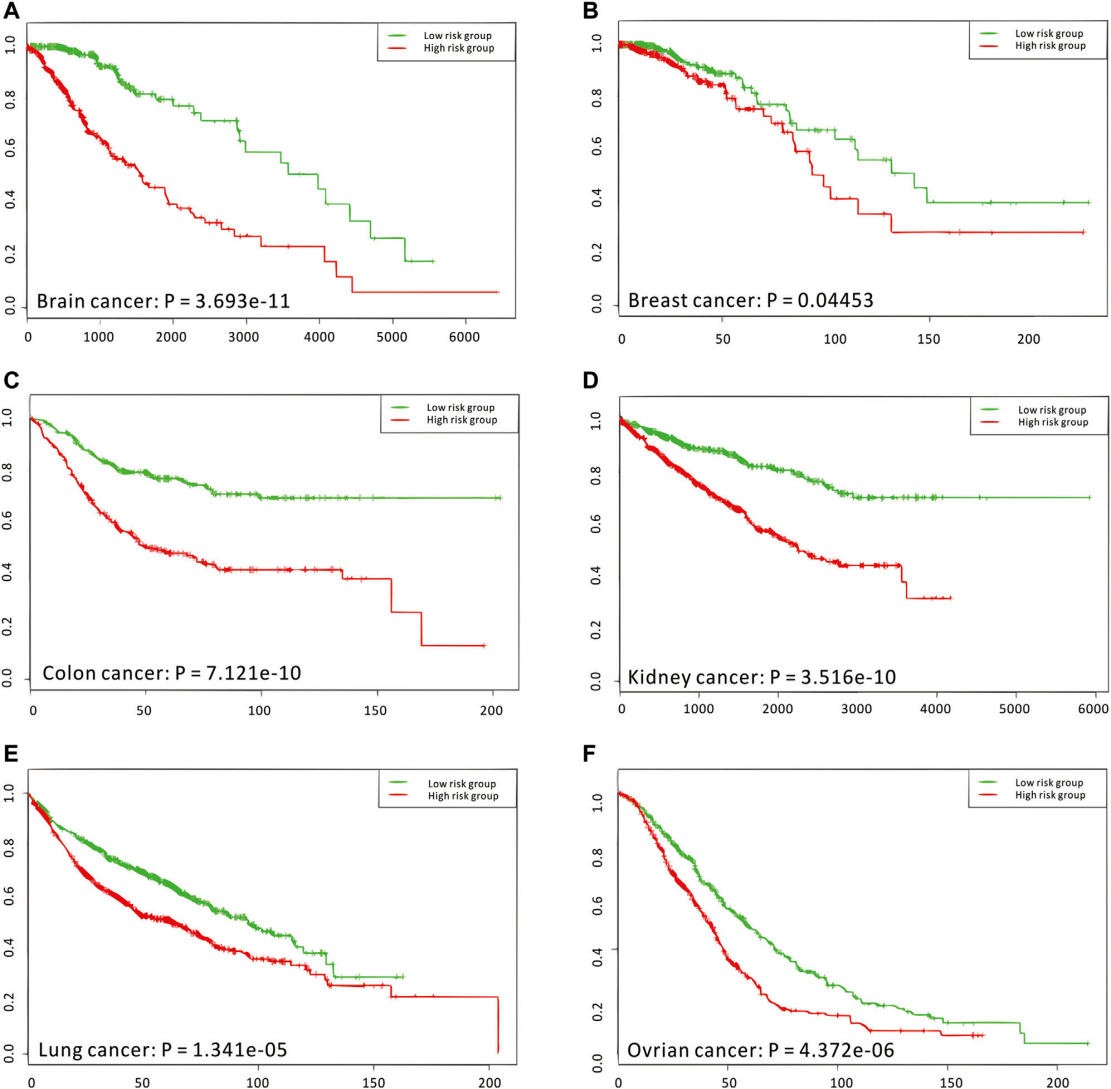
Independent survival analyses of nQTL modules from the human PBMC dataset. **(A–F)** The validation on brain, breast, colon, kidney, lung, and ovarian cancer, respectively. And these analysis results are obtained from the SurvExpress web server, where *p*-values are produced from the Cox model.

Considering that the enriched biological functions for many network signatures (Figure 5E and Supplementary Figure S29) actually show significant relations with viruses, interferons, antigens, and especially immunity, we collected and used literature-reported immune factors or inflammatory factors as a post-targeted phenotype for the following association inference. These functional factors include IFIT5, IFITM1, IFIT2, IFI6, ISG20, ISG15, IFI35, IFIT3, and HERC6. By calculating the CCA between a group of gene-pairs from one network signature and a group of functional factors (Figure 5F and Supplementary Figure S30), several network signatures showed significant relevance with many functional factors, underlying the onset of the immune system of healthy humans. On the other hand, we also carried out survival analysis by these network signature genes on independent pan-cancer datasets, assuming the immune-associated survival risks detectable in our identified network signatures. On six cancer datasets with survival data from more than 500 samples (Figure 6, Supplementary Figures S31–S36, and Supplementary Table S11), all network signatures have *p*-values less than 0.05; and even after multiple testing corrections, there are still about 83% network signatures showing significant survival analysis (Supplementary Table S11), again indicating the efficiency of nQTL discovery.

**FIGURE 7:**
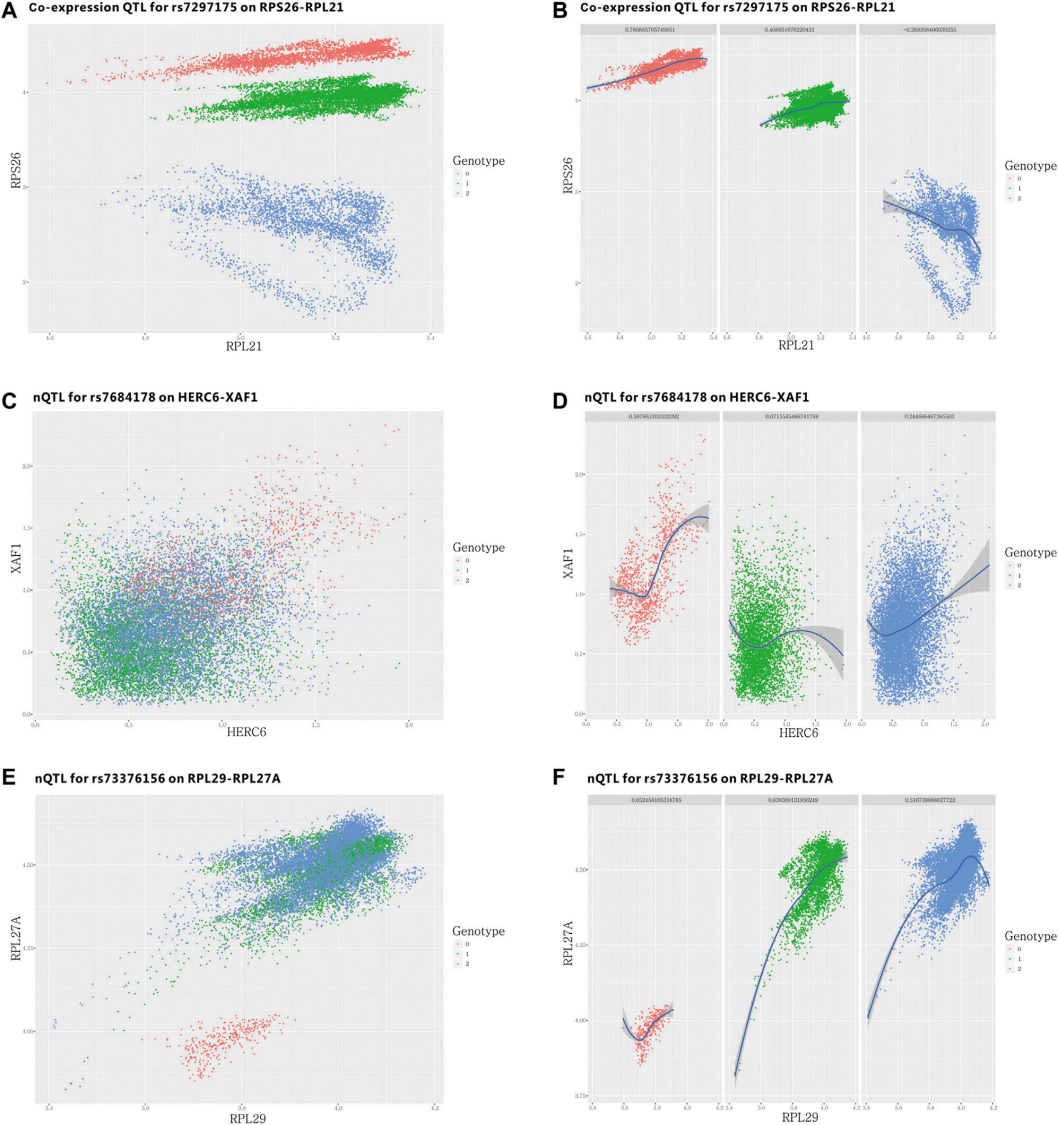
SNP–edge associations (edge traits) detected unsupervised by the nQTL framework and their assessments on single-cell data from the human PBMC dataset. **(A**,**B)** Single-cell plot of previously reported SNP and gene-pair association. **(C–F)** Single-cell plot of our newly identified SNP and gene-pair associations.

As our design, the nQTL framework is able to detect the potential association between SNP and edge in an unsupervised manner. In fact, the strategy of one work to find “co-expression QTL” is directly using single-cell data to calculate the co-expression of a gene-pair (van der Wijst et al., 2018); however, it would not always be applicable in many applications due to there being no individual-specific single-cell data available. Actually, the original work reported only one detailed case of co-expression QTL, involving a gene-pair RPS26 and RPL21. In Figures 7A,B and Supplementary Figure S37A, they have differential co-expression corresponding to a particular SNP type. But it is obvious that these cells have distinguishing expression differences for different SNP types on gene RPS26; thus, this originally reported case would be one of the expected co-expression QTLs; however, it also dominated in conventional differential expression. By contrast, we selected two genes with the most significant association to one SNP according to our nQTL analysis on the given mimic bulk expression data, and we could also observe or validate their potential co-expression difference on unseen single-cell data (Figures 7C–F and Supplementary Figure S37B). Motivated by this idea, we rescreened the gene-pairs corresponding to edge traits from our nQTL analysis on the mimic bulk expression data and further assessed their conditional co-expressions on the single-cell data from different groups of samples. As summarized in Supplementary Figure S38, nQTL analysis indeed can detect many similar “co-expression QTLs” without the supervised information from single-cell data, benefiting from the SSN approach.

## Discussion

**FIGURE 8:**
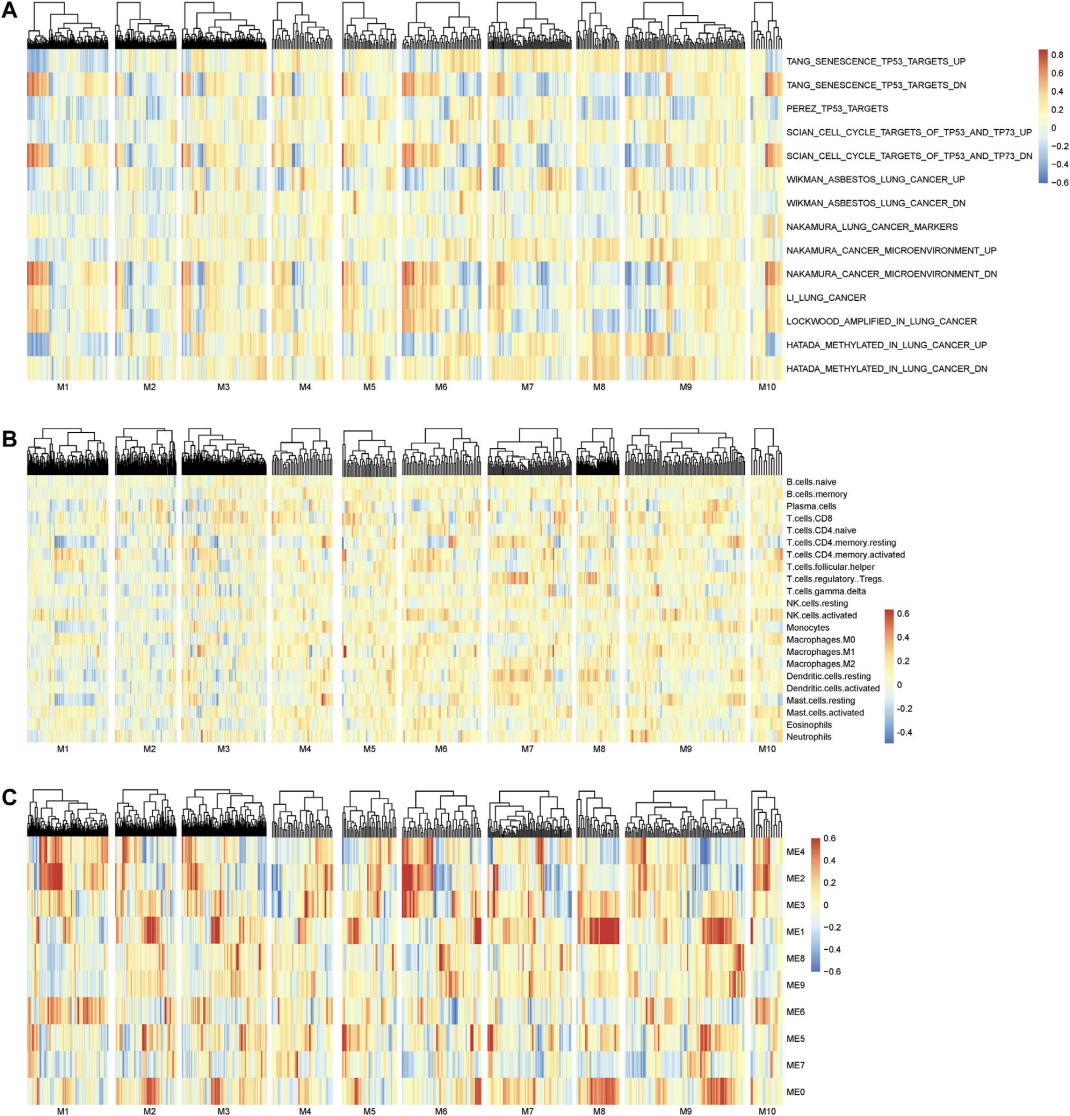
Association analysis on the nQTL module and different factors. **(A)** The association between the nQTL module and (GSVA) pathway enrichment/activity. **(B)** The association between the nQTL module and (CIBERSORT) cell composition. **(C)** The association between the nQTL module and (WGCNA) co-expression module. A few nQTL modules are associated with or driven by potential biological mechanisms, e.g., pathway function, cell composition, or gene co-expression. And many other nQTL modules should be new ones that are determined by particular genotypes through biological network rewiring, according to their GO enrichment and network association.

The main merit of nQTL analysis is to capture the association of genotype and (molecular) phenotype on a sample-specific network level rather than a population level, which is different from previous methods including “co-expression QTLs” (van der Wijst et al., 2018) and “context-specific eQTL” (Zhernakova et al., 2017). Of course, a feature of nQTL would reflect the association between genotype and network activity. As already known, the inference of network activity from its member genes’ expressions would be an option when the experimental measurements of network activity are not directly available at present, which could be similarly used to build the association between genotype and network activity in the nQTL framework. Meanwhile, how to accurately infer the network activity is still another open question, which would be further investigated in our future work. With such an assumption, we aim to provide a proof-of-principle case study on lung cancer data, by associating the genes’ expressions from the nQTL module to sample-specific pathway enrichments from GSVA (Hanzelmann et al., 2013). As shown in Figure 8A, nQTL modules indeed can identify several networks altered by the genetic variants that are associated with a disease (e.g., subtype discriminations) and also be able to translate into upregulation or downregulation of a network/pathway (e.g., lung-cancer-related pathways).

And a previous work (Zhernakova et al., 2017) found that the different gene modules are not driven by individual biological networks but result in differences in cell type composition between the different studied samples. Thus, we have carried out additional investigation on the relationship between nQTL module and cell type composition. Mainly on the lung cancer data as a case study, we used CIBERSORT (Newman et al., 2015) to obtain the potential cell type composition of each sample and analyzed the associations between nQTL module gene expressions and cell type compositions. Indeed, there are a few module genes which are associated with a few cell types (Figure 8B), like naive CD8 or CD4 T cells. Thus, the nQTL framework can detect some modules similar to previous findings, which are partially driven/explained by cell type compositions. However, the nQTL framework is also able to find many new modules that cannot be simply explained by cell type compositions. Thus, cell type composition differences might act as a biomarker, worthy of future study.

Different from conventional co-expression-based modules, the nQTL module captures the genotype-associated co-expressions. It is possible that a transcriptional factor (TF) is an important mediator or regulatory driver between the genotype and molecular phenotype; thus, we have investigated the potential TF and its motifs for nQTL modules on the lung cancer data, by g:Profiler (Raudvere et al., 2019). We found that module1, module5, and module10 would be enriched by the E2F family, AHR, and NFR-1, respectively. Thus, nQTL modules are actually different from conventional co-expression modules like the WGCNA module (Figure 8C), and its relation with TF can be further detected directly by combining more experimental data including Hi-C and ATAC data. Of note, in these co-expression-based methods, genes within the same pathway or genes with a PPI are assumed to be co-expressed; however, their associations would be not necessarily related to particular genotypes. To avoid potential bias in pathway enrichment from co-expression, we could carry out multiple testing corrections for enriched pathways from multiple nQTL modules, and our findings are still consistent in this study (e.g., almost all enrichment *p*-values are less than the corrected threshold of 0.05/10, as shown in Supplementary Figures S8, S20, and S29).

It is well known that GWAS has provided many candidate loci for human phenotypes (e.g., complex diseases). However, more and more reports show that the efficiency of GWAS is limited due to the unknown functions of most recommended loci. Thus, the eQTL analysis is further adopted to investigate the reasonability of the locus by linking the locus’ mutation type and gene expression type so as to introduce the molecular phenotype to explain the functional relevance of particular GWAS-suggested locus. Obviously, this is an integration strategy, which can combine the GWAS summary results and eQTL quantitative results together by the same candidate locus, e.g., SNPs. However, it is still limited by the loss of sample match information, and this means the risk to overlook individual specificity. By contrast, nQTL analysis can avoid this issue by integrating the multiple omics information from the same group of samples and introducing the sample-specific network to link the individual specific genotypes to networks and further to phenotypes, thus improving the genotype detection and functional explanation associated with the target phenotype. For instance, the summary-data-based Mendelian randomization analysis (Zhu et al., 2016) or Bayesian statistical framework (He et al., 2013) can be similarly used to integrate the GWAS summary data and nQTL analysis outcomes. Besides, the joint matrix decomposition model (Hu et al., 2020) is also an alternative approach for integrating GWAS and nQTL associations on the basis of their shared SNPs. Indeed, the identification of nQTL and GWAS combination with new knowledge of a common disease is worth studying in the future.

From a technical viewpoint, the key of nQTL analysis is to obtain the individual network for one sample from gene expression data. As well known to us, reconstructing a network generally requires multiple samples to estimate the associations between variables of the network, where the variables are represented as nodes/genes in terms of expression in a network and the variable associations are represented as edges/gene-pairs in terms of correlation/strength in the same network. Not only nQTL analysis but also other approaches in biological and biomedical studies are facing the problem of how to estimate the network (or its feature) when only one sample is available for a target phenotype. To address this issue, a few sample-specific network construction methods have been developed and applied. Differential edge-like transformation (DET) based on single-sample PCC (sPCC) has been developed by using the additivity of PCC on multiple samples and assigning the individual additive factors to each sample (Zeng et al., 2014), which can estimate the significant co-expression of two variables compared to the population average. Particularly, the mean of DET between two variables from a group of samples equals the PCC of this variable-pair on these samples. Meanwhile, the SSN method constructs the sample-specific differential network of one sample against a set of reference samples (Liu et al., 2016; Yu et al., 2017), which develops a statistic on the co-expression change when one sample is added into the reference samples. And the CSN focuses on the inference of network representation for each single cell from scRNA-seq data by transforming the data from an “unstable” gene expression form to a “stable” gene association form based on statistical independence (Dai et al., 2019). Besides, the Linear Interpolation to Obtain Network Estimates for Single Samples (LIONESS) reconstructs the individual specific network in a population of samples for each detailed sample, by calculating the correlation statistical significance between all samples and the samples without a given single sample (Kuijjer et al., 2019). In fact, the model of nQTL is comprehensive, and these sample-specific network construction methods are alternatives for it. Considering that the conventional eQTL model and its biological question always focus on the linear association among population samples, the DET approach is adopted in this nQTL study. The integration of other methods such as SSN, CSN, and LIONESS is worthy of future evaluation, dependent on biological background and questions in research context. In addition to enriched genes and their associations within prior-known networks/contexts in the current nQTL analysis, using network reconstruction or prediction can further improve the power of nQTL analysis.

Different from GWAS/eQTL applications, nQTL analysis has great potential in studying many complex biological problems and provides new insights from a system/network viewpoint, e.g., individual specific association discovery on diverse cell types based on new single-cell technologies (van der Wijst et al., 2018; Camp et al., 2019). As known, using nQTL or eQTL analyses for the integration of omics data at multilevels requires multilevel data from the same experimental subject in concept. For example, in the conventional study, we required the SNP and expression data for the same person in eQTL analysis although the test sample might be from different locations or times for this person, and this kind of data is well known as matched-data organization. However, it is hard to obtain such multilevel data for the same cell with current single-cell technologies. Thus, we have to approximately apply nQTL or eQTL analysis or a similar integrative model in single-cell studies currently. It is necessary to develop integrative omics and analysis methods in focal research of wide single-cell fields. Of note, the potential extension of the nQTL concept and model on the integration of multi-omics data would still be burdened by correlations among and within the different omics layers. Although this method could help identify causal relations within and between omics layers, there are still many issues that need to be further studied, such as causality inference (Leng et al., 2020).

As a unified framework, our nQTL approach provides a systematical tool to support the whole analysis pipeline to detect the edge traits, edge signatures, and their relationship with phenotypes, which builds an association cascade from genotype to network and further to phenotype. Compared to conventional eQTL or GWAS analysis, nQTL analysis not only supplies the potential phenotype drivers corresponding to genotypes but also reveals functional insights of these relevant genotypes in the form of global network and local modules. Therefore, the development and adoption of advanced network inference methods and module decomposition approaches would benefit nQTL research by promoting the efficiency and robustness of analysis.

Of note, the quality control and significance correction should be further promoted for nQTL analysis. In these experiments, the used public datasets have no minor allele frequency (MAF) available, and thus, the MAF filter was not used further. In future studies with a focus on particular variants like GWAS outcomes, an nQTL study could use a MAF filter to narrow down the genotype analysis. Besides, in addition to the FDR used by MatrixEQTL, the empirical FDR adopted by “co-expression QTLs” (van der Wijst et al., 2018) and “context-specific eQTL” (Zhernakova et al., 2017) analyses was also calculated, where there are no result changes on the simulated data; and the empirical FDR is also low under the current experimental threshold setting in real datasets (see Supplementary Table S12). Indeed, the combination of different permutation strategies for significance correction should provide reliable information on the confidence of identified eQTLs and nQTLs.

## Conclusion

Collectively, the nQTL framework is proposed to detect the association cascade: genotype → network → phenotype, which not only interprets the biological or biomedical significance of the discovered nQTL traits or features but also reveals the detailed function roles of those nQTLs as network determinates associated with phenotypes. Our multiple simulation studies and case studies on human healthy and disease phenotypes all support the efficiency of the nQTL framework, which can identify not only network traits for analyzing complex biological processes at a network level but also network signatures for phenotype discrimination.

## Data Availability

The used data are from public databases like GEO (https://www.ncbi.nlm.nih.gov/geo/) and CCLE (https://portals.broadinstitute.org/ccle/). The nQTL codes are available in the GitHub repository, https://github.com/ztpub/nQTL, and other analysis scripts and data can be required from the corresponding author.

## Abbreviations

CCLE, Cancer Cell Line Encyclopedia; eQTL, expression quantitative trait loci; GWAS, genome-wide association study; nQTL, network quantitative trait loci; NSCLC, non-small-cell lung cancer; PBMC, peripheral blood mononuclear cell; PLSDA, partial least squares discriminant analysis; PPI, protein–protein interaction; SCLC, small-cell lung cancer; SNP, single-nucleotide polymorphisms; SSN, single-sample network; TF, transcriptional factor.
