# Supplementary material for: Interpreting Functional Impact of Genetic Variations by Network QTL for Genotype–Phenotype Association Study
Source: Front Cell Dev Biol. 2022 Jan 26;9:720321. doi: 10.3389/fcell.2021.720321 (PMC8826544; doi:10.3389/fcell.2021.720321)
Supplement: Supplementary file 1 [file DataSheet1.ZIP › SI-figure/Figure S1.pdf]

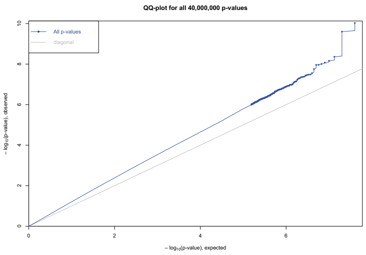
QQ-plot for all 40,000,000 p-values
All p-values
diagonal
− log10(p-value), observed
− log10(p-value), expected

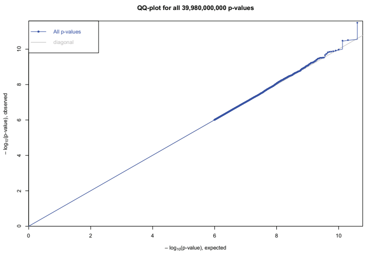
QQ-plot for all 39,980,000,000 p-values
All p-values
diagonal
− log10(p-value), observed
− log10(p-value), expected

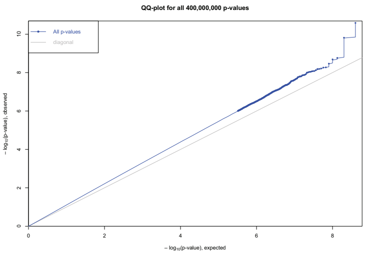
QQ-plot for all 400,000,000 p-values
All p-values
diagonal
− log10(p-value), observed
− log10(p-value), expected

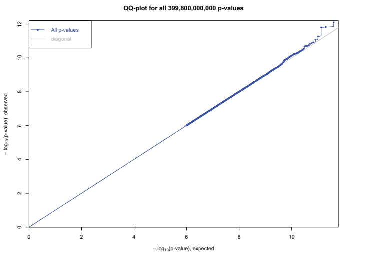
QQ-plot for all 399,800,000,000 p-values
All p-values
diagonal
− log10(p-value), observed
− log10(p-value), expected

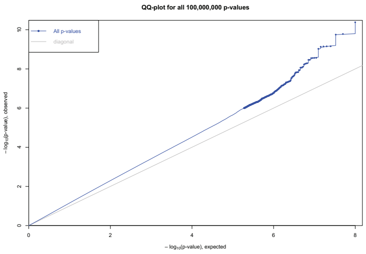
QQ-plot for all 100,000,000 p-values
All p-values
diagonal
− log10(p-value), observed
− log10(p-value), expected

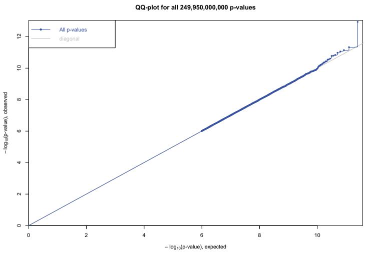
QQ-plot for all 249,950,000,000 p-values
All p-values
diagonal
− log10(p-value), observed
− log10(p-value), expected

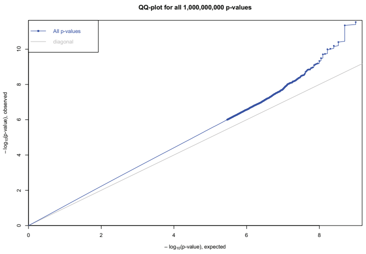
QQ-plot for all 1,000,000,000 p-values
All p-values
diagonal
− log10(p-value), observed
− log10(p-value), expected

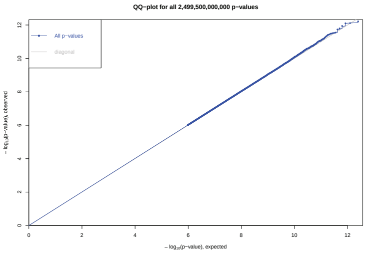
QQ−plot for all 2,499,500,000,000 p−values
All p−values
diagonal
− log10(p-value), observed
− log10(p-value), expected

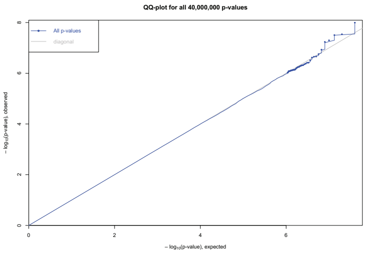
QQ-plot for all 40,000,000 p-values
All p-values
diagonal
− log10(p-value), observed
− log10(p-value), expected

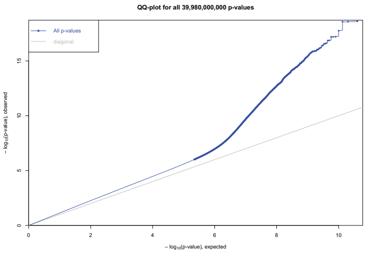
QQ-plot for all 39,980,000,000 p-values
All p-values
diagonal
− log10(p-value), observed
− log10(p-value), expected

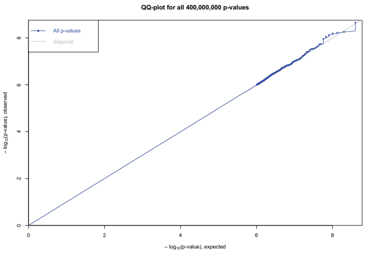
QQ-plot for all 400,000,000 p-values
All p-values
diagonal
− log10(p-value), observed
− log10(p-value), expected

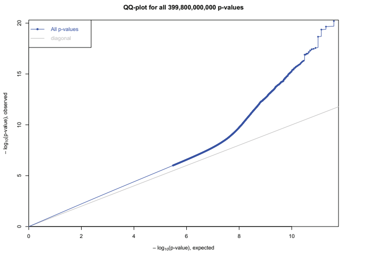
QQ-plot for all 399,800,000,000 p-values
All p-values
diagonal
− log10(p-value), observed
− log10(p-value), expected

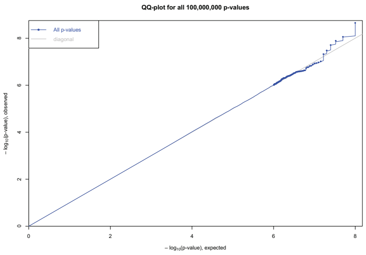
QQ-plot for all 100,000,000 p-values
All p-values
diagonal
− log10(p-value), observed
− log10(p-value), expected

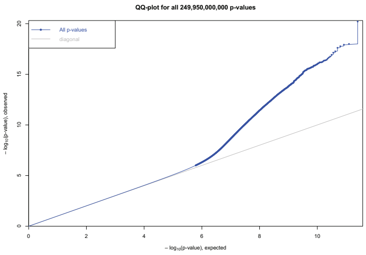
QQ-plot for all 249,950,000,000 p-values
All p-values
diagonal
− log10(p-value), observed
− log10(p-value), expected

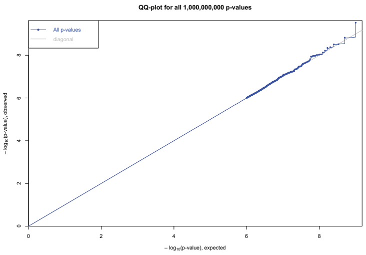
QQ-plot for all 1,000,000,000 p-values
All p-values
diagonal
− log10(p-value), observed
− log10(p-value), expected

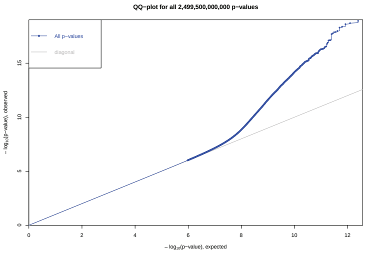
QQ−plot for all 2,499,500,000,000 p−values
All p−values
diagonal
− log10(p-value), observed
− log10(p-value), expected
