# Supplementary material for: Interpreting Functional Impact of Genetic Variations by Network QTL for Genotype–Phenotype Association Study
Source: Front Cell Dev Biol. 2022 Jan 26;9:720321. doi: 10.3389/fcell.2021.720321 (PMC8826544; doi:10.3389/fcell.2021.720321)
Supplement: Supplementary file 1 [file DataSheet1.ZIP › SI-figure/Figure S13.pdf]

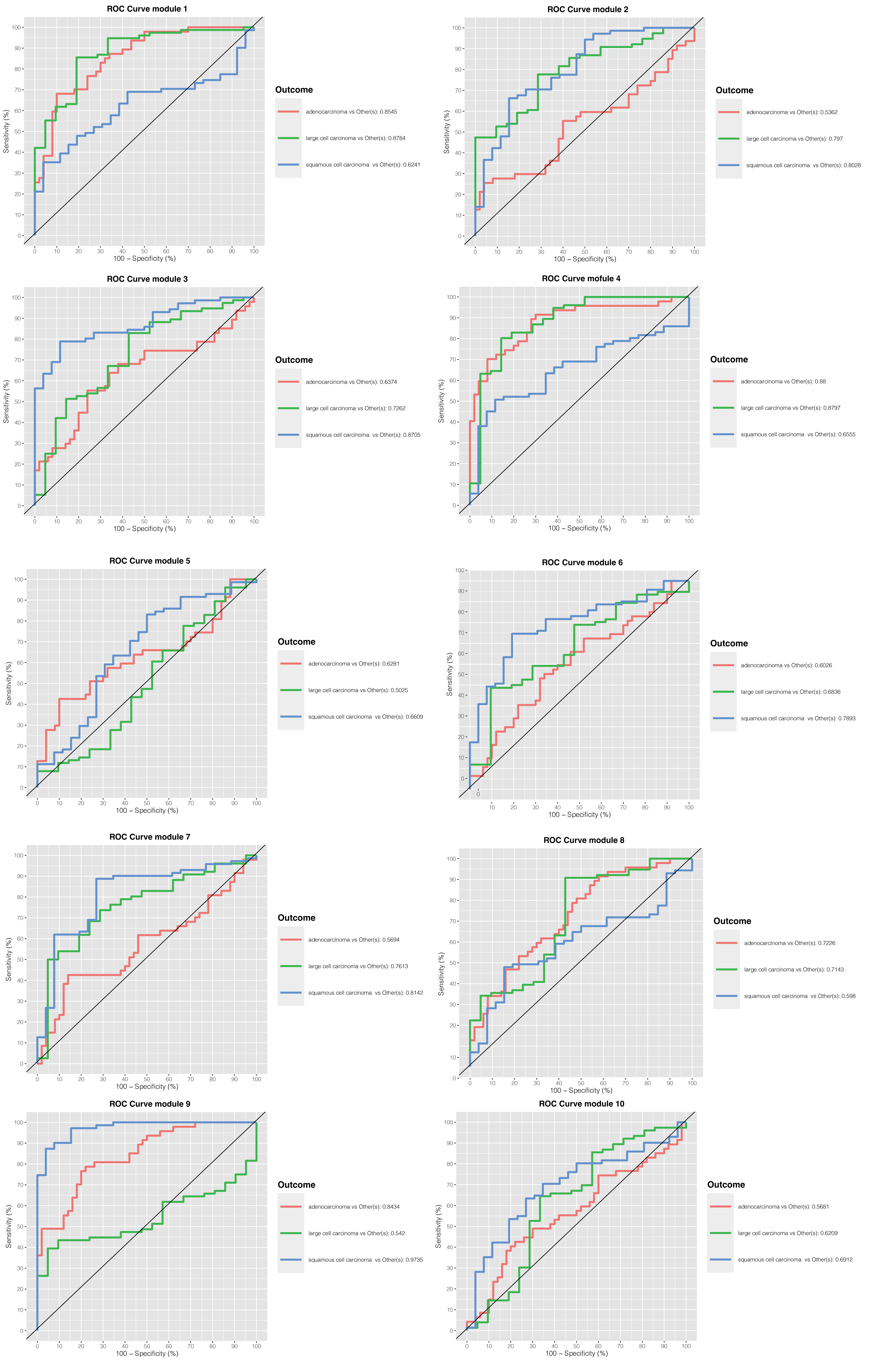
ROC Curve module 1
Outcome
adenocarcinoma vs Other(s): 0.8545
large cell carcinoma vs Other(s): 0.8784
squamous cell carcinoma vs Other(s): 0.6241
ROC Curve module 2
Outcome
adenocarcinoma vs Other(s): 0.5362
large cell carcinoma vs Other(s): 0.797
squamous cell carcinoma vs Other(s): 0.8028
ROC Curve module 3
Outcome
adenocarcinoma vs Other(s): 0.6374
large cell carcinoma vs Other(s): 0.7262
squamous cell carcinoma vs Other(s): 0.8705
ROC Curve mofule 4
Outcome
adenocarcinoma vs Other(s): 0.88
large cell carcinoma vs Other(s): 0.8797
squamous cell carcinoma vs Other(s): 0.6555
ROC Curve module 5
Outcome
adenocarcinoma vs Other(s): 0.6281
large cell carcinoma vs Other(s): 0.5025
squamous cell carcinoma vs Other(s): 0.6609
ROC Curve module 6
Outcome
adenocarcinoma vs Other(s): 0.6026
large cell carcinoma vs Other(s): 0.6836
squamous cell carcinoma vs Other(s): 0.7893
ROC Curve module 7
Outcome
adenocarcinoma vs Other(s): 0.5694
large cell carcinoma vs Other(s): 0.7613
squamous cell carcinoma vs Other(s): 0.8142
ROC Curve module 8
Outcome
adenocarcinoma vs Other(s): 0.7226
large cell carcinoma vs Other(s): 0.7143
squamous cell carcinoma vs Other(s): 0.598
ROC Curve module 9
Outcome
adenocarcinoma vs Other(s): 0.8434
large cell carcinoma vs Other(s): 0.542
squamous cell carcinoma vs Other(s): 0.9735
ROC Curve module 10
Outcome
adenocarcinoma vs Other(s): 0.5681
large cell carcinoma vs Other(s): 0.6209
squamous cell carcinoma vs Other(s): 0.6912
Sensitivity (%)
100 – Specificity (%)
