# Supplementary material for: Interpreting Functional Impact of Genetic Variations by Network QTL for Genotype–Phenotype Association Study
Source: Front Cell Dev Biol. 2022 Jan 26;9:720321. doi: 10.3389/fcell.2021.720321 (PMC8826544; doi:10.3389/fcell.2021.720321)
Supplement: Supplementary file 1 [file DataSheet1.ZIP › SI-figure/Figure S14.pdf]

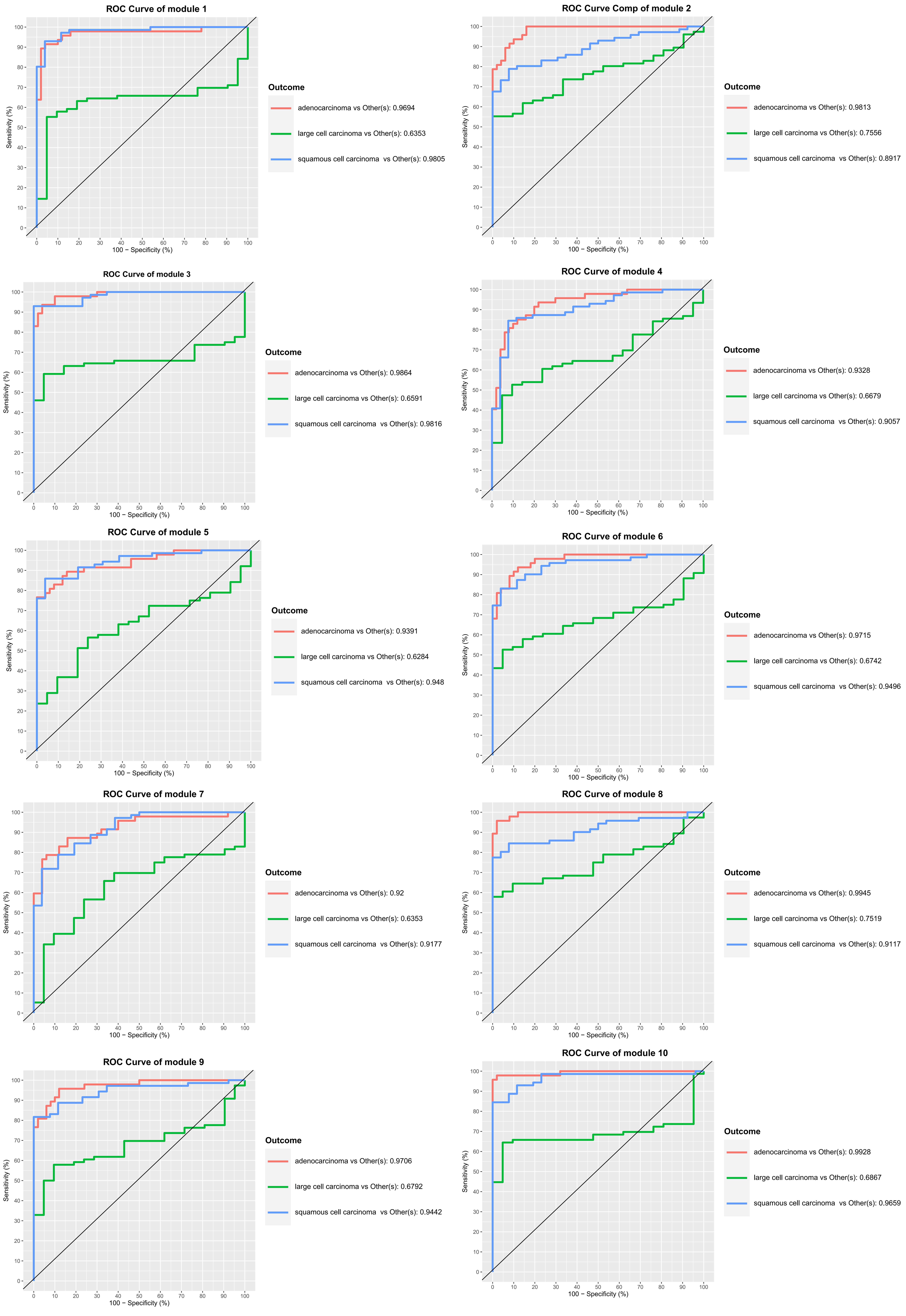
ROC Curve of module 1
Sensitivity (%)
100 − Specificity (%)
Outcome
adenocarcinoma vs Other(s): 0.9694
large cell carcinoma vs Other(s): 0.6353
squamous cell carcinoma vs Other(s): 0.9805
ROC Curve Comp of module 2
Sensitivity (%)
100 − Specificity (%)
Outcome
adenocarcinoma vs Other(s): 0.9813
large cell carcinoma vs Other(s): 0.7556
squamous cell carcinoma vs Other(s): 0.8917
ROC Curve of module 3
Sensitivity (%)
100 − Specificity (%)
Outcome
adenocarcinoma vs Other(s): 0.9864
large cell carcinoma vs Other(s): 0.6591
squamous cell carcinoma vs Other(s): 0.9816
ROC Curve of module 4
Sensitivity (%)
100 − Specificity (%)
Outcome
adenocarcinoma vs Other(s): 0.9328
large cell carcinoma vs Other(s): 0.6679
squamous cell carcinoma vs Other(s): 0.9057
ROC Curve of module 5
Sensitivity (%)
100 − Specificity (%)
Outcome
adenocarcinoma vs Other(s): 0.9391
large cell carcinoma vs Other(s): 0.6284
squamous cell carcinoma vs Other(s): 0.948
ROC Curve of module 6
Sensitivity (%)
100 − Specificity (%)
Outcome
adenocarcinoma vs Other(s): 0.9715
large cell carcinoma vs Other(s): 0.6742
squamous cell carcinoma vs Other(s): 0.9496
ROC Curve of module 7
Sensitivity (%)
100 − Specificity (%)
Outcome
adenocarcinoma vs Other(s): 0.92
large cell carcinoma vs Other(s): 0.6353
squamous cell carcinoma vs Other(s): 0.9177
ROC Curve of module 8
Sensitivity (%)
100 − Specificity (%)
Outcome
adenocarcinoma vs Other(s): 0.9945
large cell carcinoma vs Other(s): 0.7519
squamous cell carcinoma vs Other(s): 0.9117
ROC Curve of module 9
Sensitivity (%)
100 − Specificity (%)
Outcome
adenocarcinoma vs Other(s): 0.9706
large cell carcinoma vs Other(s): 0.6792
squamous cell carcinoma vs Other(s): 0.9442
ROC Curve of module 10
Sensitivity (%)
100 − Specificity (%)
Outcome
adenocarcinoma vs Other(s): 0.9928
large cell carcinoma vs Other(s): 0.6867
squamous cell carcinoma vs Other(s): 0.9659
