# Supplementary material for: Interpreting Functional Impact of Genetic Variations by Network QTL for Genotype–Phenotype Association Study
Source: Front Cell Dev Biol. 2022 Jan 26;9:720321. doi: 10.3389/fcell.2021.720321 (PMC8826544; doi:10.3389/fcell.2021.720321)
Supplement: Supplementary file 1 [file DataSheet1.ZIP › SI-figure/Figure S15.pdf]

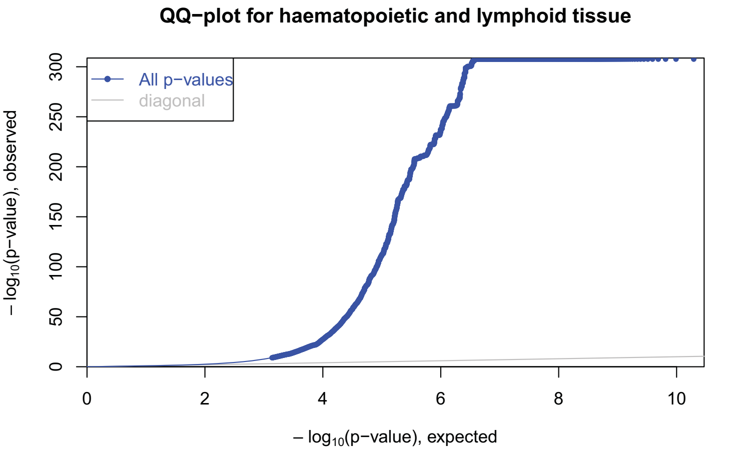
QQ−plot for haematopoietic and lymphoid tissue
All p−values
diagonal
$-\log_{10}$(p−value), observed
0
50
100
150
200
250
300
0
2
4
6
8
10
$-\log_{10}$(p−value), expected

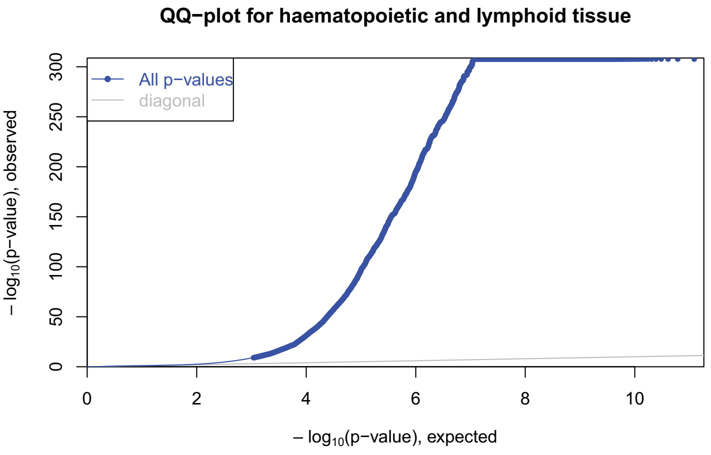
QQ−plot for haematopoietic and lymphoid tissue
All p−values
diagonal
$-\log_{10}$(p−value), observed
0
50
100
150
200
250
300
0
2
4
6
8
10
$-\log_{10}$(p−value), expected
