# Supplementary material for: Interpreting Functional Impact of Genetic Variations by Network QTL for Genotype–Phenotype Association Study
Source: Front Cell Dev Biol. 2022 Jan 26;9:720321. doi: 10.3389/fcell.2021.720321 (PMC8826544; doi:10.3389/fcell.2021.720321)

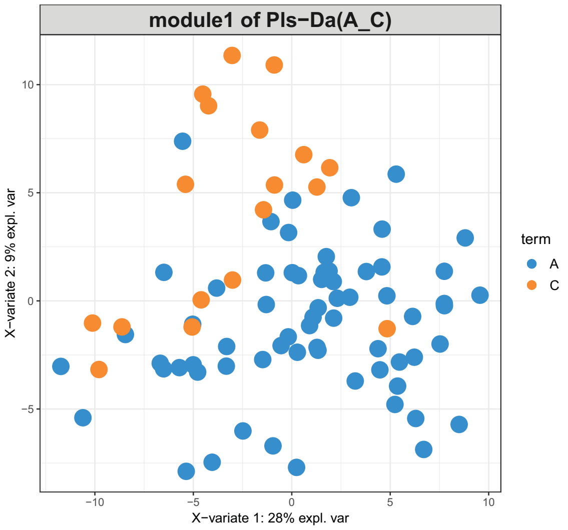
module1 of Pls−Da(A_C)
X−variate 1: 28% expl. var
X−variate 2: 9% expl. var
term
A
C

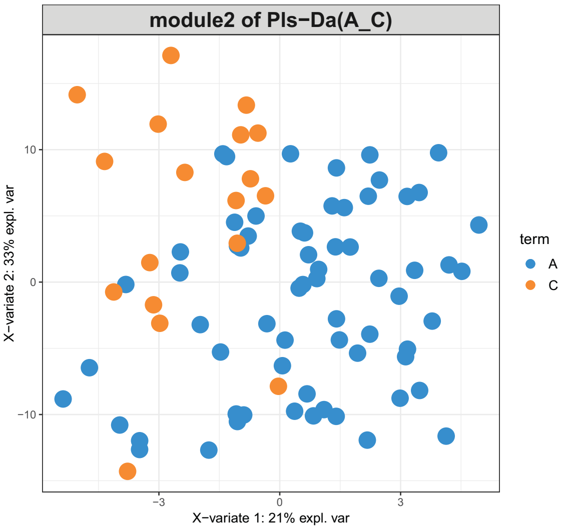
module2 of Pls−Da(A_C)
X−variate 1: 21% expl. var
X−variate 2: 33% expl. var
term
A
C

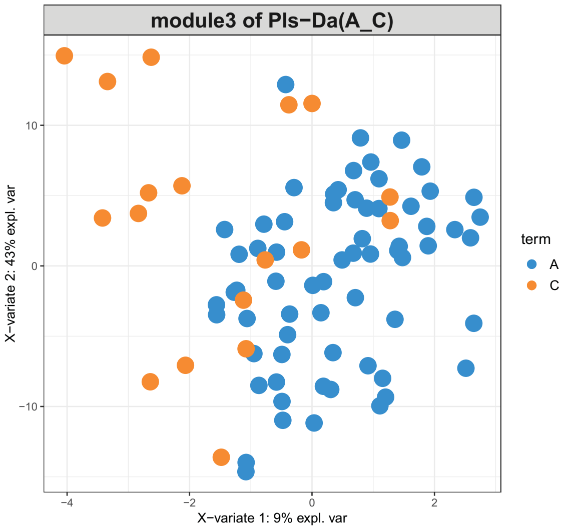
module3 of Pls−Da(A_C)
X−variate 1: 9% expl. var
X−variate 2: 43% expl. var
term
A
C

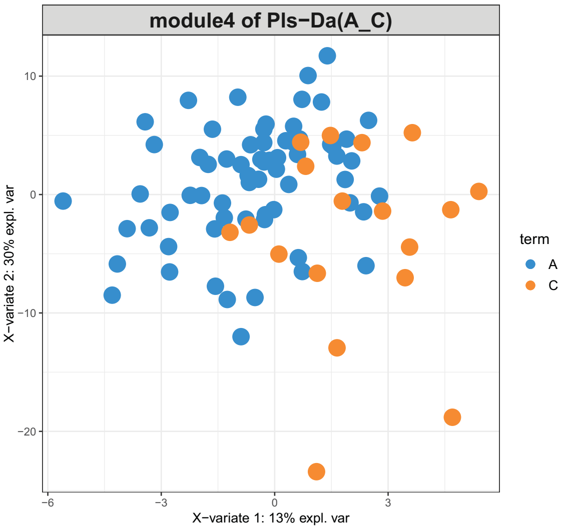
module4 of Pls−Da(A_C)
X−variate 1: 13% expl. var
X−variate 2: 30% expl. var
term
A
C

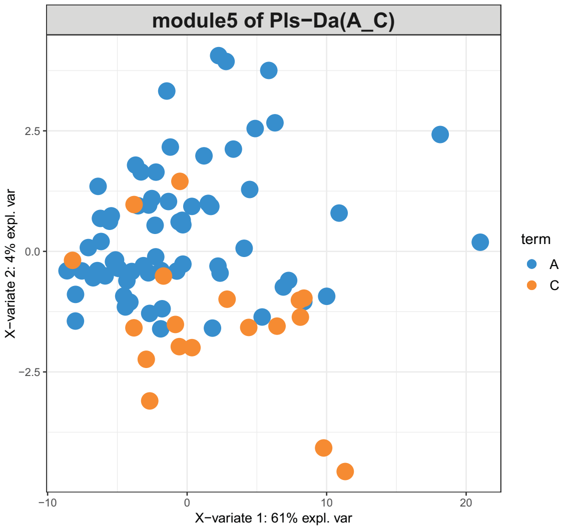
module5 of Pls−Da(A_C)
X−variate 1: 61% expl. var
X−variate 2: 4% expl. var
term
A
C

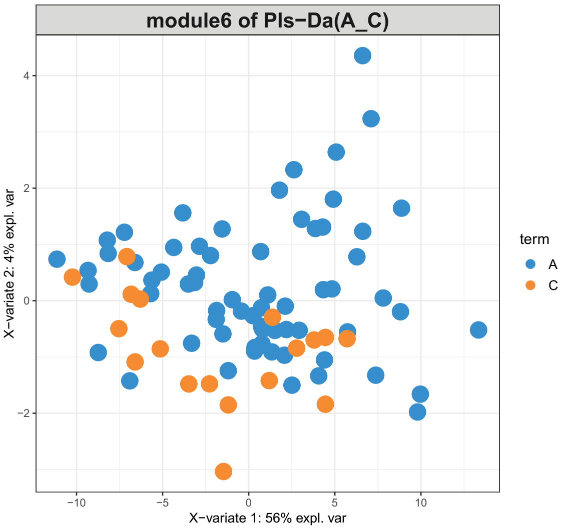
module6 of Pls−Da(A_C)
X−variate 1: 56% expl. var
X−variate 2: 4% expl. var
term
A
C

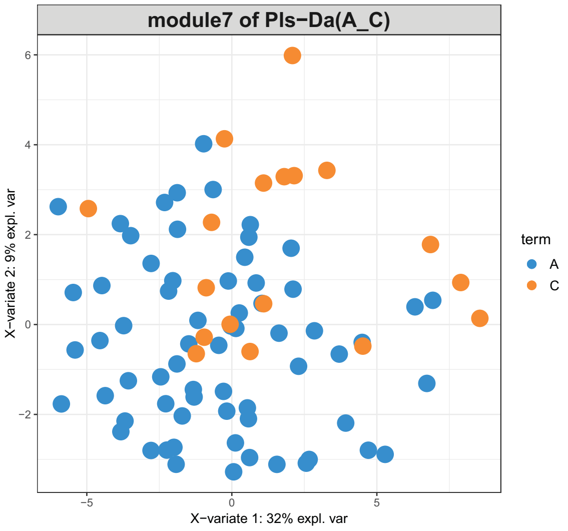
module7 of Pls−Da(A_C)
X−variate 1: 32% expl. var
X−variate 2: 9% expl. var
term
A
C

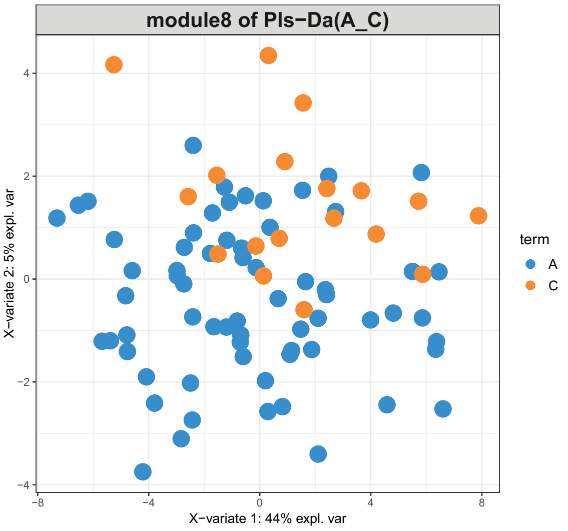
module8 of Pls−Da(A_C)
X−variate 1: 44% expl. var
X−variate 2: 5% expl. var
term
A
C

CENSORED:SURVIVAL_DAYS
ER1|RPA1|PNOC|POLR1B|CNOT6|POLD1|NTPCR|CDK2|CCNB2|POLR2G|SNX2|DTL|EIF4B
Concordance Index = 96.41, Log-Rank Equal Curves p=5.329e-15, R^2=0.553/0.876
Risk Groups Hazard Ratio = 41.32 (conf. int. 9.74 ~ 175.2), p=4.45e-07

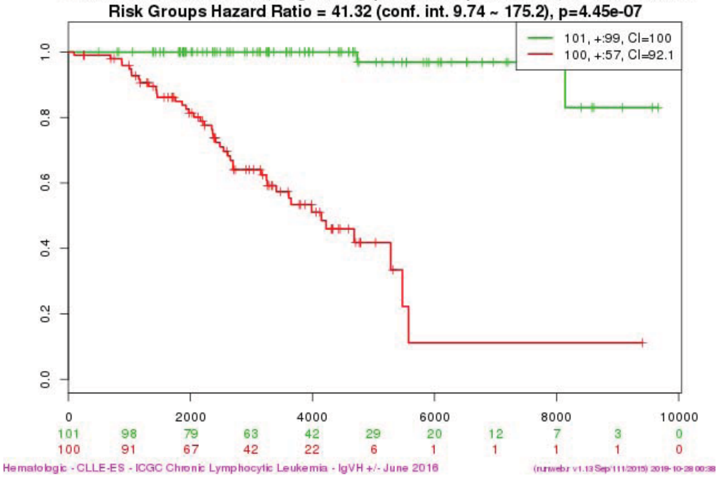

CENSORED:SURVIVAL_DAYS
DT1|RGR|TOR4A|CHRM2|GALR1|ANAPC11|KNG1|HTR1E|CBL|NPY5R|PFKL|RAC1|MMP14
Concordance Index = 100, Log-Rank Equal Curves p=0, R^2=NaN/0.876
Risk Groups Hazard Ratio = 1.888e+09 (conf. int. 0 ~ Inf), p=0.9956

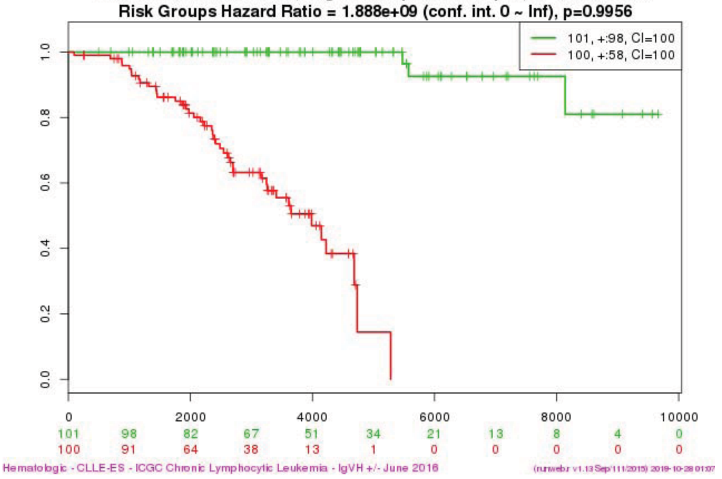

CENSORED:SURVIVAL_DAYS
V1|ACKR3|TDGF1|CNBD2|STAT5A|ALB|IFI30|GRIK2|OAS2|CACNG8|CATSPER4|PCDH7|NP
Concordance Index = 96.97, Log-Rank Equal Curves p=1.643e-11, R^2=0.642/0.876
Risk Groups Hazard Ratio = 25.89 (conf. int. 6.27 ~ 107), p=6.946e-06

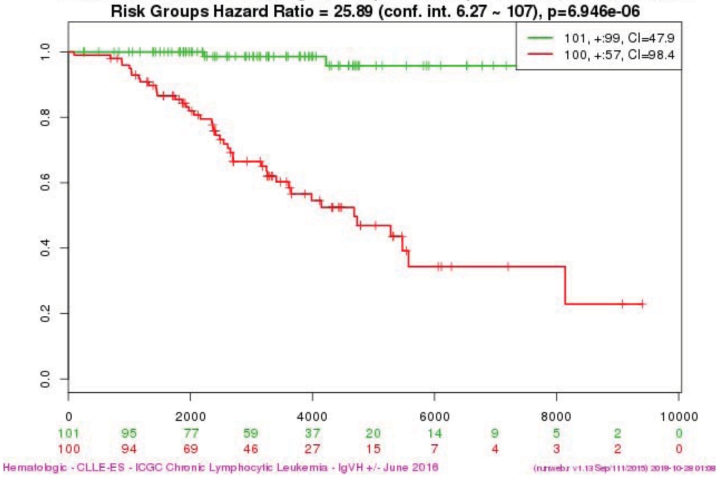

CENSORED:SURVIVAL_DAYS
PN11|GPR17|P2RY1|CATSPERG|SUCNR1|PHACTR2|HTR2A.AS1|SAA1|F2|NPSR1|GAST|AP
Concordance Index = 90.37, Log-Rank Equal Curves p=1.073e-10, R^2=0.512/0.876
Risk Groups Hazard Ratio = 10.89 (conf. int. 4.52 ~ 26.28), p=1.065e-07

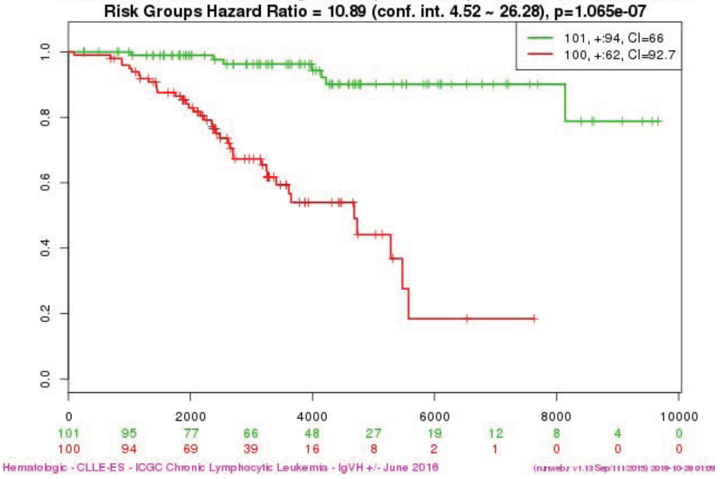

CENSORED:SURVIVAL_DAYS
}|CHRM2|MYBPC1|HTR1F|TMOD3|PLD2|EGF|GNG10|HERC3|PTGS1|MYL5|ALOX15B|GNAL
Concordance Index = 86.53, Log-Rank Equal Curves p=3.009e-14, R^2=0.439/0.876
Risk Groups Hazard Ratio = 23.89 (conf. int. 7.35 ~ 77.58), p=1.292e-07

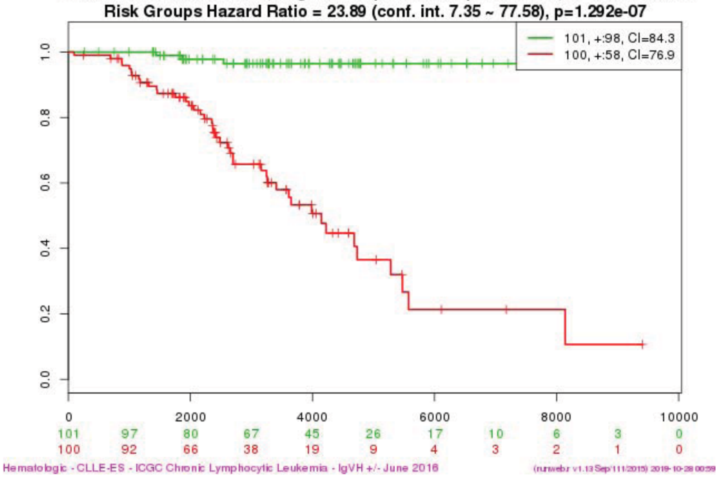

CENSORED:SURVIVAL_DAYS
|GPR17|EPHA1.AS1|PROZ|CASR|CCR9|BRS3|SAA1|NPSR1|HCRTR2|EDN1|GPR26|GNG12.
Concordance Index = 77.62, Log-Rank Equal Curves p=2.482e-05, R^2=0.217/0.876
Risk Groups Hazard Ratio = 4.05 (conf. int. 2 ~ 8.18), p=9.878e-05

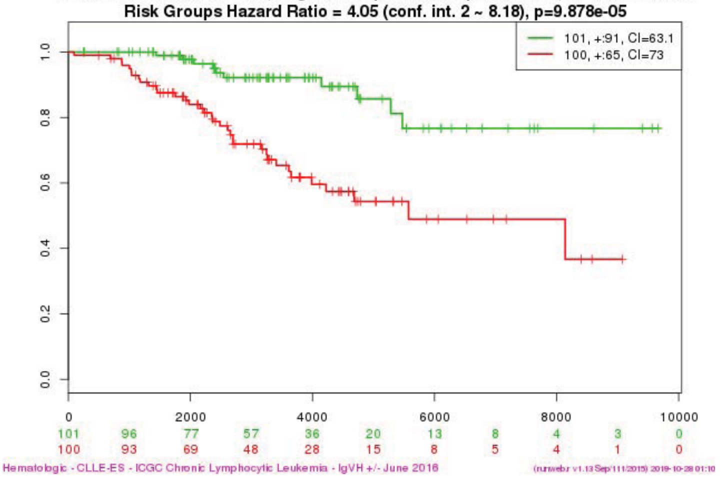

CENSORED:SURVIVAL_DAYS
!1|PTH2R|POLR1D|MPL|F2|DVL3|GRB7|VEGFA|BMX|CEL|ARRB2|TAS2R39|SAA1|CSF2RA|
Concordance Index = 76.31, Log-Rank Equal Curves p=0.000268, R^2=0.231/0.876
Risk Groups Hazard Ratio = 3.3 (conf. int. 1.67 ~ 6.53), p=0.0005866

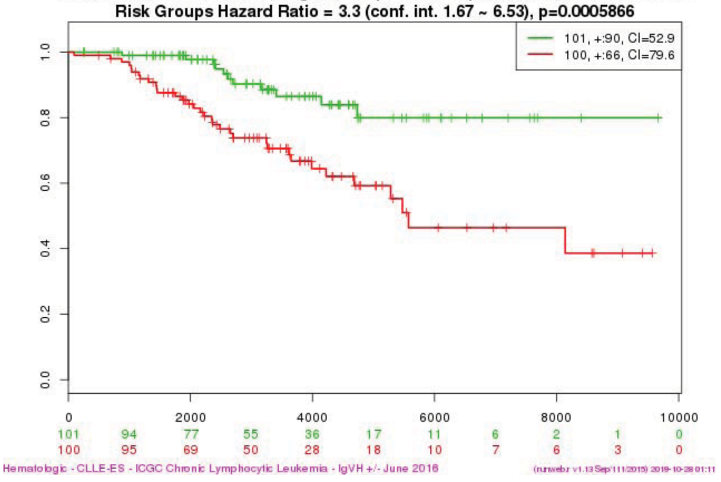

CENSORED:SURVIVAL_DAYS
KNG1|GPR26|HCRTR2|GRK5|NFATC3|F2|HTR2A.AS1|VIPR1|VIP|TKTL1|NPSR1|LGR6|USP9
Concordance Index = 76.55, Log-Rank Equal Curves p=2.898e-07, R^2=0.216/0.876
Risk Groups Hazard Ratio = 5.66 (conf. int. 2.7 ~ 11.88), p=4.547e-06

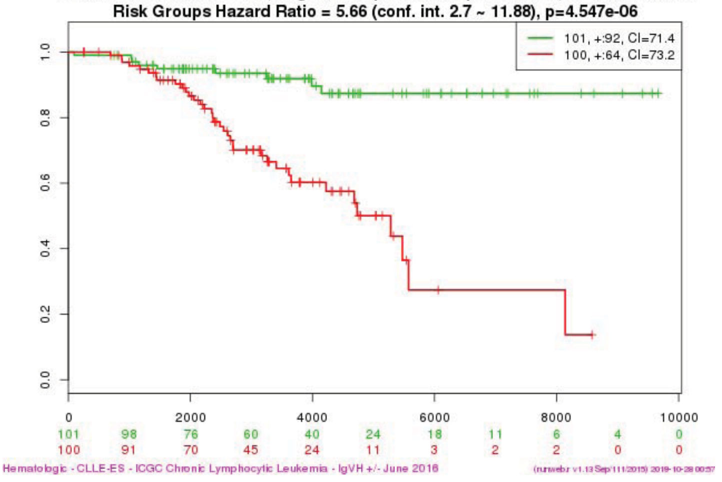

CENSORED:Survival_Months
7|CXCR6|CDC25C|TGFBR2|CXCL5|ADRA2A|UBA7|XRCC1|CHUK|CCR4|CDC2|CDK2|CDK9|
Concordance Index = 64.25, Log-Rank Equal Curves p=2.07e-06, R^2=0.142/0.997
Risk Groups Hazard Ratio = 2.25 (conf. int. 1.59 ~ 3.19), p=4.858e-06

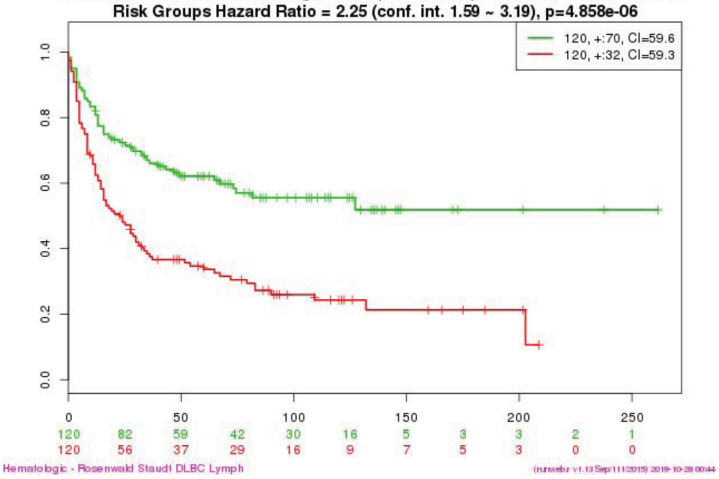

CENSORED:Survival_Months
'NF|UBA7|PIK3CG|IL24|BTK|CD79B|ZNF294|ZFP36L1|MMP14|TKT|PTPN6|CXCL1|MMP17|GI
Concordance Index = 65.52, Log-Rank Equal Curves p=3.039e-06, R^2=0.12/0.997
Risk Groups Hazard Ratio = 2.2 (conf. int. 1.56 ~ 3.11), p=6.649e-06

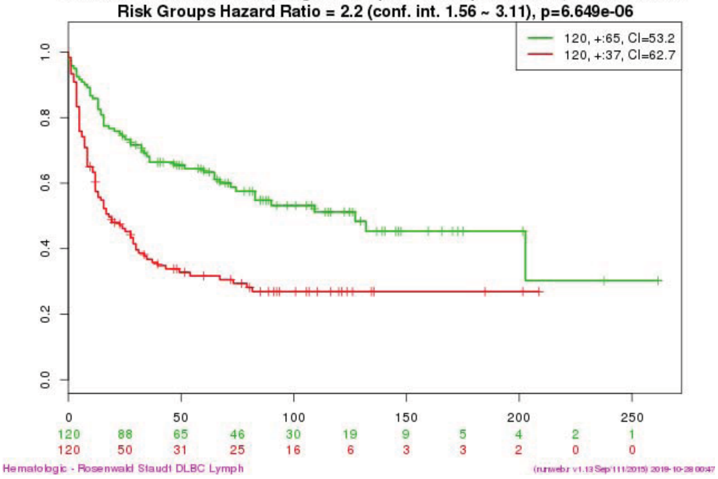

CENSORED:Survival_Months
ADRA2A|CXCL5|IFI30|LHFPL2|GABBR1|CXCL1|MPL
Concordance Index = 60.47, Log-Rank Equal Curves p=0.01746, R^2=0.065/0.997
Risk Groups Hazard Ratio = 1.49 (conf. int. 1.07 ~ 2.09), p=0.0195

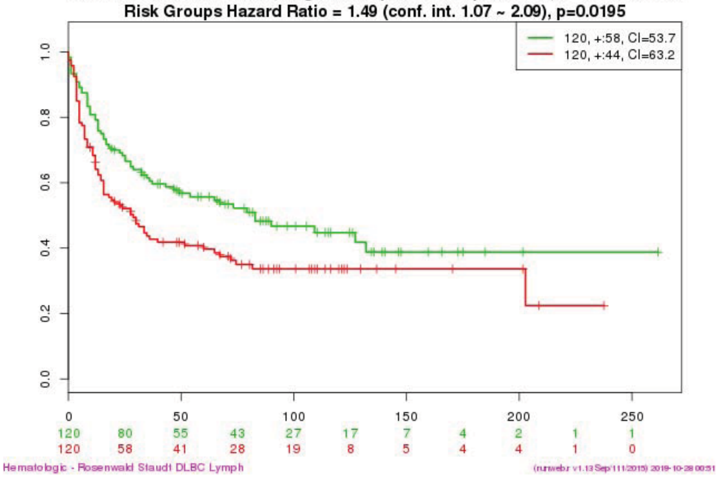

CENSORED:Survival_Months
SELP|MPL|ADRA2A|CDH5|CTR9|ZNF294|GPR4
Concordance Index = 59.45, Log-Rank Equal Curves p=0.004046, R^2=0.044/0.997
Risk Groups Hazard Ratio = 1.63 (conf. int. 1.16 ~ 2.28), p=0.004866

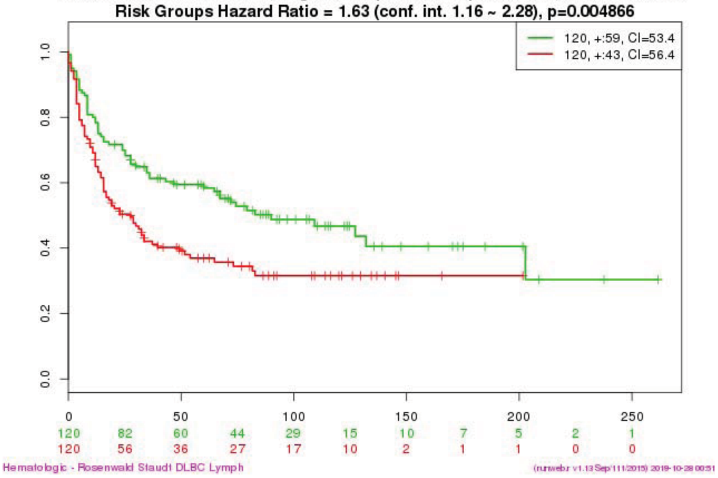

CENSORED:Survival_Months
CYP2B6|CCR4|CYP2J2|MAPK3|PTGS2|CCL11|PAFAH1B1
Concordance Index = 54.93, Log-Rank Equal Curves p=0.836, R^2=0.017/0.997
Risk Groups Hazard Ratio = 1.04 (conf. int. 0.74 ~ 1.45), p=0.8378

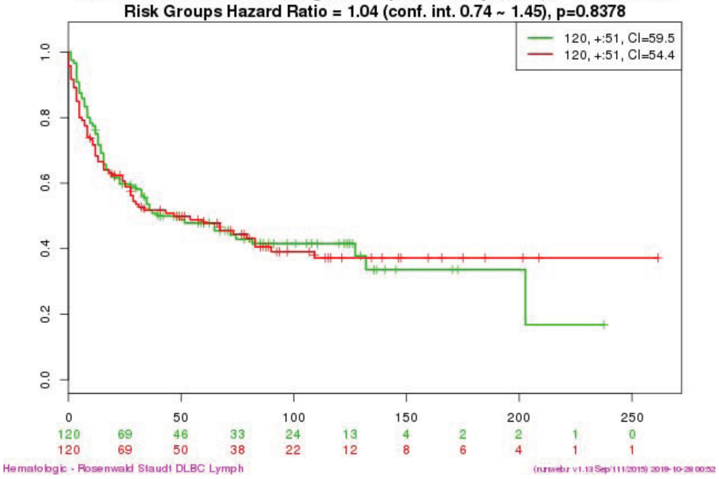

CENSORED:Survival_Months
GNGT2|GPR4|GNB5|EPHA1
Concordance Index = 58.04, Log-Rank Equal Curves p=0.001959, R^2=0.046/0.997
Risk Groups Hazard Ratio = 1.69 (conf. int. 1.2 ~ 2.38), p=0.002462

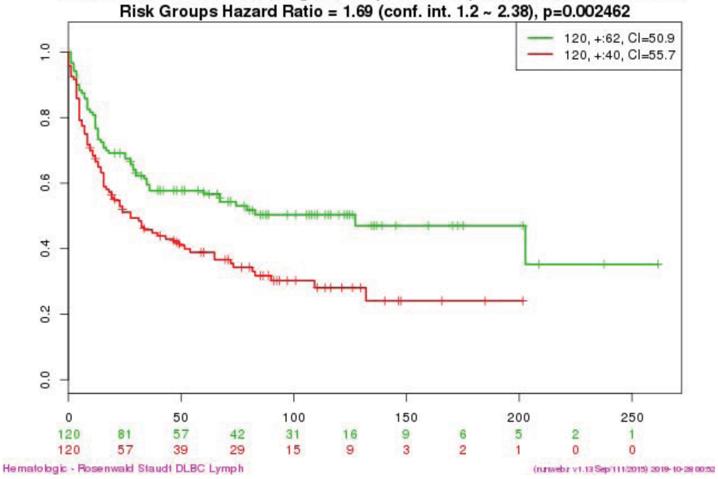

CENSORED:Survival_Months
MPL|HRAS|UBA7|VEGFA|GABBR1
Concordance Index = 55.63, Log-Rank Equal Curves p=0.01905, R^2=0.027/0.997
Risk Groups Hazard Ratio = 1.49 (conf. int. 1.06 ~ 2.08), p=0.02121

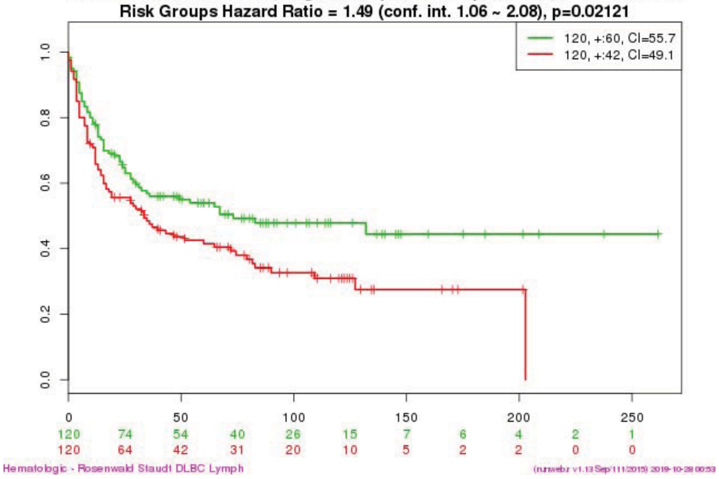

CENSORED:Survival_Months
CD5|GPR4|LYL1
Concordance Index = 56.6, Log-Rank Equal Curves p=0.01986, R^2=0.02/0.997
Risk Groups Hazard Ratio = 1.48 (conf. int. 1.06 ~ 2.07), p=0.02207

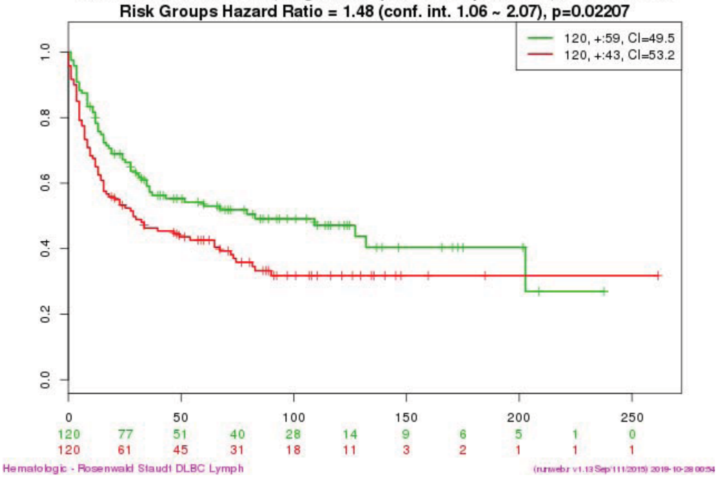

Supplement: Supplementary file 1 [file DataSheet1.ZIP › SI-figure/Figure S21.pdf]
