# Supplementary material for: Interpreting Functional Impact of Genetic Variations by Network QTL for Genotype–Phenotype Association Study
Source: Front Cell Dev Biol. 2022 Jan 26;9:720321. doi: 10.3389/fcell.2021.720321 (PMC8826544; doi:10.3389/fcell.2021.720321)

CENSORED:Survival_Months
7|CXCR6|CDC25C|TGFBR2|CXCL5|ADRA2A|UBA7|XRCC1|CHUK|CCR4|CDC2|CDK2|CDK9|
Concordance Index = 64.25, Log-Rank Equal Curves p=2.07e-06, R^2=0.142/0.997
Risk Groups Hazard Ratio = 2.25 (conf. int. 1.59 ~ 3.19), p=4.858e-06

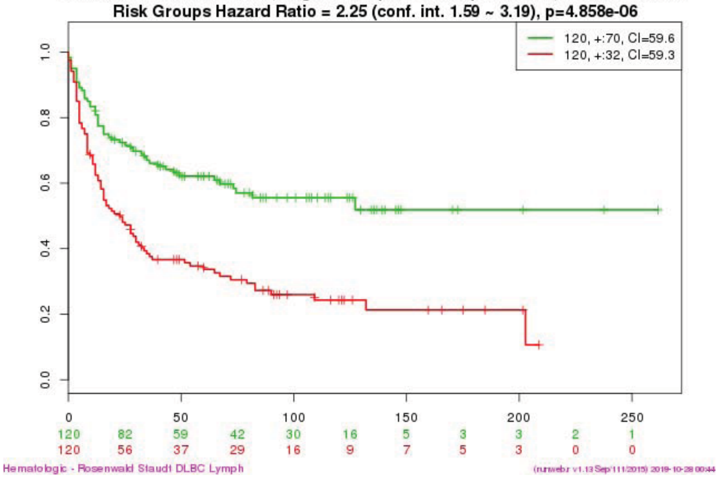

CENSORED:Survival_Months
'NF|UBA7|PIK3CG|IL24|BTK|CD79B|ZNF294|ZFP36L1|MMP14|TKT|PTPN6|CXCL1|MMP17|GI
Concordance Index = 65.52, Log-Rank Equal Curves p=3.039e-06, R^2=0.12/0.997
Risk Groups Hazard Ratio = 2.2 (conf. int. 1.56 ~ 3.11), p=6.649e-06

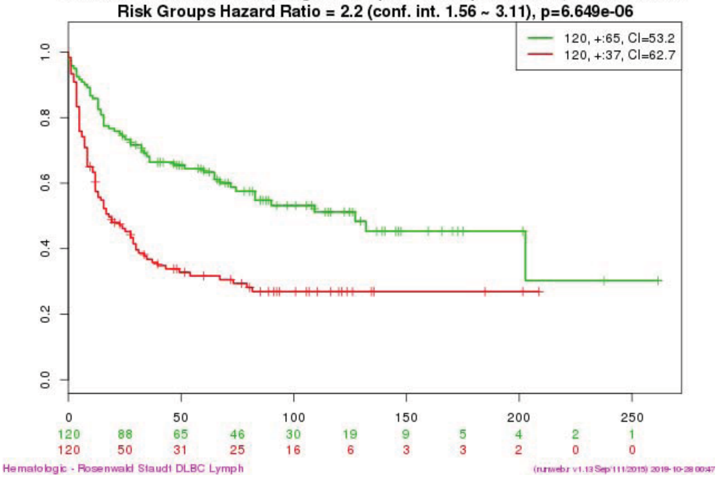

CENSORED:Survival_Months
ADRA2A|CXCL5|IFI30|LHFPL2|GABBR1|CXCL1|MPL
Concordance Index = 60.47, Log-Rank Equal Curves p=0.01746, R^2=0.065/0.997
Risk Groups Hazard Ratio = 1.49 (conf. int. 1.07 ~ 2.09), p=0.0195

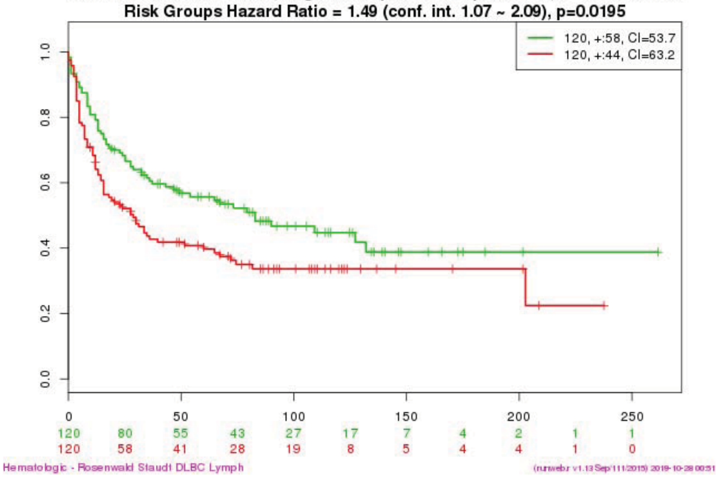

CENSORED:Survival_Months
SELP|MPL|ADRA2A|CDH5|CTR9|ZNF294|GPR4
Concordance Index = 59.45, Log-Rank Equal Curves p=0.004046, R^2=0.044/0.997
Risk Groups Hazard Ratio = 1.63 (conf. int. 1.16 ~ 2.28), p=0.004866

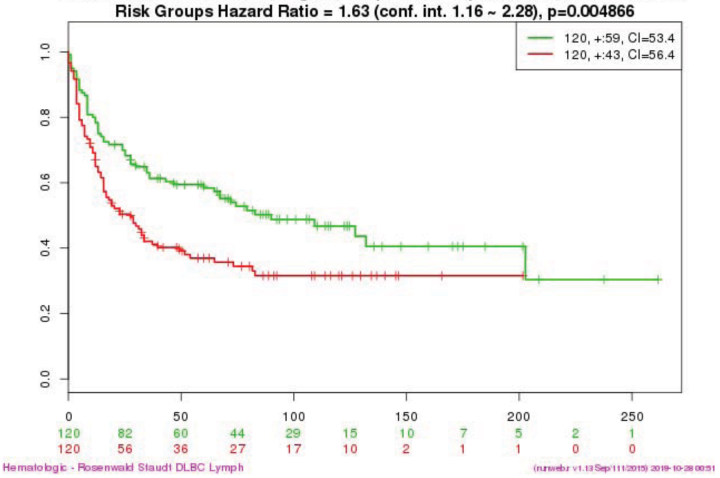

CENSORED:Survival_Months
CYP2B6|CCR4|CYP2J2|MAPK3|PTGS2|CCL11|PAFAH1B1
Concordance Index = 54.93, Log-Rank Equal Curves p=0.836, R^2=0.017/0.997
Risk Groups Hazard Ratio = 1.04 (conf. int. 0.74 ~ 1.45), p=0.8378

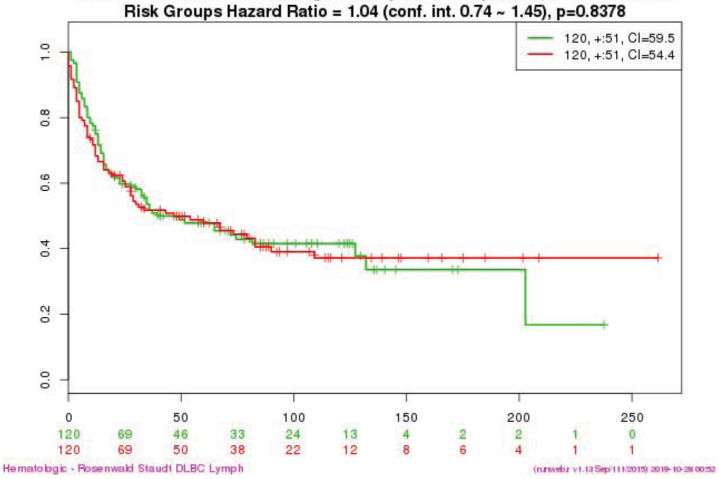

CENSORED:Survival_Months
GNGT2|GPR4|GNB5|EPHA1
Concordance Index = 58.04, Log-Rank Equal Curves p=0.001959, R^2=0.046/0.997
Risk Groups Hazard Ratio = 1.69 (conf. int. 1.2 ~ 2.38), p=0.002462

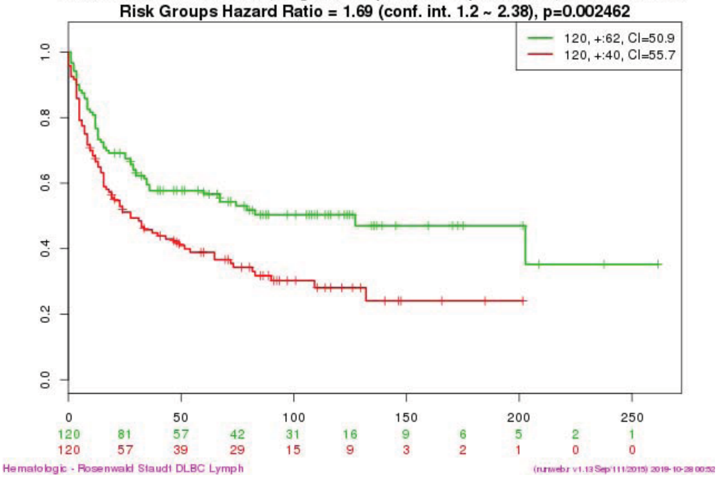

CENSORED:Survival_Months
MPL|HRAS|UBA7|VEGFA|GABBR1
Concordance Index = 55.63, Log-Rank Equal Curves p=0.01905, R^2=0.027/0.997
Risk Groups Hazard Ratio = 1.49 (conf. int. 1.06 ~ 2.08), p=0.02121

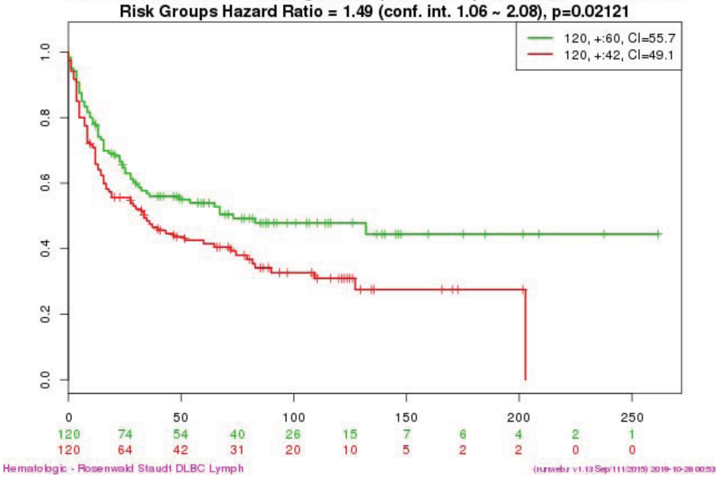

CENSORED:Survival_Months
CD5|GPR4|LYL1
Concordance Index = 56.6, Log-Rank Equal Curves p=0.01986, R^2=0.02/0.997
Risk Groups Hazard Ratio = 1.48 (conf. int. 1.06 ~ 2.07), p=0.02207

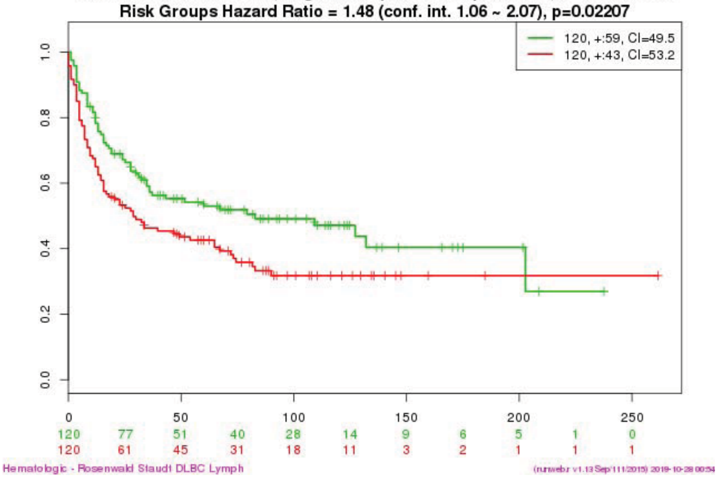

Supplement: Supplementary file 1 [file DataSheet1.ZIP › SI-figure/Figure S22.pdf]
