# Supplementary material for: Interpreting Functional Impact of Genetic Variations by Network QTL for Genotype–Phenotype Association Study
Source: Front Cell Dev Biol. 2022 Jan 26;9:720321. doi: 10.3389/fcell.2021.720321 (PMC8826544; doi:10.3389/fcell.2021.720321)
Supplement: Supplementary file 1 [file DataSheet1.ZIP › SI-figure/Figure S28.pdf]

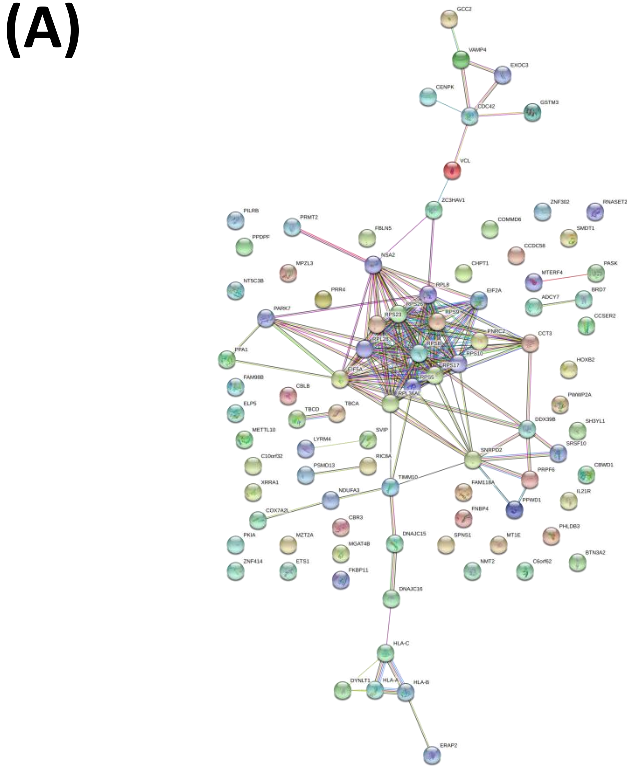

eQTL: gene-network of top-100 genes

Number of nodes:90
Number of edges:147
Average node degree:3.27
Avg. local clustering coefficient:0.427
Expected number of edges:65
PPI enrichment p-value:<1.0e-16

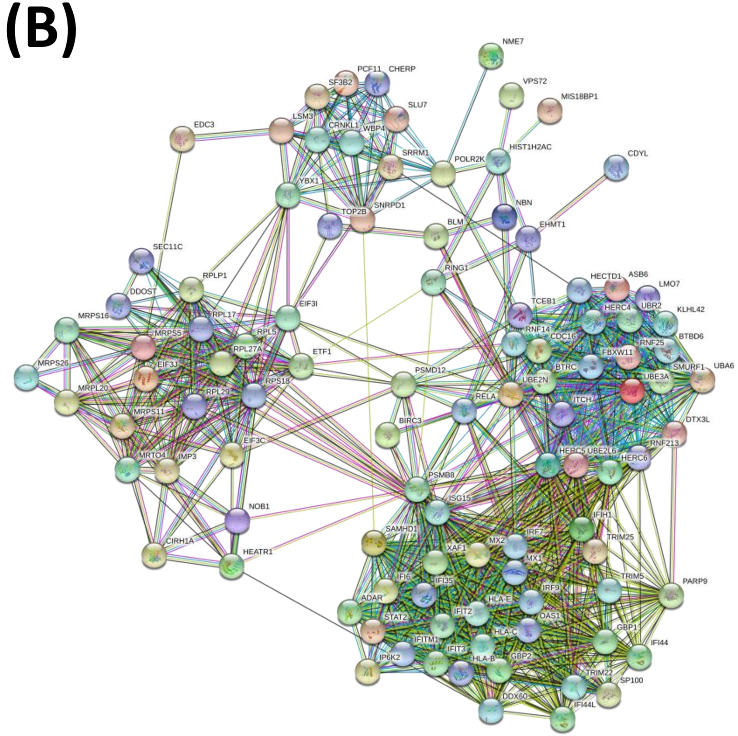

nQTL: gene-network top-100 genes

Number of nodes:103
Number of edges:1024
Average node degree:19.9
Avg. local clustering coefficient:0.746
Expected number of edges:220
PPI enrichment p-value:< 1.0e-16
