# Supplementary material for: Interpreting Functional Impact of Genetic Variations by Network QTL for Genotype–Phenotype Association Study
Source: Front Cell Dev Biol. 2022 Jan 26;9:720321. doi: 10.3389/fcell.2021.720321 (PMC8826544; doi:10.3389/fcell.2021.720321)
Supplement: Supplementary file 1 [file DataSheet1.ZIP › SI-figure/Figure S31.pdf]

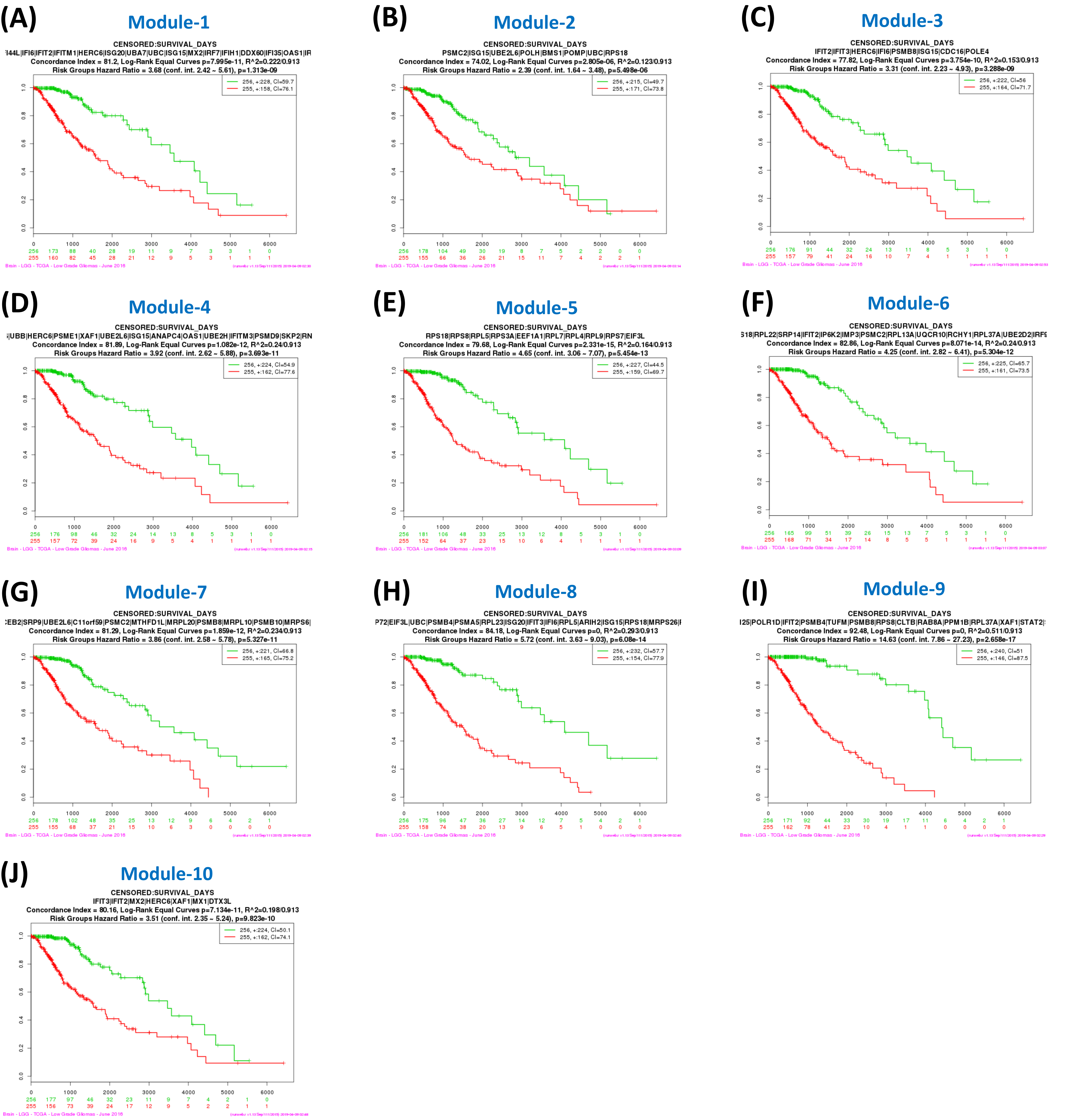

(A)
Module-1
CENSORED:SURVIVAL_DAYS
I44L|IFI6|IFIT2|IFITM1|HERC6|ISG20|UBA7|UBC|ISG15|MX2|IRF7|IFIH1|DDX60|IFI35|OAS1|IR
Concordance Index = 81.2, Log-Rank Equal Curves p=7.995e-11, R^2=0.222/0.913
Risk Groups Hazard Ratio = 3.68 (conf. int. 2.42 ~ 5.61), p=1.313e-09
256, +:228, CI=59.7
255, +:158, CI=76.1
Brain - LGG - TCGA - Low Grade Gliomas - June 2016
(B)
Module-2
CENSORED:SURVIVAL_DAYS
PSMC2|ISG15|UBE2L6|POLH|BMS1|POMP|UBC|RPS18
Concordance Index = 74.02, Log-Rank Equal Curves p=2.805e-06, R^2=0.123/0.913
Risk Groups Hazard Ratio = 2.39 (conf. int. 1.64 ~ 3.48), p=5.498e-06
256, +:215, CI=49.7
255, +:171, CI=73.8
Brain - LGG - TCGA - Low Grade Gliomas - June 2016
(C)
Module-3
CENSORED:SURVIVAL_DAYS
IFIT2|IFIT3|HERC6|IFI6|PSMB8|ISG15|CDC16|POLE4
Concordance Index = 77.82, Log-Rank Equal Curves p=3.754e-10, R^2=0.153/0.913
Risk Groups Hazard Ratio = 3.31 (conf. int. 2.23 ~ 4.93), p=3.288e-09
256, +:222, CI=56
255, +:164, CI=71.7
Brain - LGG - TCGA - Low Grade Gliomas - June 2016
(D)
Module-4
CENSORED:SURVIVAL_DAYS
UBB|HERC6|PSME1|XAF1|UBE2L6|ISG15|ANAPC4|OAS1|UBE2H|IFITM3|PSMD9|SKP2|RN
Concordance Index = 81.89, Log-Rank Equal Curves p=1.082e-12, R^2=0.24/0.913
Risk Groups Hazard Ratio = 3.92 (conf. int. 2.62 ~ 5.88), p=3.693e-11
256, +:224, CI=54.9
255, +:162, CI=77.6
Brain - LGG - TCGA - Low Grade Gliomas - June 2016
(E)
Module-5
CENSORED:SURVIVAL_DAYS
RPS18|RPS8|RPL5|RPS3A|EEF1A1|RPL7|RPL4|RPL9|RPS7|EIF3L
Concordance Index = 79.68, Log-Rank Equal Curves p=2.331e-15, R^2=0.164/0.913
Risk Groups Hazard Ratio = 4.65 (conf. int. 3.06 ~ 7.07), p=5.454e-13
256, +:227, CI=44.5
255, +:159, CI=69.7
Brain - LGG - TCGA - Low Grade Gliomas - June 2016
(F)
Module-6
CENSORED:SURVIVAL_DAYS
S18|RPL22|SRP14|IFIT2|IP6K2|IMP3|PSMC2|RPL13A|UQCR10|RCHY1|RPL37A|UBE2D2|IRF
Concordance Index = 82.86, Log-Rank Equal Curves p=8.071e-14, R^2=0.24/0.913
Risk Groups Hazard Ratio = 4.25 (conf. int. 2.82 ~ 6.41), p=5.304e-12
256, +:225, CI=65.7
255, +:161, CI=73.5
Brain - LGG - TCGA - Low Grade Gliomas - June 2016
(G)
Module-7
CENSORED:SURVIVAL_DAYS
CEB2|SRP9|UBE2L6|C11orf59|PSMC2|MTHFD1L|MRPL20|PSMB8|MRPL10|PSMB10|MRPS6|
Concordance Index = 81.29, Log-Rank Equal Curves p=1.859e-12, R^2=0.234/0.913
Risk Groups Hazard Ratio = 3.86 (conf. int. 2.58 ~ 5.78), p=5.327e-11
256, +:221, CI=66.8
255, +:165, CI=75.2
Brain - LGG - TCGA - Low Grade Gliomas - June 2016
(H)
Module-8
CENSORED:SURVIVAL_DAYS
P72|EIF3L|UBC|PSMB4|PSMA5|RPL23|ISG20|IFIT3|IFI6|RPL5|ARIH2|ISG15|RPS18|MRPS26|
Concordance Index = 84.18, Log-Rank Equal Curves p=0, R^2=0.293/0.913
Risk Groups Hazard Ratio = 5.72 (conf. int. 3.63 ~ 9.03), p=6.08e-14
256, +:232, CI=57.7
255, +:154, CI=77.9
Brain - LGG - TCGA - Low Grade Gliomas - June 2016
(I)
Module-9
CENSORED:SURVIVAL_DAYS
I25|POLR1D|IFIT2|PSMB4|TUFM|PSMB8|RPS8|CLTB|RAB8A|PPM1B|RPL37A|XAF1|STAT2|
Concordance Index = 92.48, Log-Rank Equal Curves p=0, R^2=0.511/0.913
Risk Groups Hazard Ratio = 14.63 (conf. int. 7.86 ~ 27.23), p=2.658e-17
256, +:240, CI=51
255, +:146, CI=87.5
Brain - LGG - TCGA - Low Grade Gliomas - June 2016
(J)
Module-10
CENSORED:SURVIVAL_DAYS
IFIT3|IFIT2|MX2|HERC6|XAF1|MX1|DTX3L
Concordance Index = 80.16, Log-Rank Equal Curves p=7.134e-11, R^2=0.198/0.913
Risk Groups Hazard Ratio = 3.51 (conf. int. 2.35 ~ 5.24), p=9.823e-10
256, +:224, CI=50.1
255, +:162, CI=74.1
Brain - LGG - TCGA - Low Grade Gliomas - June 2016
