# Supplementary material for: Interpreting Functional Impact of Genetic Variations by Network QTL for Genotype–Phenotype Association Study
Source: Front Cell Dev Biol. 2022 Jan 26;9:720321. doi: 10.3389/fcell.2021.720321 (PMC8826544; doi:10.3389/fcell.2021.720321)
Supplement: Supplementary file 1 [file DataSheet1.ZIP › SI-figure/Figure S34.pdf]

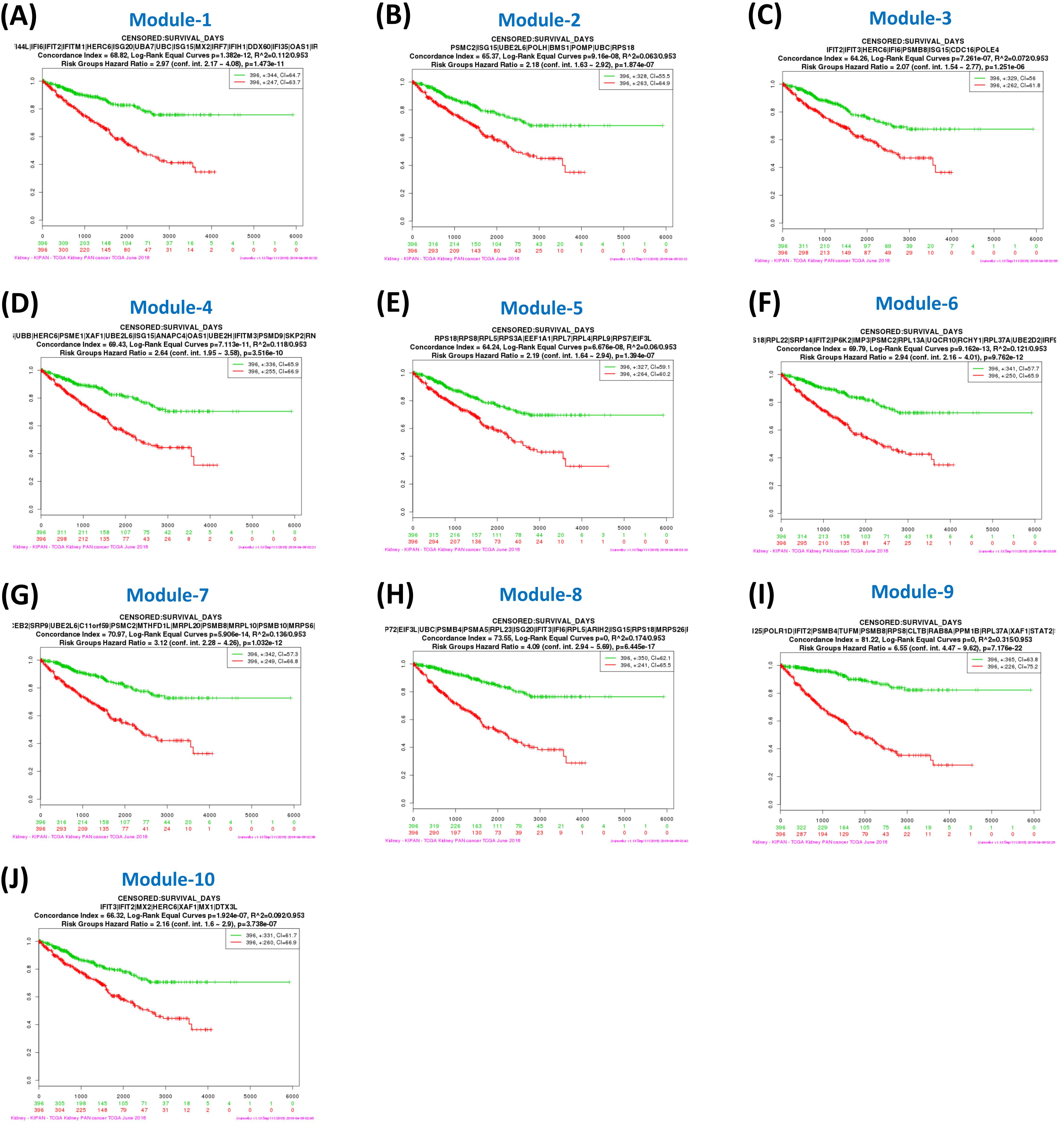

(A)
Module-1
CENSORED:SURVIVAL_DAYS
I44L|IFI6|IFIT2|IFITM1|HERC6|ISG20|UBA7|UBC|ISG15|MX2|IRF7|IFIH1|DDX60|IFI35|OAS1|IR
Concordance Index = 68.82, Log-Rank Equal Curves p=1.382e-12, R^2=0.112/0.953
Risk Groups Hazard Ratio = 2.97 (conf. int. 2.17 ~ 4.08), p=1.473e-11
396, +:344, CI=64.7
396, +:247, CI=63.7
Kidney - KIPAN - TCGA Kidney PANcancer TCGA June 2016
(B)
Module-2
CENSORED:SURVIVAL_DAYS
PSMC2|ISG15|UBE2L6|POLH|BMS1|POMP|UBC|RPS18
Concordance Index = 65.37, Log-Rank Equal Curves p=9.16e-08, R^2=0.063/0.953
Risk Groups Hazard Ratio = 2.18 (conf. int. 1.63 ~ 2.92), p=1.874e-07
396, +:328, CI=55.5
396, +:263, CI=64.9
Kidney - KIPAN - TCGA Kidney PANcancer TCGA June 2016
(C)
Module-3
CENSORED:SURVIVAL_DAYS
IFIT2|IFIT3|HERC6|IFI6|PSMB8|ISG15|CDC16|POLE4
Concordance Index = 64.26, Log-Rank Equal Curves p=7.261e-07, R^2=0.072/0.953
Risk Groups Hazard Ratio = 2.07 (conf. int. 1.54 ~ 2.77), p=1.251e-06
396, +:329, CI=56
396, +:262, CI=61.8
Kidney - KIPAN - TCGA Kidney PANcancer TCGA June 2016
(D)
Module-4
CENSORED:SURVIVAL_DAYS
UBB|HERC6|PSME1|XAF1|UBE2L6|ISG15|ANAPC4|OAS1|UBE2H|IFITM3|PSMD9|SKP2|RN
Concordance Index = 69.43, Log-Rank Equal Curves p=7.113e-11, R^2=0.118/0.953
Risk Groups Hazard Ratio = 2.64 (conf. int. 1.95 ~ 3.58), p=3.516e-10
396, +:336, CI=65.9
396, +:255, CI=66.9
Kidney - KIPAN - TCGA Kidney PANcancer TCGA June 2016
(E)
Module-5
CENSORED:SURVIVAL_DAYS
RPS18|RPS8|RPL5|RPS3A|EEF1A1|RPL7|RPL4|RPL9|RPS7|EIF3L
Concordance Index = 64.24, Log-Rank Equal Curves p=6.676e-08, R^2=0.06/0.953
Risk Groups Hazard Ratio = 2.19 (conf. int. 1.64 ~ 2.94), p=1.394e-07
396, +:327, CI=59.1
396, +:264, CI=60.2
Kidney - KIPAN - TCGA Kidney PANcancer TCGA June 2016
(F)
Module-6
CENSORED:SURVIVAL_DAYS
S18|RPL22|SRP14|IFIT2|IP6K2|IMP3|PSMC2|RPL13A|UQCR10|RCHY1|RPL37A|UBE2D2|IRF
Concordance Index = 69.79, Log-Rank Equal Curves p=9.162e-13, R^2=0.121/0.953
Risk Groups Hazard Ratio = 2.94 (conf. int. 2.16 ~ 4.01), p=9.762e-12
396, +:341, CI=57.7
396, +:250, CI=65.9
Kidney - KIPAN - TCGA Kidney PANcancer TCGA June 2016
(G)
Module-7
CENSORED:SURVIVAL_DAYS
EB2|SRP9|UBE2L6|C11orf59|PSMC2|MTHFD1L|MRPL20|PSMB8|MRPL10|PSMB10|MRPS6|
Concordance Index = 70.97, Log-Rank Equal Curves p=5.906e-14, R^2=0.136/0.953
Risk Groups Hazard Ratio = 3.12 (conf. int. 2.28 ~ 4.26), p=1.032e-12
396, +:342, CI=57.3
396, +:249, CI=66.8
Kidney - KIPAN - TCGA Kidney PANcancer TCGA June 2016
(H)
Module-8
CENSORED:SURVIVAL_DAYS
P72|EIF3L|UBC|PSMB4|PSMA5|RPL23|ISG20|IFIT3|IFI6|RPL5|ARIH2|ISG15|RPS18|MRPS26|
Concordance Index = 73.55, Log-Rank Equal Curves p=0, R^2=0.174/0.953
Risk Groups Hazard Ratio = 4.09 (conf. int. 2.94 ~ 5.69), p=6.445e-17
396, +:350, CI=62.1
396, +:241, CI=65.5
Kidney - KIPAN - TCGA Kidney PANcancer TCGA June 2016
(I)
Module-9
CENSORED:SURVIVAL_DAYS
I25|POLR1D|IFIT2|PSMB4|TUFM|PSMB8|RPS8|CLTB|RAB8A|PPM1B|RPL37A|XAF1|STAT2|
Concordance Index = 81.22, Log-Rank Equal Curves p=0, R^2=0.315/0.953
Risk Groups Hazard Ratio = 6.55 (conf. int. 4.47 ~ 9.62), p=7.176e-22
396, +:365, CI=63.8
396, +:226, CI=75.2
Kidney - KIPAN - TCGA Kidney PANcancer TCGA June 2016
(J)
Module-10
CENSORED:SURVIVAL_DAYS
IFIT3|IFIT2|MX2|HERC6|XAF1|MX1|DTX3L
Concordance Index = 66.32, Log-Rank Equal Curves p=1.924e-07, R^2=0.092/0.953
Risk Groups Hazard Ratio = 2.16 (conf. int. 1.6 ~ 2.9), p=3.738e-07
396, +:331, CI=61.7
396, +:260, CI=66.9
Kidney - KIPAN - TCGA Kidney PANcancer TCGA June 2016
