# Supplementary material for: Interpreting Functional Impact of Genetic Variations by Network QTL for Genotype–Phenotype Association Study
Source: Front Cell Dev Biol. 2022 Jan 26;9:720321. doi: 10.3389/fcell.2021.720321 (PMC8826544; doi:10.3389/fcell.2021.720321)
Supplement: Supplementary file 1 [file DataSheet1.ZIP › SI-figure/Figure S36.pdf]

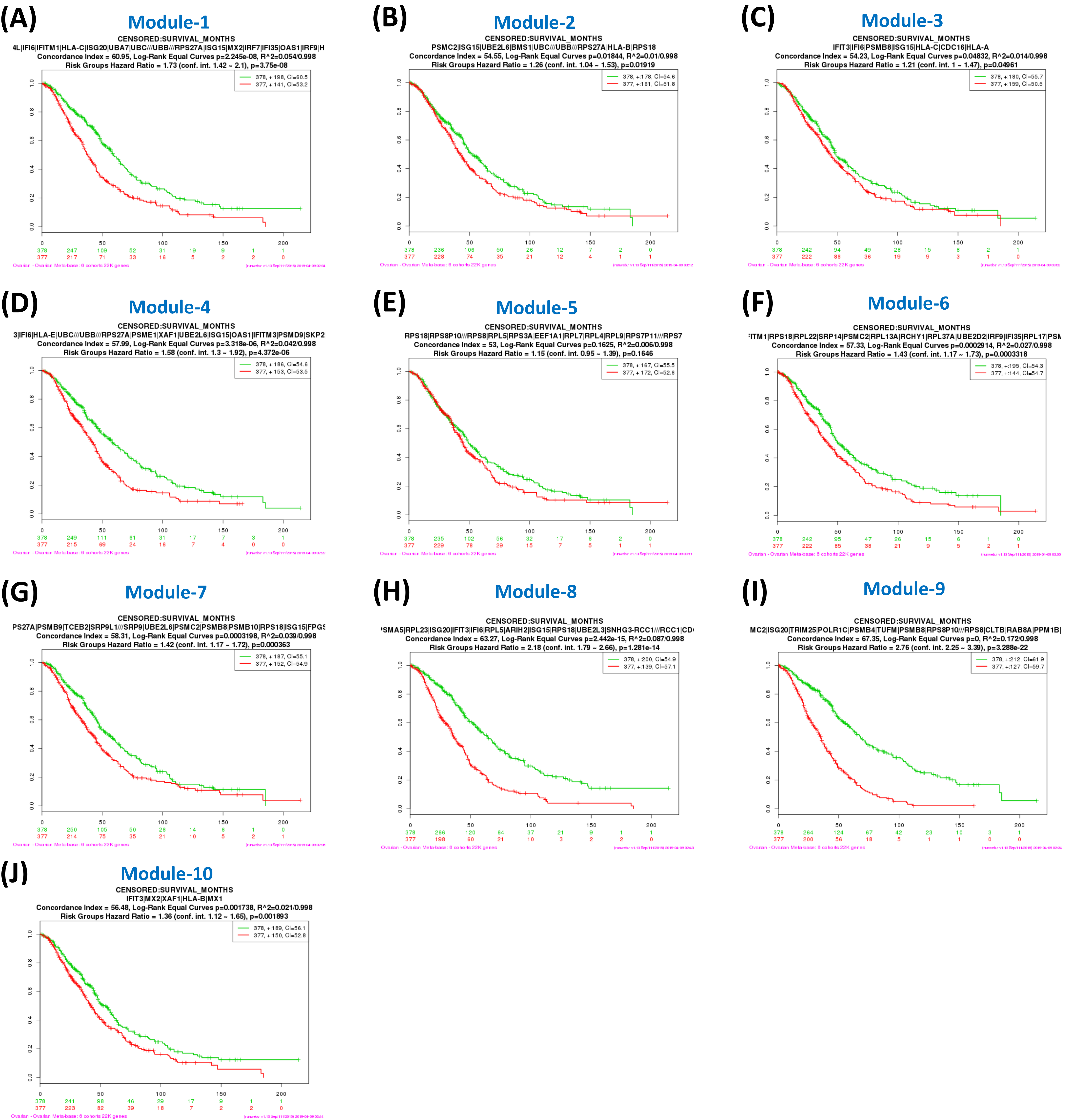
(A) Module-1
CENSORED:SURVIVAL_MONTHS
4L|IFI6|IFITM1|HLA-C|ISG20|UBA7|UBC///UBB///RPS27A|ISG15|MX2|IRF7|IFI35|OAS1|IRF9|H
Concordance Index = 60.95, Log-Rank Equal Curves p=2.245e-08, R^2=0.054/0.998
Risk Groups Hazard Ratio = 1.73 (conf. int. 1.42 ~ 2.1), p=3.75e-08
378, +:198, CI=60.5
377, +:141, CI=53.2
378 247 109 52 31 19 9 1 1
377 217 71 33 16 5 2 2 0
Ovarian - Ovarian Meta-base: 6 cohorts 22K genes
(B) Module-2
CENSORED:SURVIVAL_MONTHS
PSMC2|ISG15|UBE2L6|BMS1|UBC///UBB///RPS27A|HLA-B|RPS18
Concordance Index = 54.55, Log-Rank Equal Curves p=0.01844, R^2=0.01/0.998
Risk Groups Hazard Ratio = 1.26 (conf. int. 1.04 ~ 1.53), p=0.01919
378, +:178, CI=54.6
377, +:161, CI=51.8
378 236 106 50 26 12 7 2 0
377 228 74 35 21 12 4 1 1
Ovarian - Ovarian Meta-base: 6 cohorts 22K genes
(C) Module-3
CENSORED:SURVIVAL_MONTHS
IFIT3|IFI6|PSMB8|ISG15|HLA-C|CDC16|HLA-A
Concordance Index = 54.23, Log-Rank Equal Curves p=0.04832, R^2=0.014/0.998
Risk Groups Hazard Ratio = 1.21 (conf. int. 1 ~ 1.47), p=0.04961
378, +:180, CI=55.7
377, +:159, CI=50.5
378 242 94 49 28 15 8 2 1
377 222 86 36 19 9 3 1 0
Ovarian - Ovarian Meta-base: 6 cohorts 22K genes
(D) Module-4
CENSORED:SURVIVAL_MONTHS
3|IFI6|HLA-E|UBC///UBB///RPS27A|PSME1|XAF1|UBE2L6|ISG15|OAS1|IFITM3|PSMD9|SKP2
Concordance Index = 57.99, Log-Rank Equal Curves p=3.318e-06, R^2=0.042/0.998
Risk Groups Hazard Ratio = 1.58 (conf. int. 1.3 ~ 1.92), p=4.372e-06
378, +:186, CI=54.6
377, +:153, CI=53.5
378 249 111 61 31 17 7 3 1
377 215 69 24 16 7 4 0 0
Ovarian - Ovarian Meta-base: 6 cohorts 22K genes
(E) Module-5
CENSORED:SURVIVAL_MONTHS
RPS18|RPS8P10///RPS8|RPL5|RPS3A|EEF1A1|RPL7|RPL4|RPL9|RPS7P11///RPS7
Concordance Index = 53, Log-Rank Equal Curves p=0.1625, R^2=0.006/0.998
Risk Groups Hazard Ratio = 1.15 (conf. int. 0.95 ~ 1.39), p=0.1646
378, +:167, CI=55.5
377, +:172, CI=52.6
378 235 102 56 32 17 6 2 0
377 229 78 29 15 7 5 1 1
Ovarian - Ovarian Meta-base: 6 cohorts 22K genes
(F) Module-6
CENSORED:SURVIVAL_MONTHS
ITM1|RPS18|RPL22|SRP14|PSMC2|RPL13A|RCHY1|RPL37A|UBE2D2|IRF9|IFI35|RPL17|PSM
Concordance Index = 57.33, Log-Rank Equal Curves p=0.0002914, R^2=0.027/0.998
Risk Groups Hazard Ratio = 1.43 (conf. int. 1.17 ~ 1.73), p=0.0003318
378, +:195, CI=54.3
377, +:144, CI=54.7
378 242 95 47 26 15 6 1 0
377 222 85 38 21 9 5 2 1
Ovarian - Ovarian Meta-base: 6 cohorts 22K genes
(G) Module-7
CENSORED:SURVIVAL_MONTHS
PS27A|PSMB9|TCEB2|SRP9L1///SRP9|UBE2L6|PSMC2|PSMB8|PSMB10|RPS18|ISG15|FPGS
Concordance Index = 58.31, Log-Rank Equal Curves p=0.0003198, R^2=0.039/0.998
Risk Groups Hazard Ratio = 1.42 (conf. int. 1.17 ~ 1.72), p=0.000363
378, +:187, CI=55.1
377, +:152, CI=54.9
378 250 105 50 26 14 6 1 0
377 214 75 35 21 10 5 2 1
Ovarian - Ovarian Meta-base: 6 cohorts 22K genes
(H) Module-8
CENSORED:SURVIVAL_MONTHS
SMA5|RPL23|ISG20|IFIT3|IFI6|RPL5|ARIH2|ISG15|RPS18|UBE2L3|SNHG3-RCC1///RCC1|CD
Concordance Index = 63.27, Log-Rank Equal Curves p=2.442e-15, R^2=0.087/0.998
Risk Groups Hazard Ratio = 2.18 (conf. int. 1.79 ~ 2.66), p=1.281e-14
378, +:200, CI=54.9
377, +:139, CI=57.1
378 266 120 64 37 21 9 1 1
377 198 60 21 10 3 2 2 0
Ovarian - Ovarian Meta-base: 6 cohorts 22K genes
(I) Module-9
CENSORED:SURVIVAL_MONTHS
MC2|ISG20|TRIM25|POLR1C|PSMB4|TUFM|PSMB8|RPS8P10///RPS8|CLTB|RAB8A|PPM1B|
Concordance Index = 67.35, Log-Rank Equal Curves p=0, R^2=0.172/0.998
Risk Groups Hazard Ratio = 2.76 (conf. int. 2.25 ~ 3.39), p=3.288e-22
378, +:212, CI=61.9
377, +:127, CI=59.7
378 264 124 67 42 23 10 3 1
377 200 56 18 5 1 1 0 0
Ovarian - Ovarian Meta-base: 6 cohorts 22K genes
(J) Module-10
CENSORED:SURVIVAL_MONTHS
IFIT3|MX2|XAF1|HLA-B|MX1
Concordance Index = 56.48, Log-Rank Equal Curves p=0.001738, R^2=0.021/0.998
Risk Groups Hazard Ratio = 1.36 (conf. int. 1.12 ~ 1.65), p=0.001893
378, +:189, CI=56.1
377, +:150, CI=52.8
378 241 98 46 29 17 9 1 1
377 223 82 39 18 7 2 2 0
Ovarian - Ovarian Meta-base: 6 cohorts 22K genes
