# Supplementary material for: Interpreting Functional Impact of Genetic Variations by Network QTL for Genotype–Phenotype Association Study
Source: Front Cell Dev Biol. 2022 Jan 26;9:720321. doi: 10.3389/fcell.2021.720321 (PMC8826544; doi:10.3389/fcell.2021.720321)
Supplement: Supplementary file 1 [file DataSheet1.ZIP › SI-figure/Figure S38.pdf]

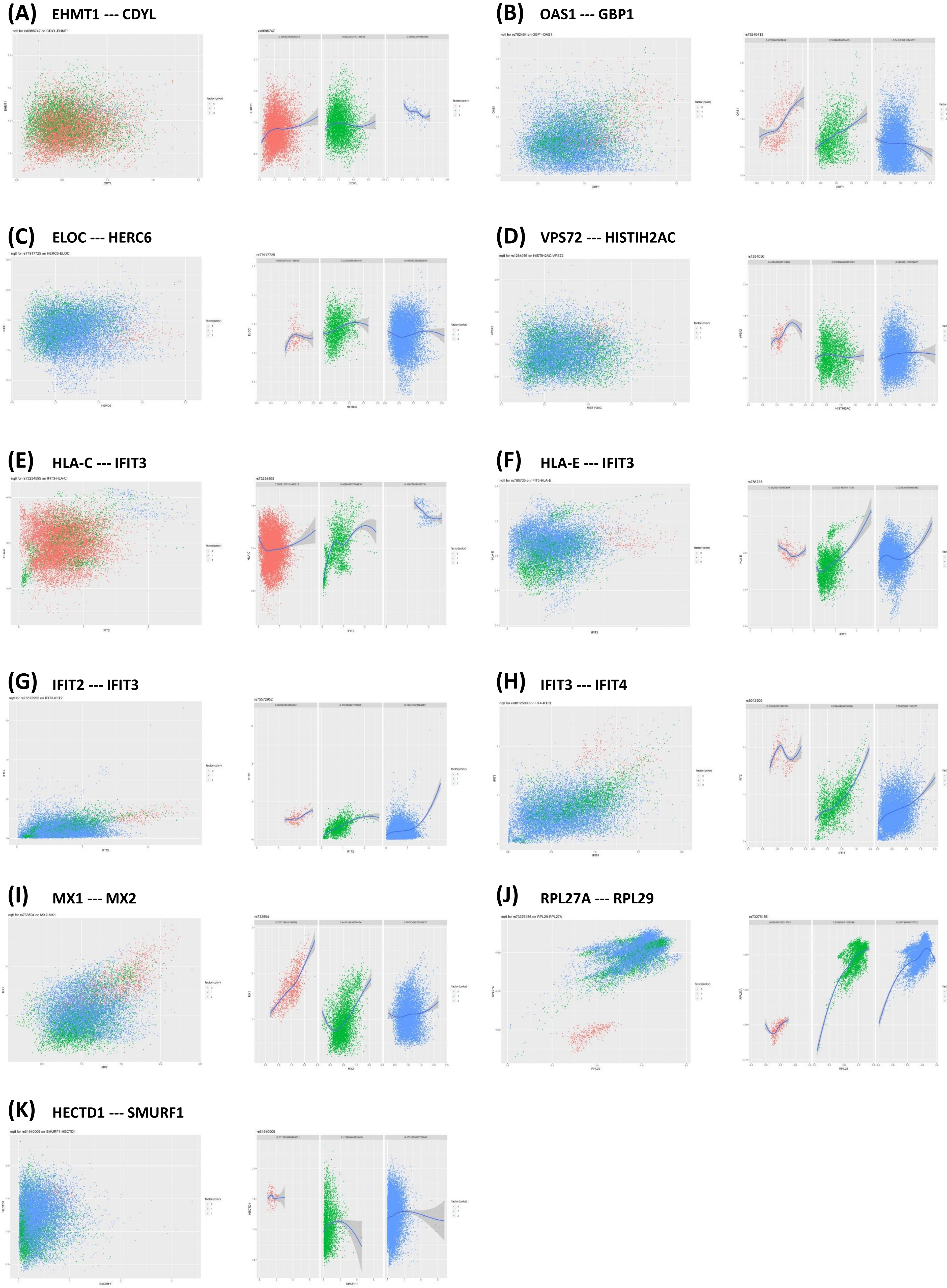
(A) EHMT1 --- CDYL
(B) OAS1 --- GBP1
(C) ELOC --- HERC6
(D) VPS72 --- HISTIH2AC
(E) HLA-C --- IFIT3
(F) HLA-E --- IFIT3
(G) IFIT2 --- IFIT3
(H) IFIT3 --- IFIT4
(I) MX1 --- MX2
(J) RPL27A --- RPL29
(K) HECTD1 --- SMURF1
