# Supplementary material for: Interpreting Functional Impact of Genetic Variations by Network QTL for Genotype–Phenotype Association Study
Source: Front Cell Dev Biol. 2022 Jan 26;9:720321. doi: 10.3389/fcell.2021.720321 (PMC8826544; doi:10.3389/fcell.2021.720321)
Supplement: Supplementary file 1 [file DataSheet1.ZIP › SI-figure/Figure S39.pdf]

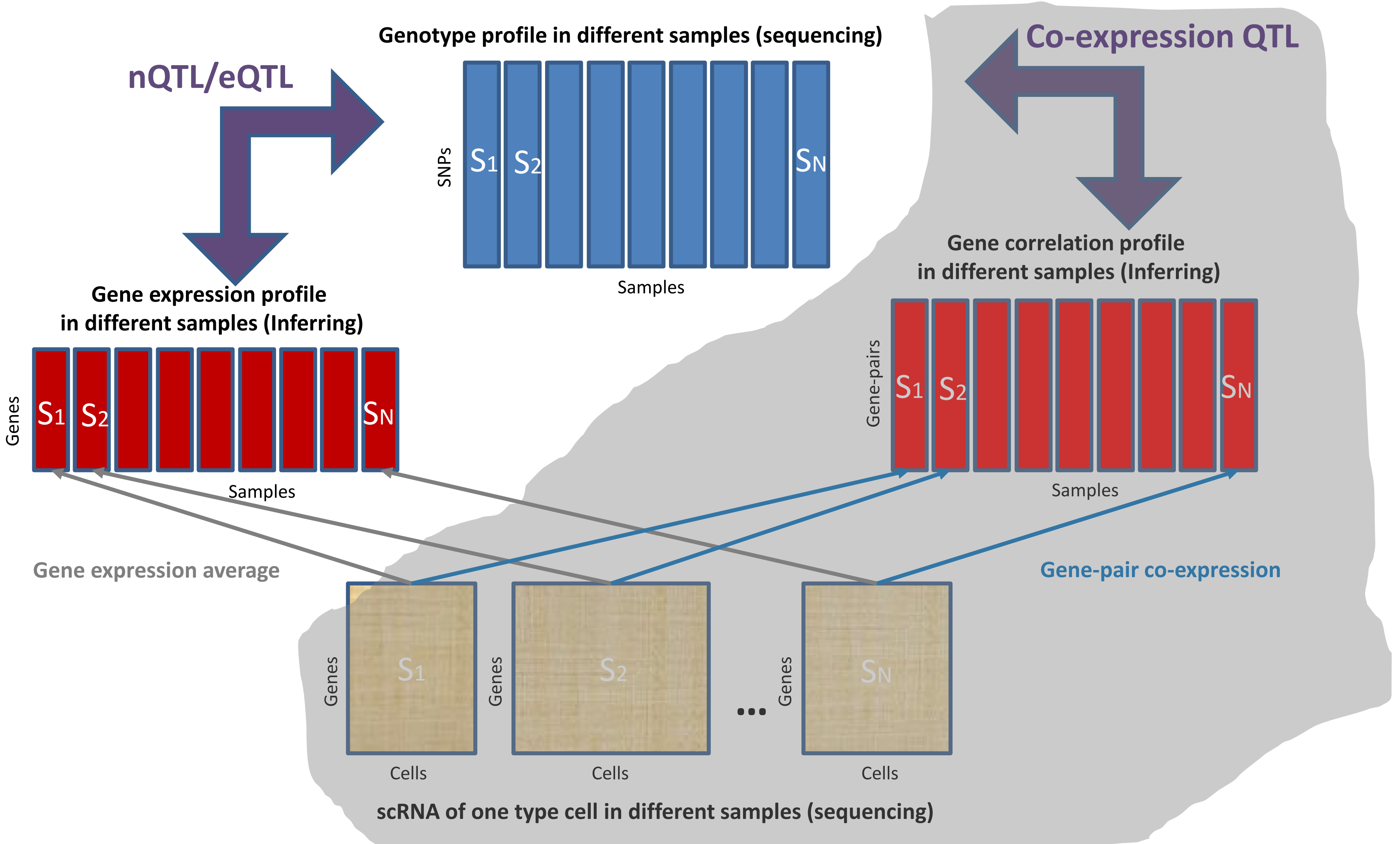
Genotype profile in different samples (sequencing)
nQTL/eQTL
Co-expression QTL
SNPs
S1
S2
SN
Samples
Gene expression profile
in different samples (Inferring)
Genes
S1
S2
SN
Samples
Gene correlation profile
in different samples (Inferring)
Gene-pairs
S1
S2
SN
Samples
Gene expression average
Gene-pair co-expression
Genes
S1
Cells
Genes
S2
Cells
...
Genes
SN
Cells
scRNA of one type cell in different samples (sequencing)
