# Supplementary material for: Interpreting Functional Impact of Genetic Variations by Network QTL for Genotype–Phenotype Association Study
Source: Front Cell Dev Biol. 2022 Jan 26;9:720321. doi: 10.3389/fcell.2021.720321 (PMC8826544; doi:10.3389/fcell.2021.720321)
Supplement: Supplementary file 1 [file DataSheet1.ZIP › SI-figure/Figure S7.pdf]

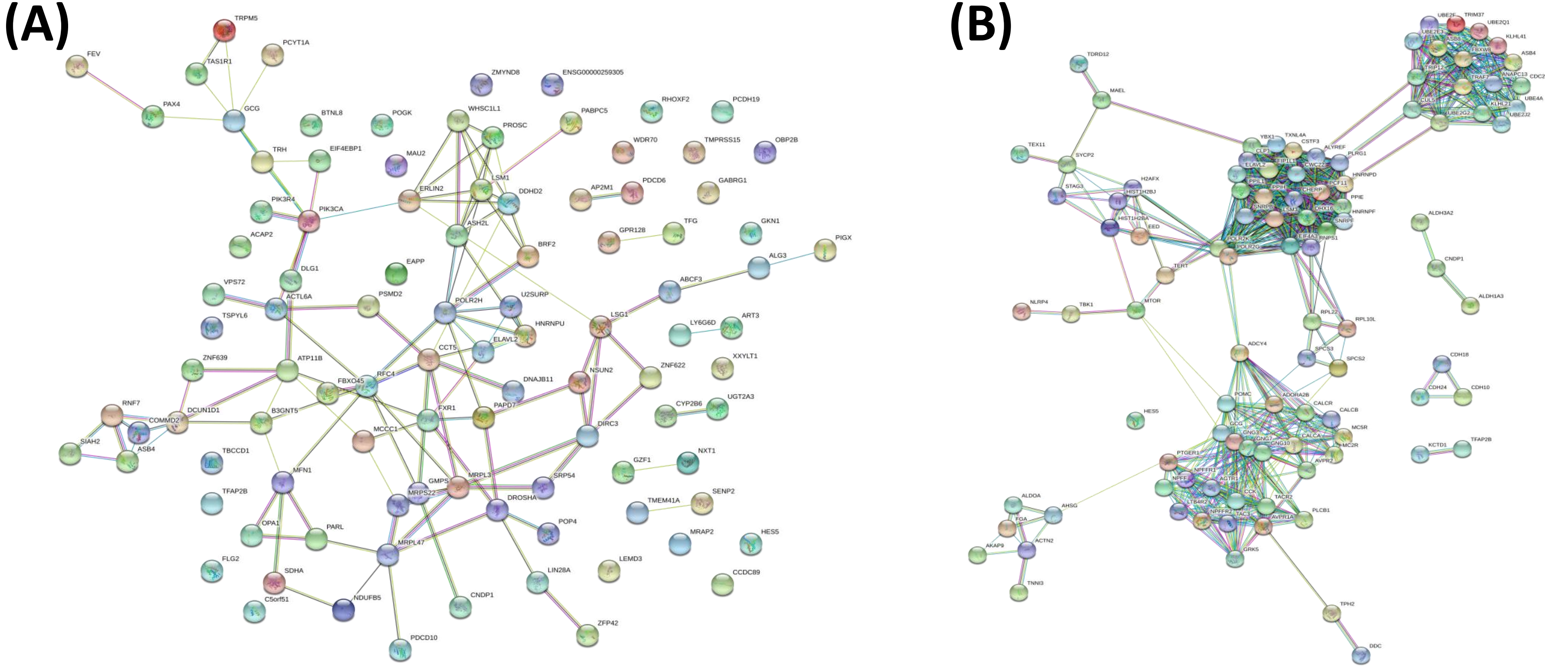

eQTL: gene-network of top-100 genes

Number of nodes:100

Number of edges:118

Average node degree:2.36

Avg. local clustering coefficient:0.461

Expected number of edges:62

PPI enrichment p-value:1.45e-10

nQTL: gene-network top-100 genes

Number of nodes:100

Number of edges:687

Average node degree:13.7

Avg. local clustering coefficient:0.829

Expected number of edges:218

PPI enrichment p-value:< 1.0e-16
