# Supplementary figures and images for: Interpreting Functional Impact of Genetic Variations by Network QTL for Genotype–Phenotype Association Study
Source: Front Cell Dev Biol. 2022 Jan 26;9:720321. doi: 10.3389/fcell.2021.720321 (PMC8826544; doi:10.3389/fcell.2021.720321)

(A)

Module-1

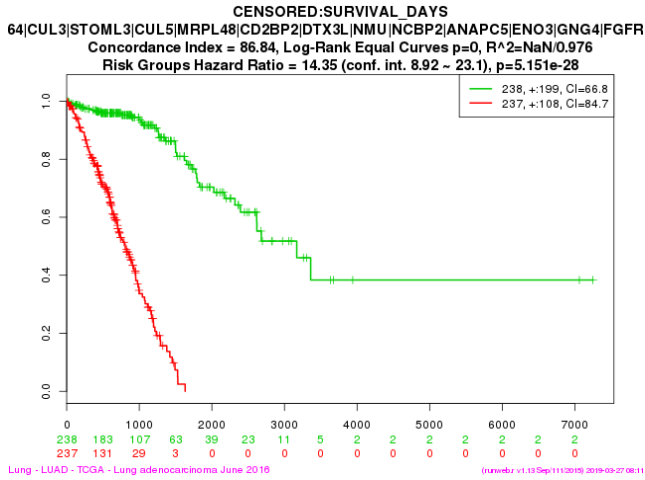

(B)

Module-2

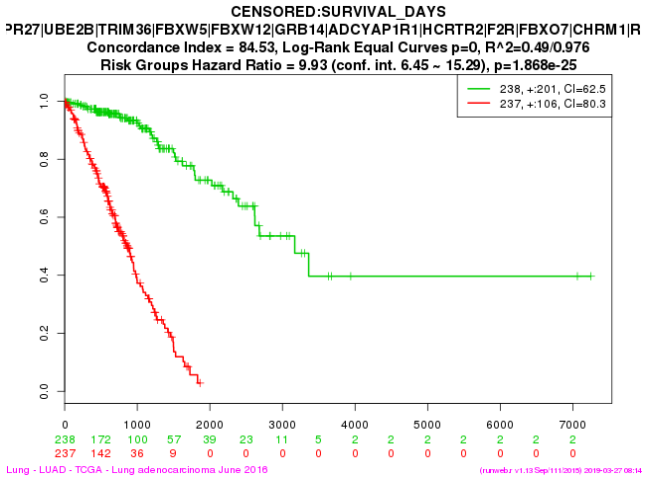

(C)

Module-3

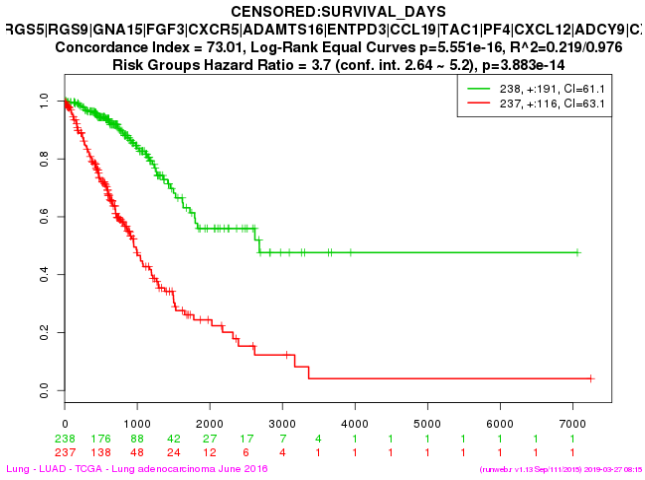

(D)

Module-4

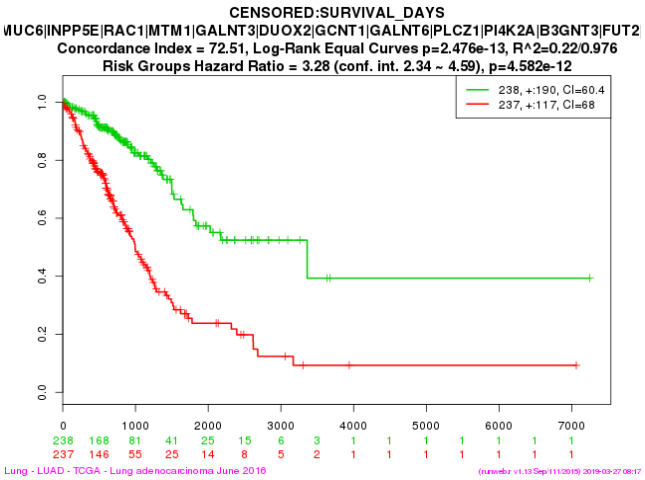

(E)

Module-5

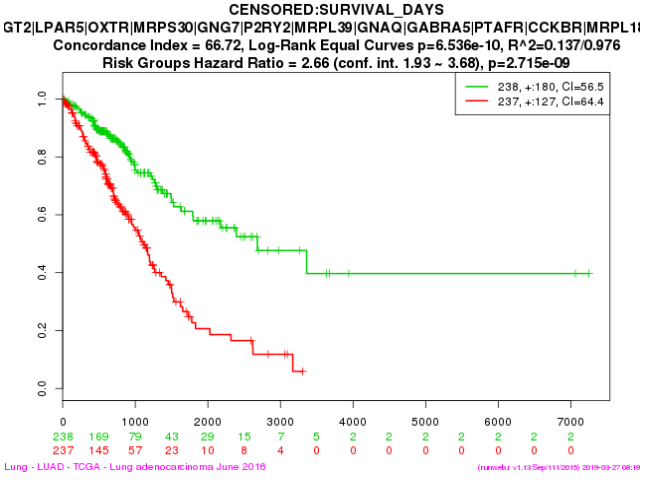

(F)

Module-6

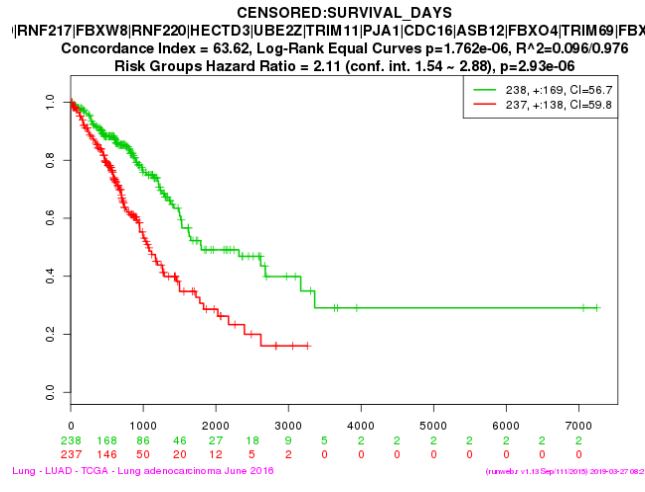

(G)

Module-7

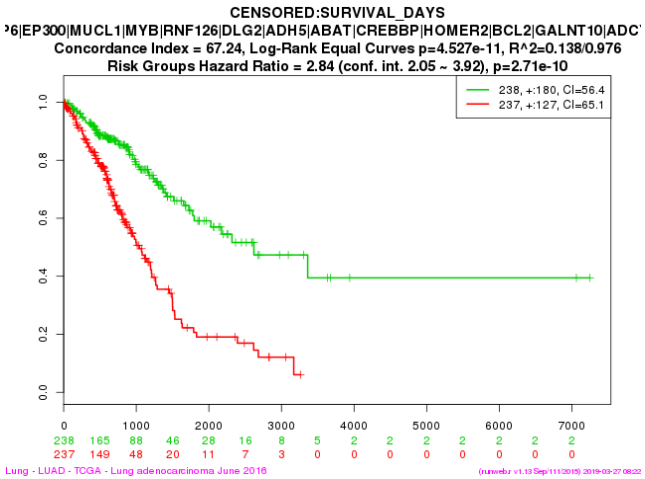

(H)

Module-8

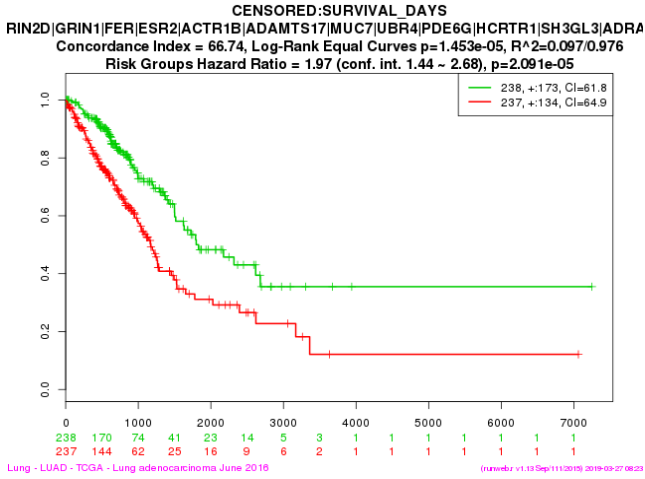

(I)

Module-9

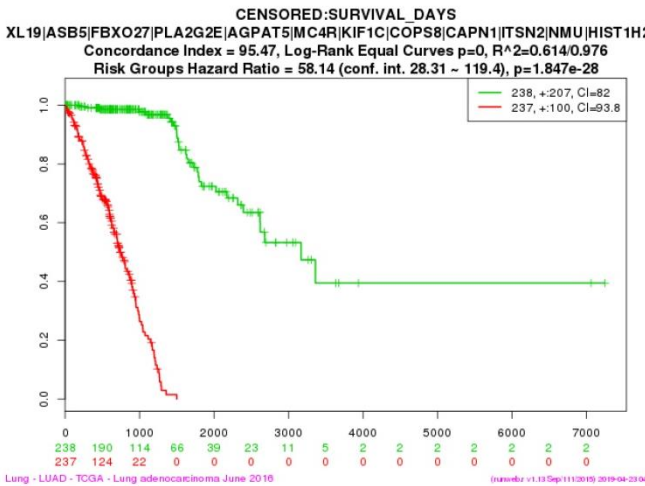

(J)

Module-10

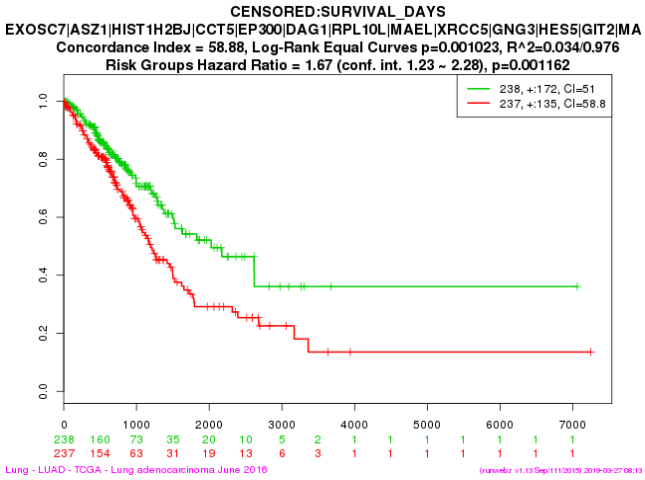

Supplement: Supplementary file 1 [file DataSheet1.ZIP › SI-figure/Figure S10.pdf]

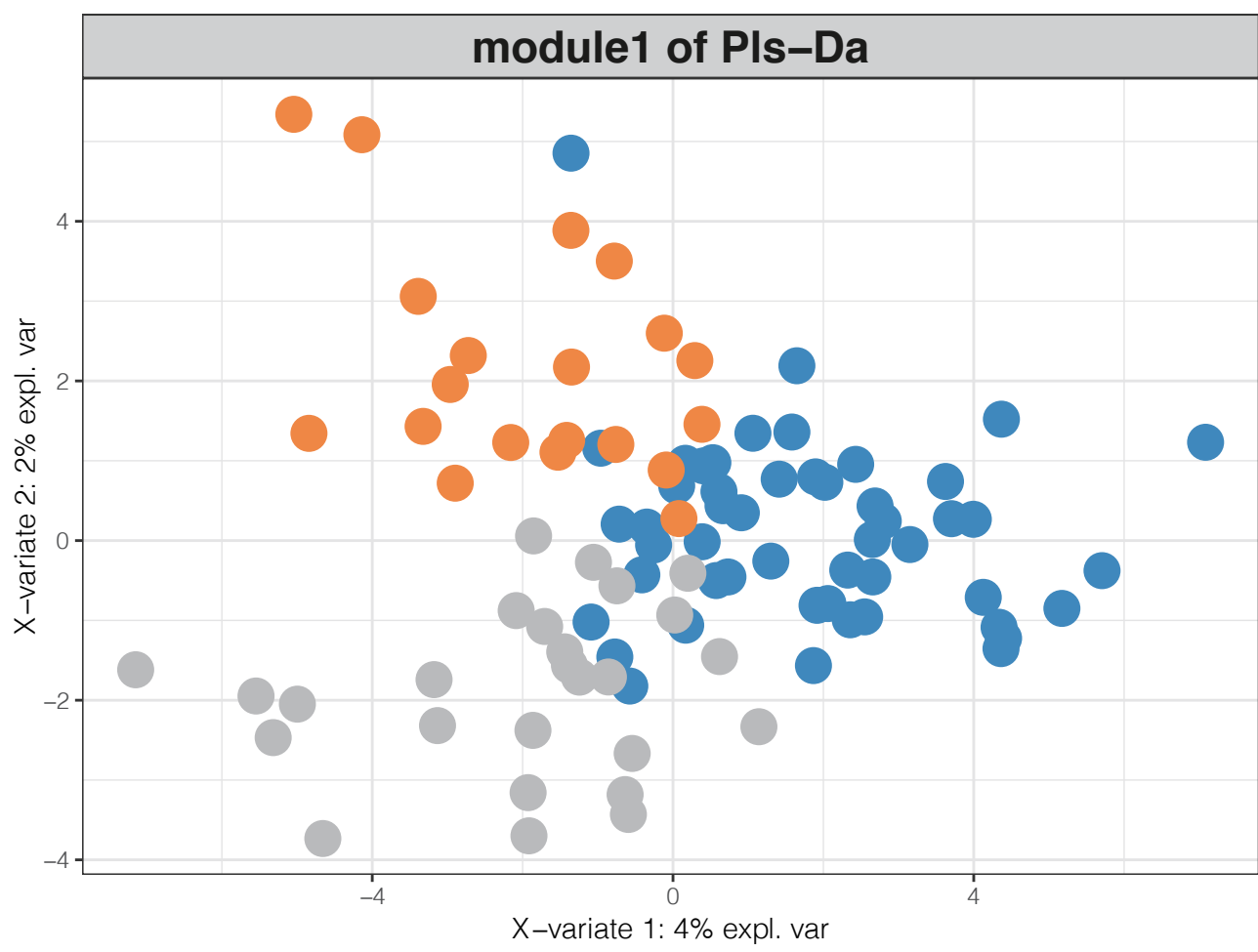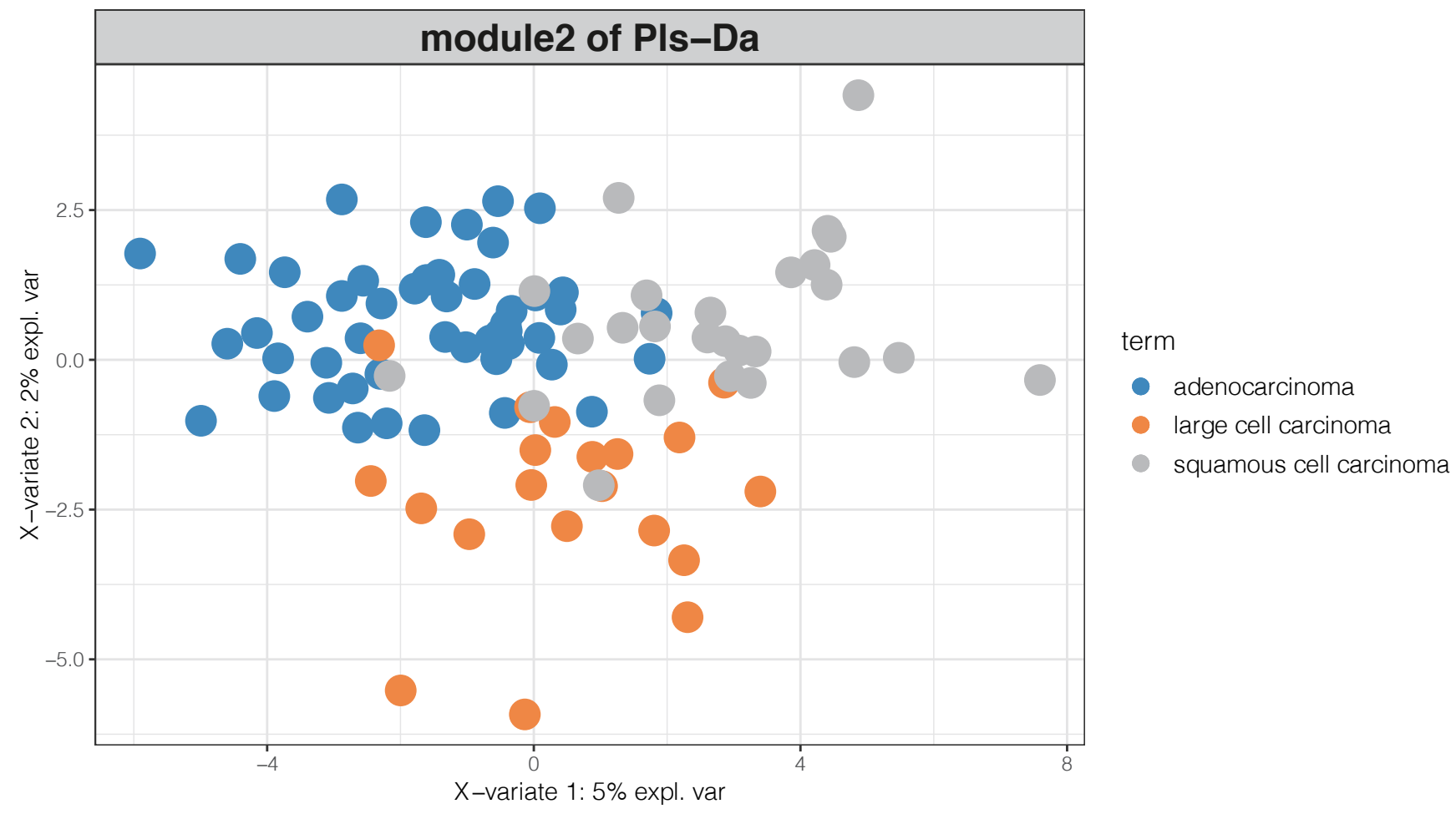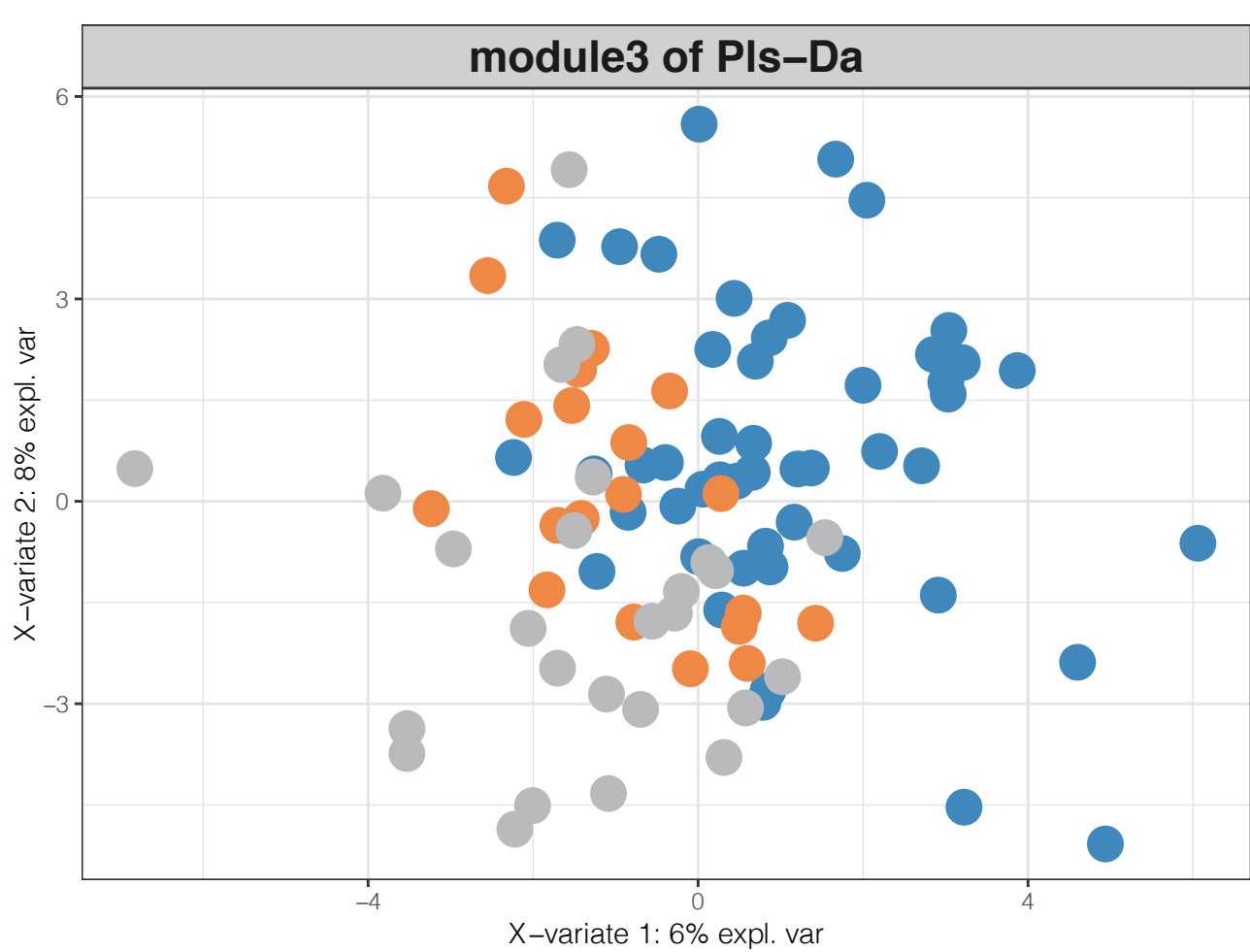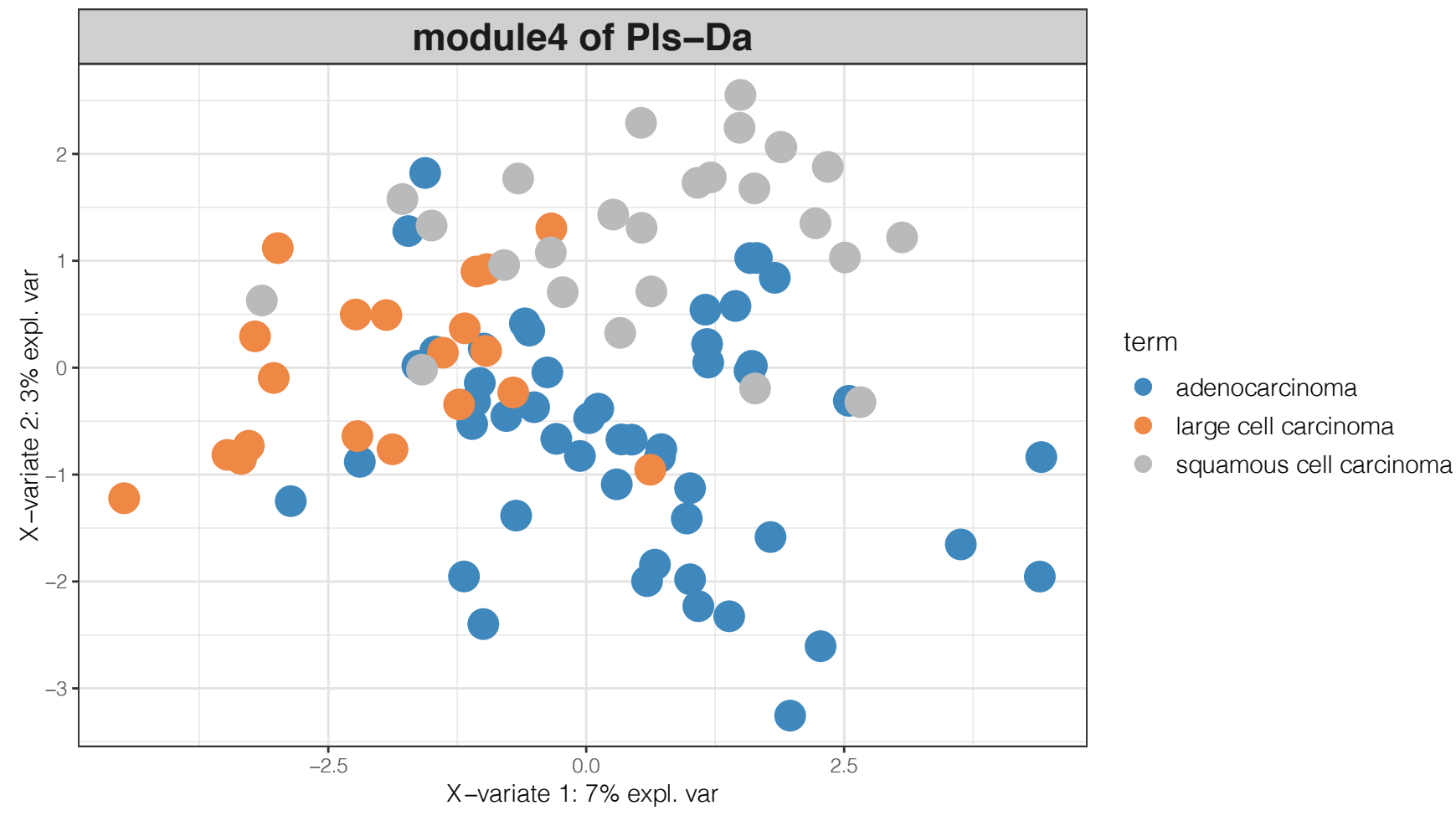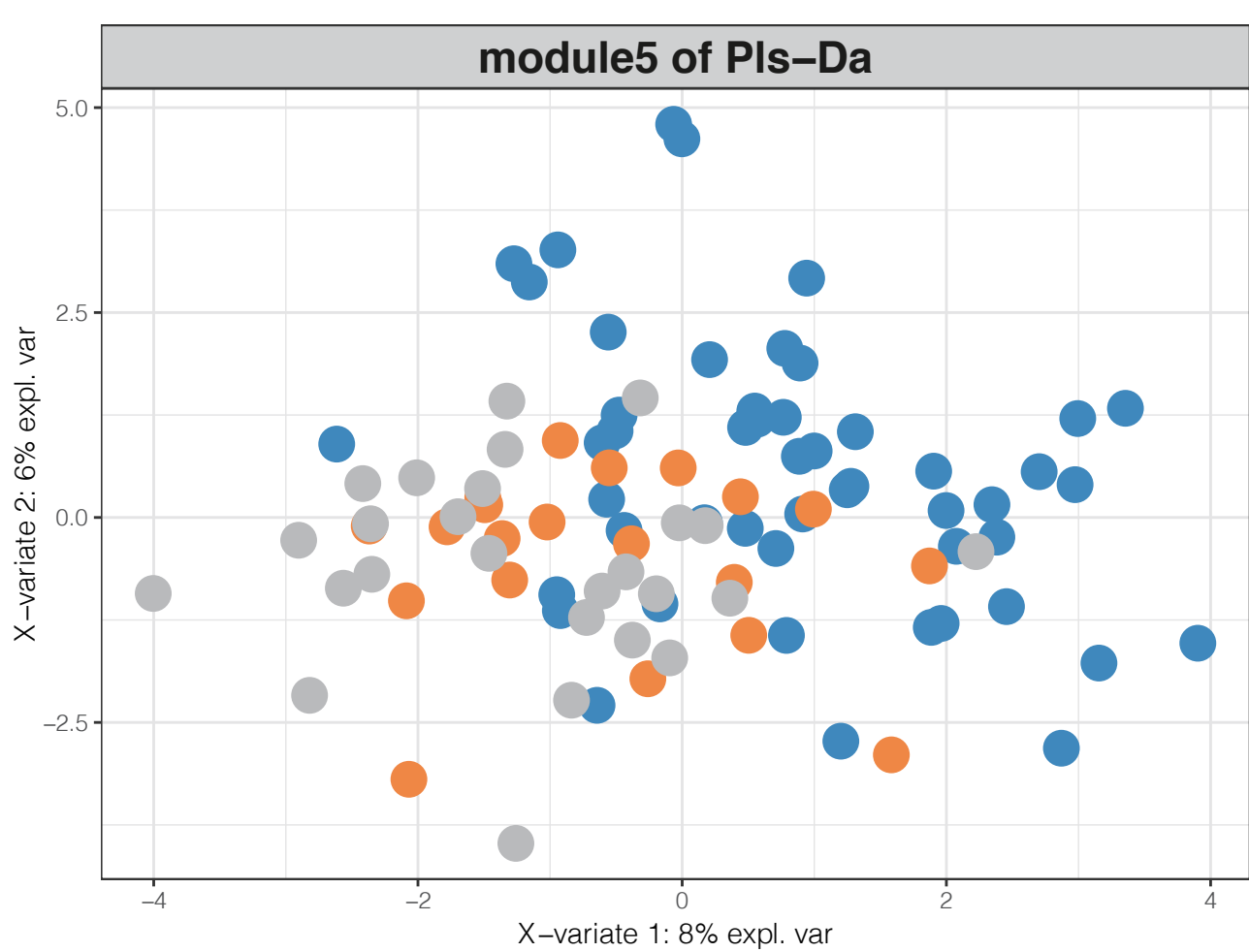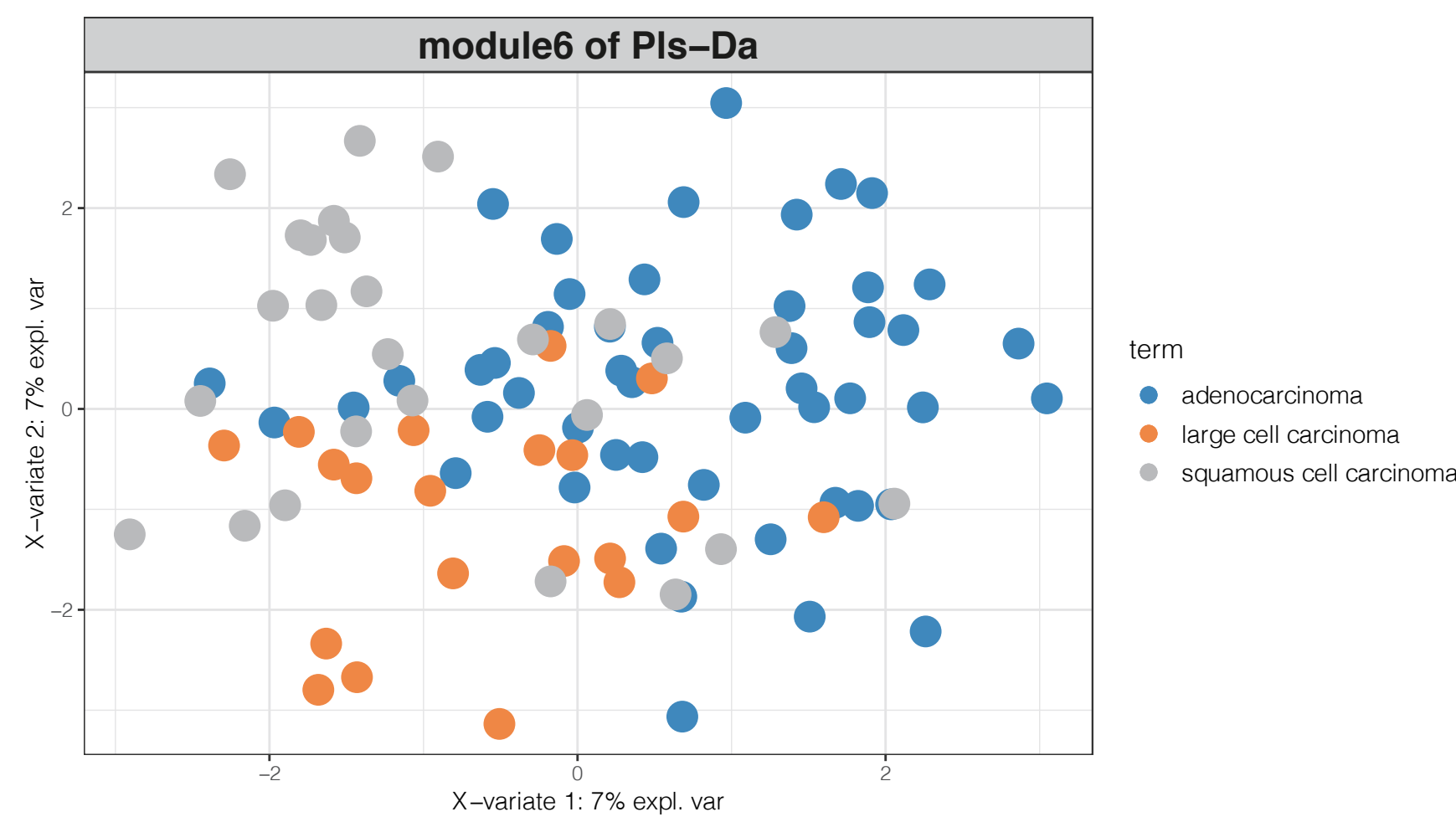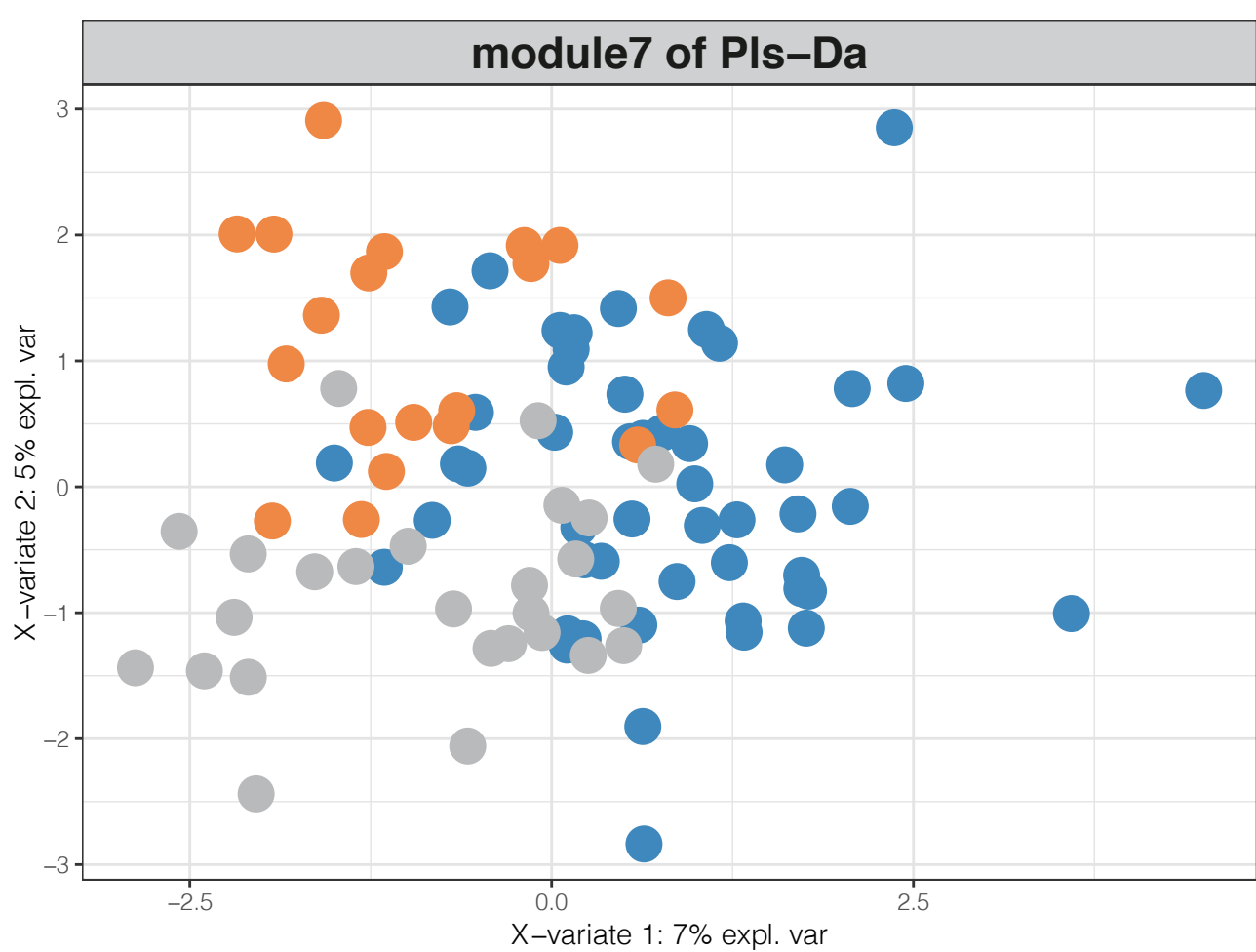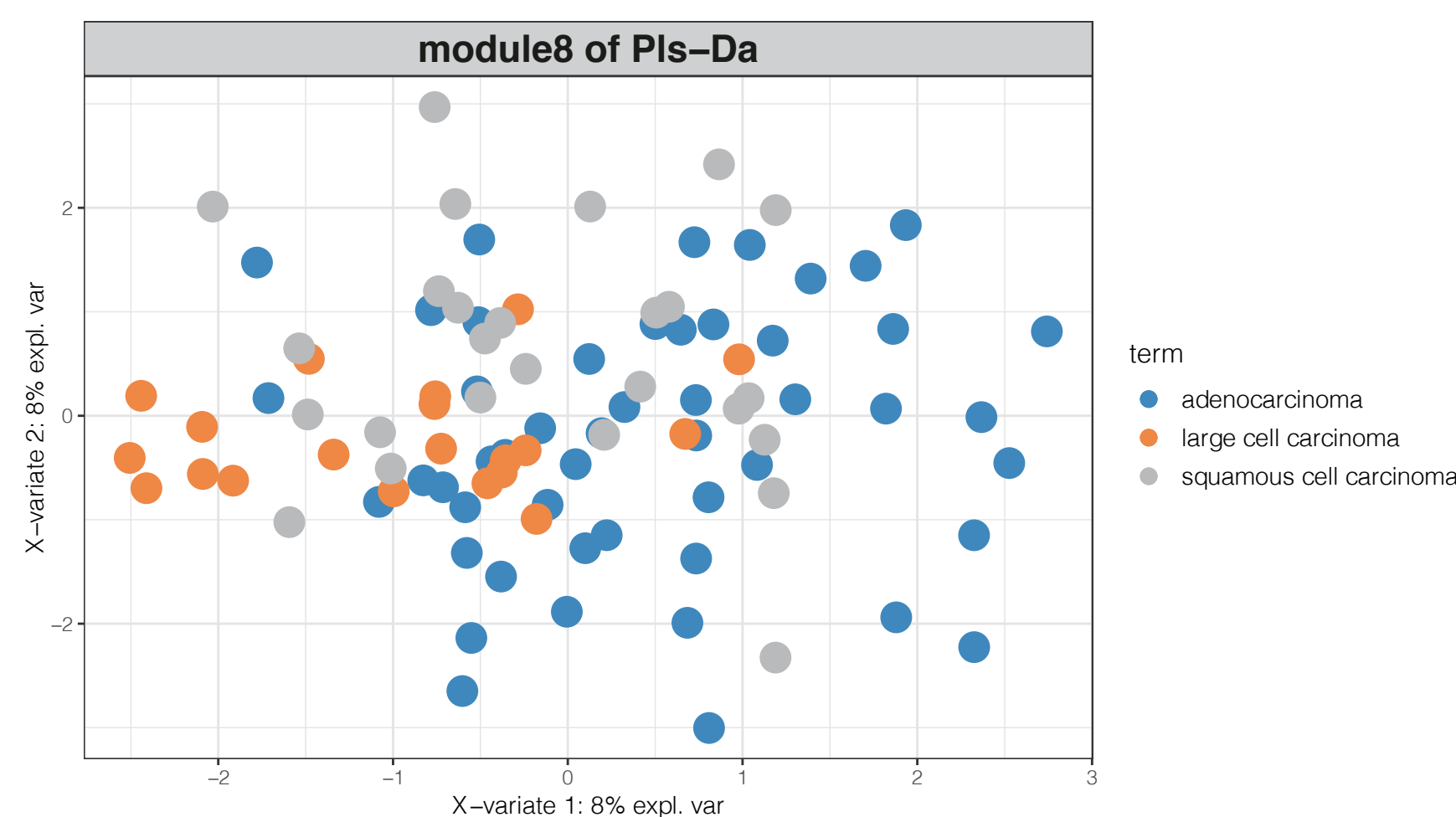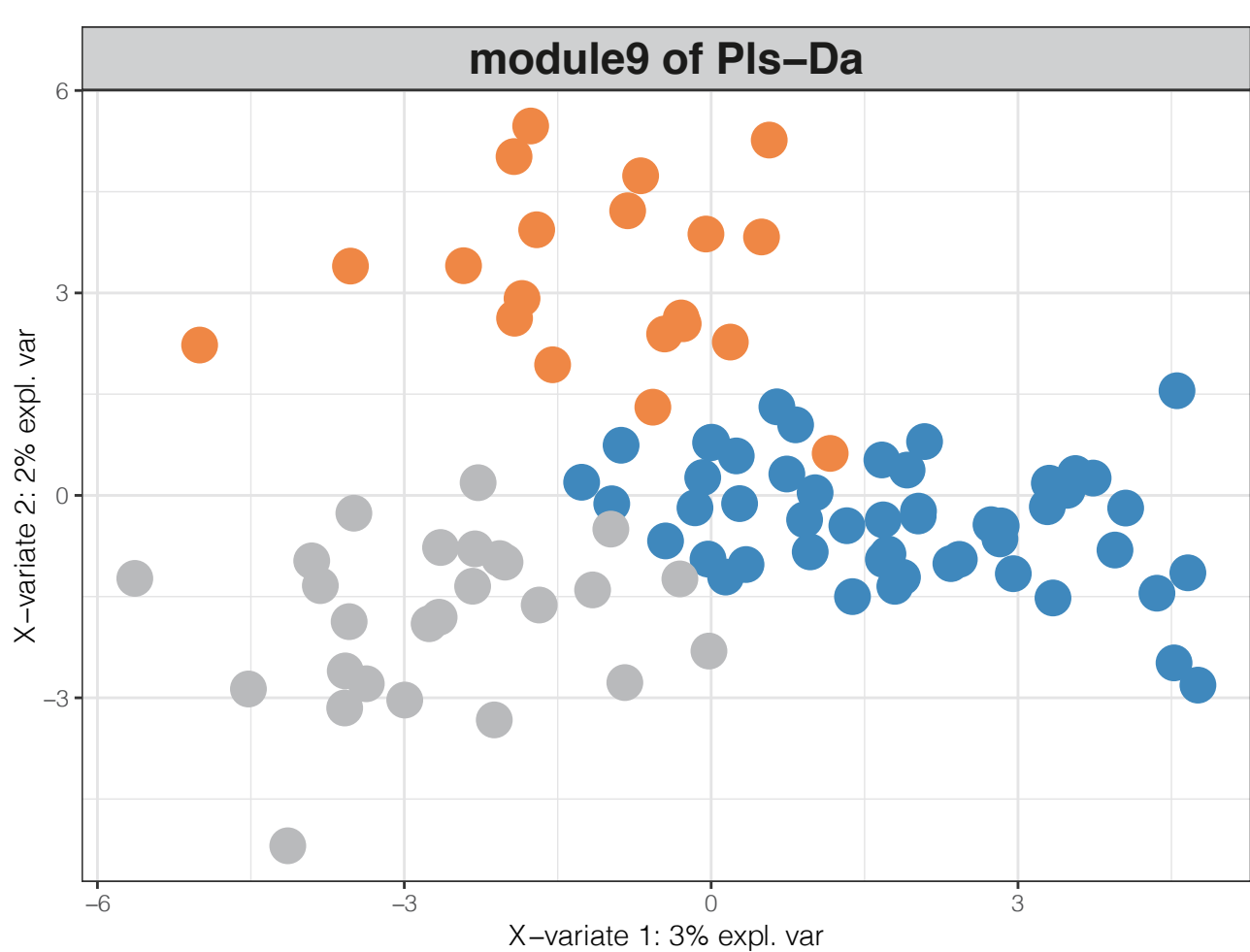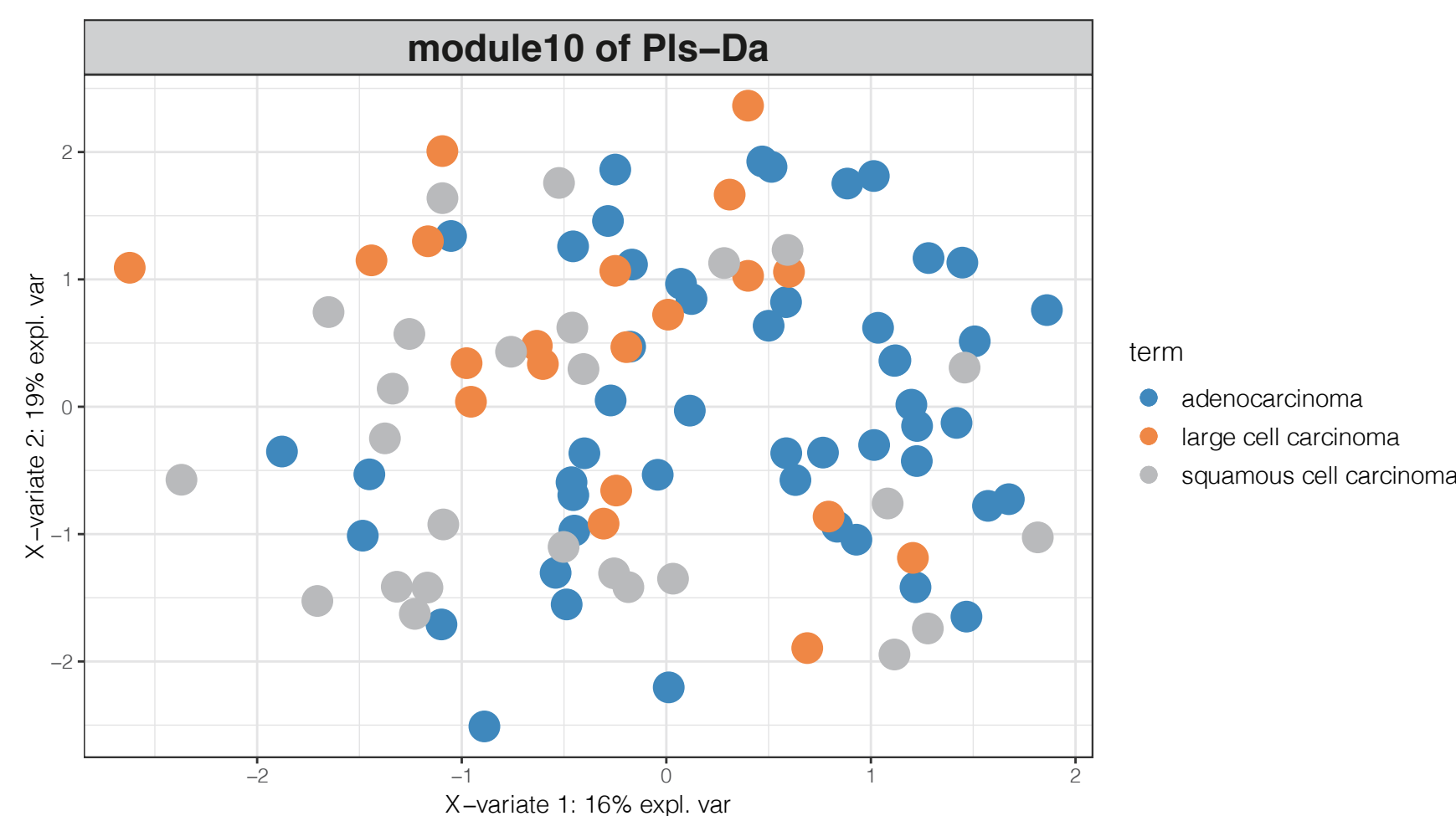

Supplement: Supplementary file 1 [file DataSheet1.ZIP › SI-figure/Figure S11.pdf]

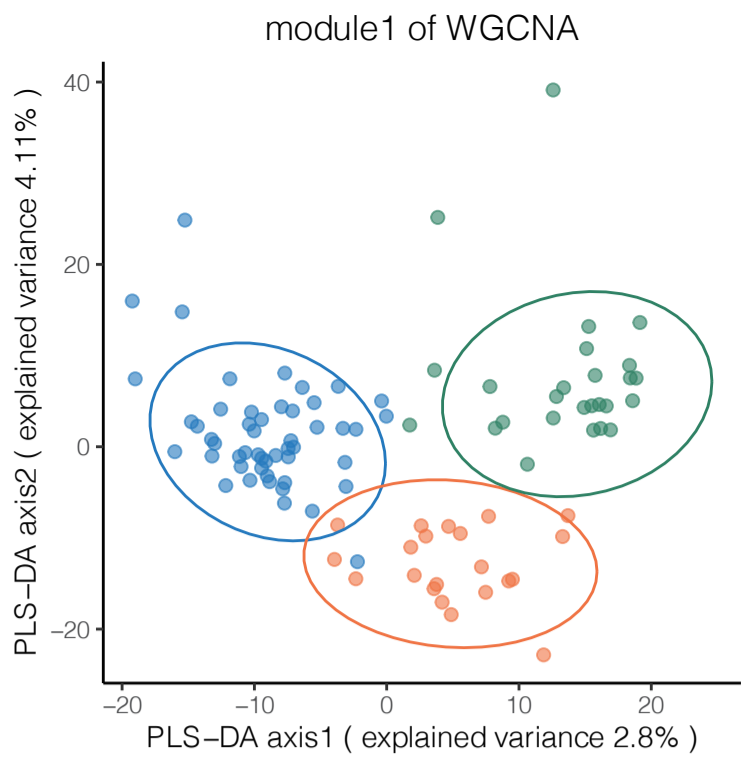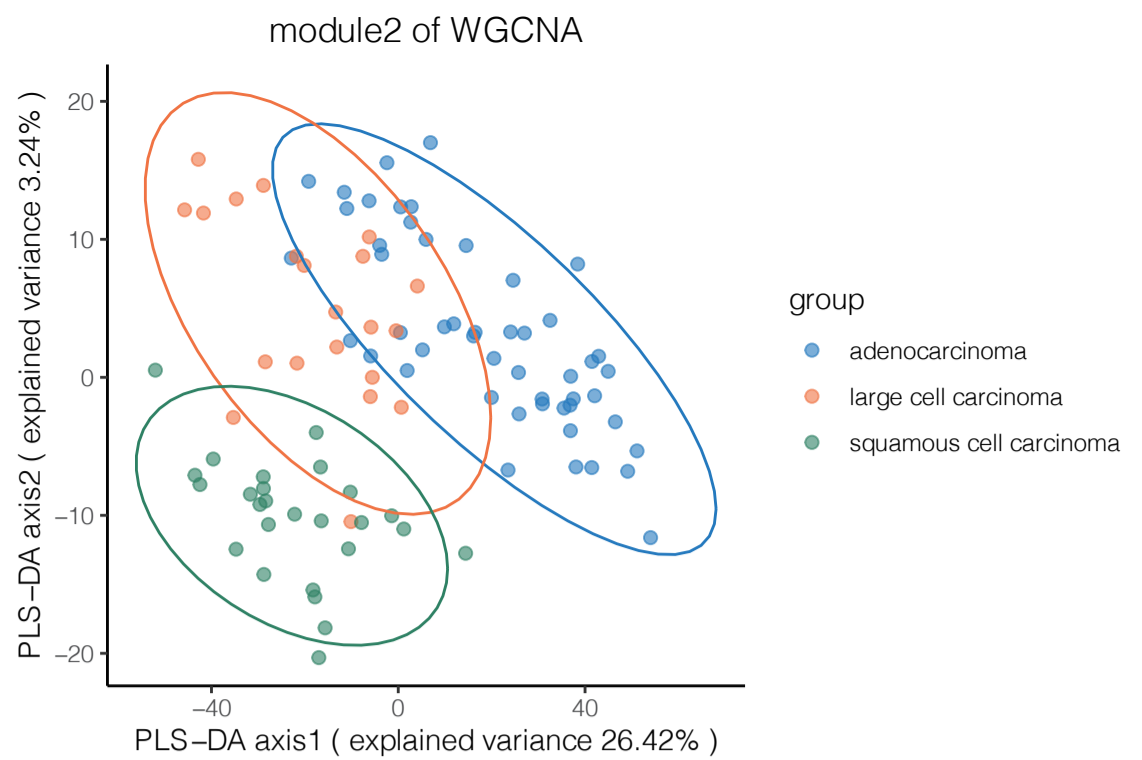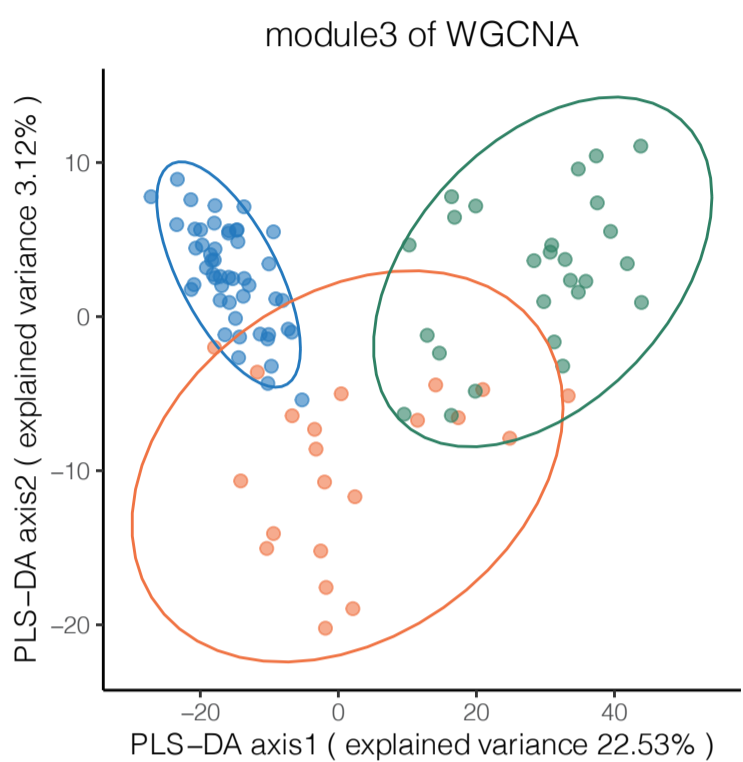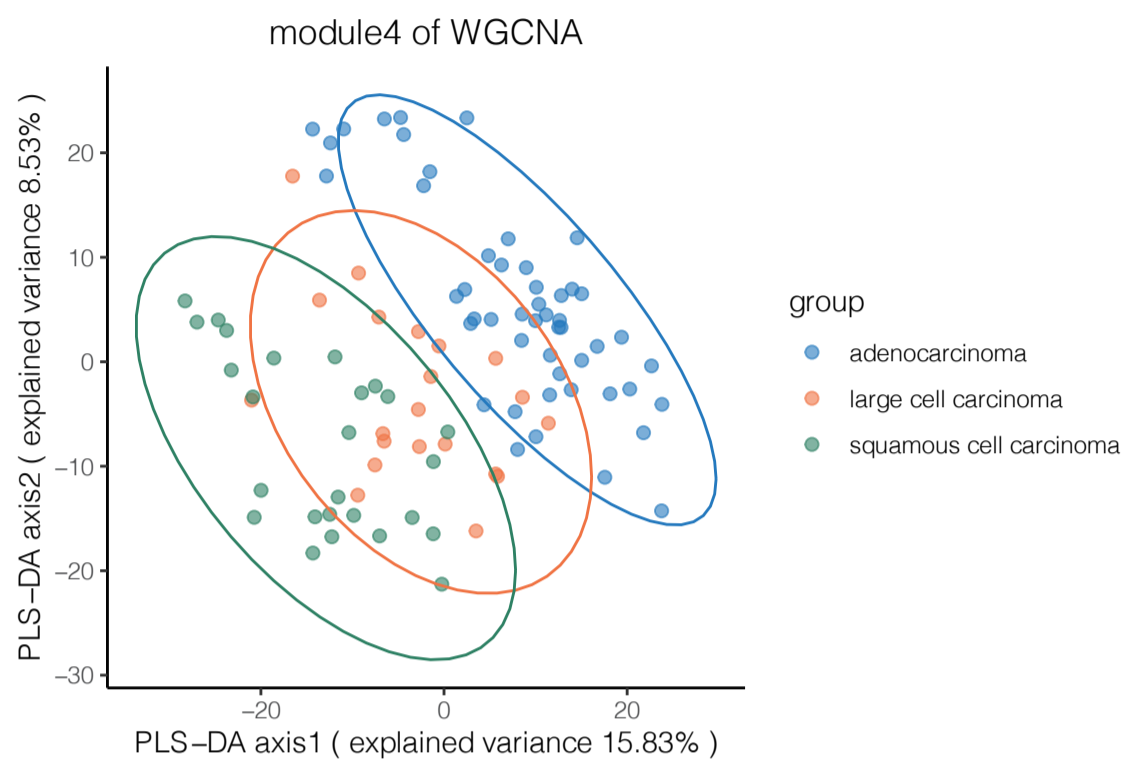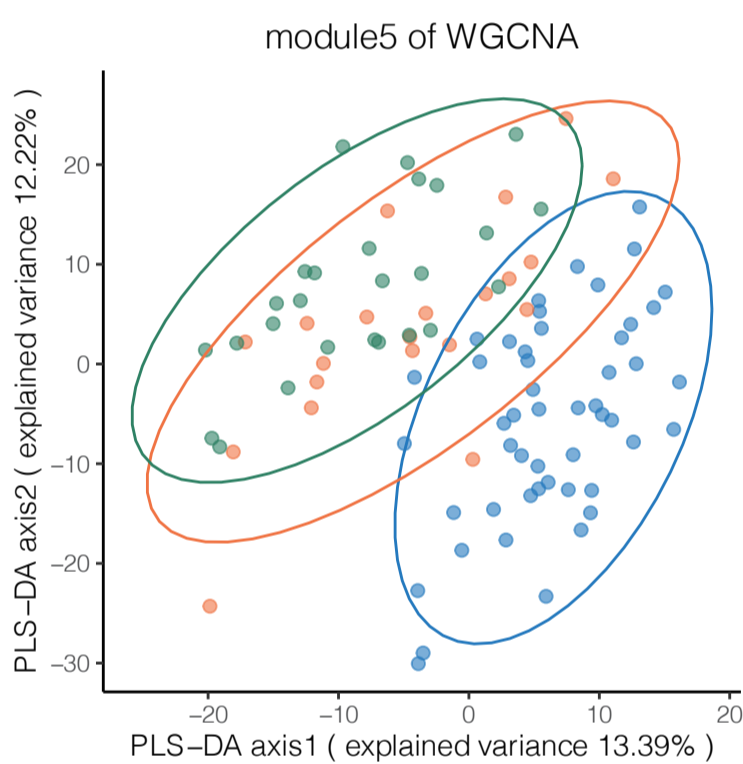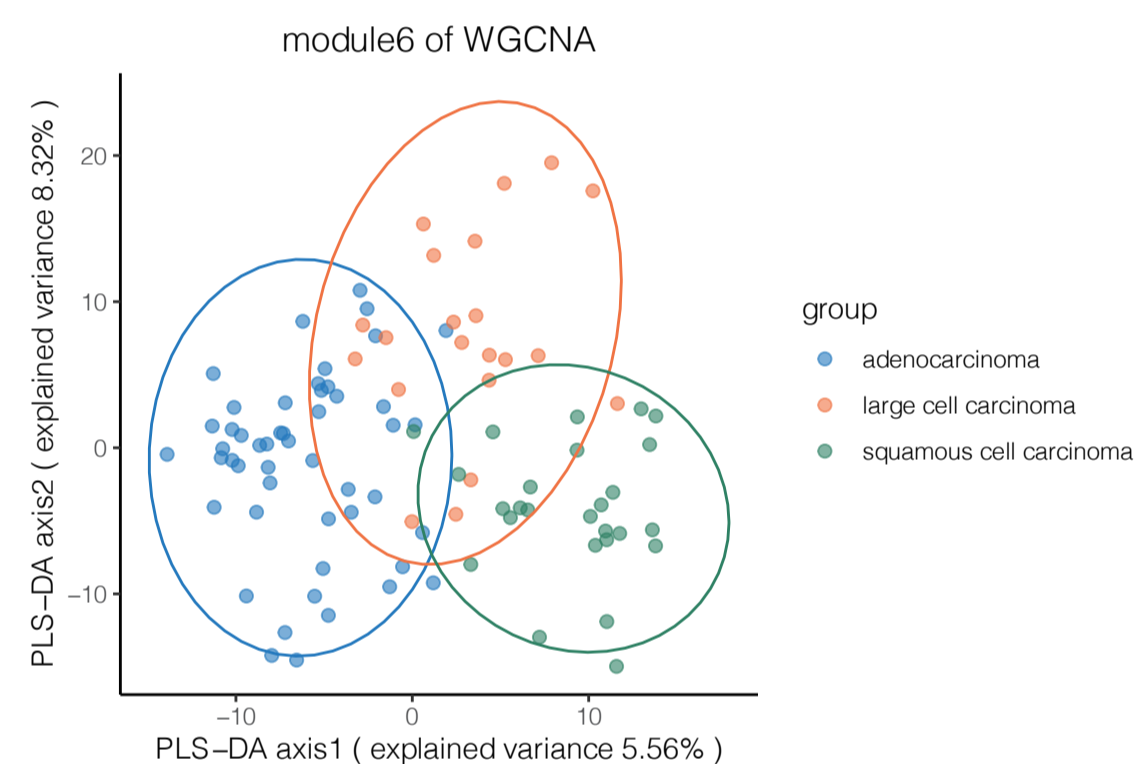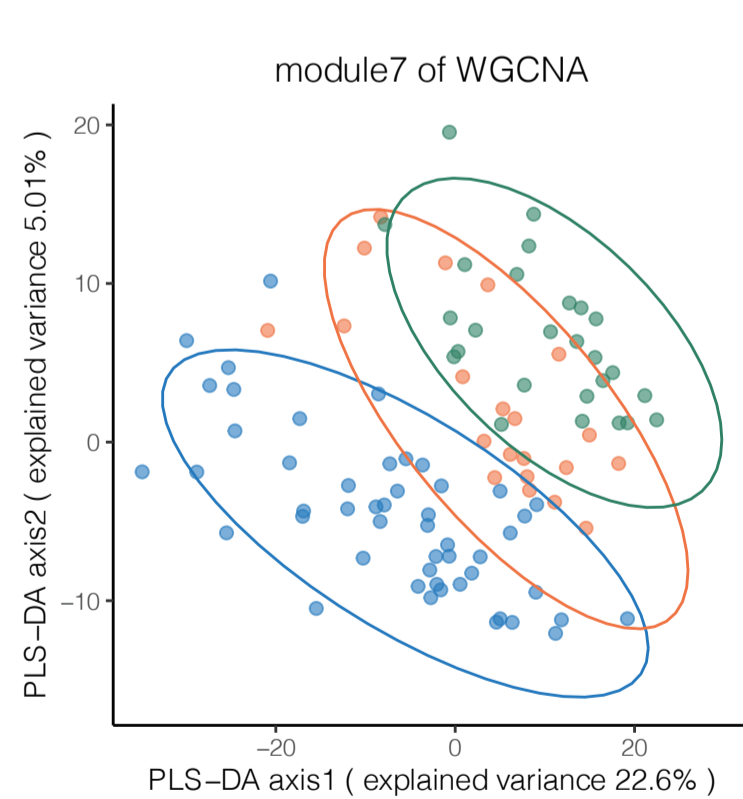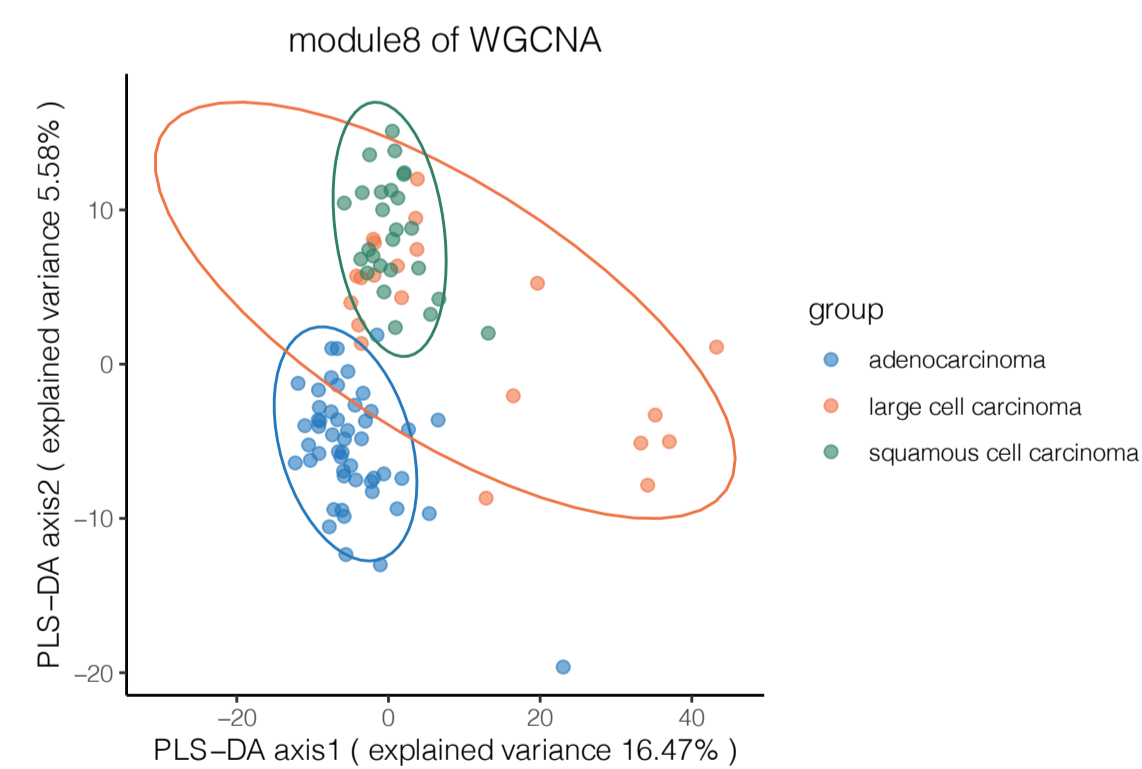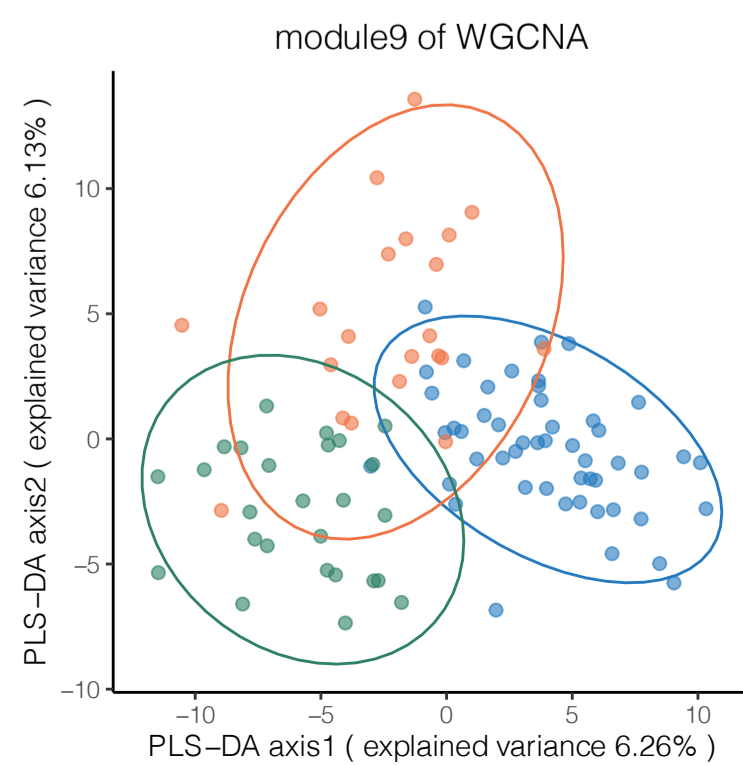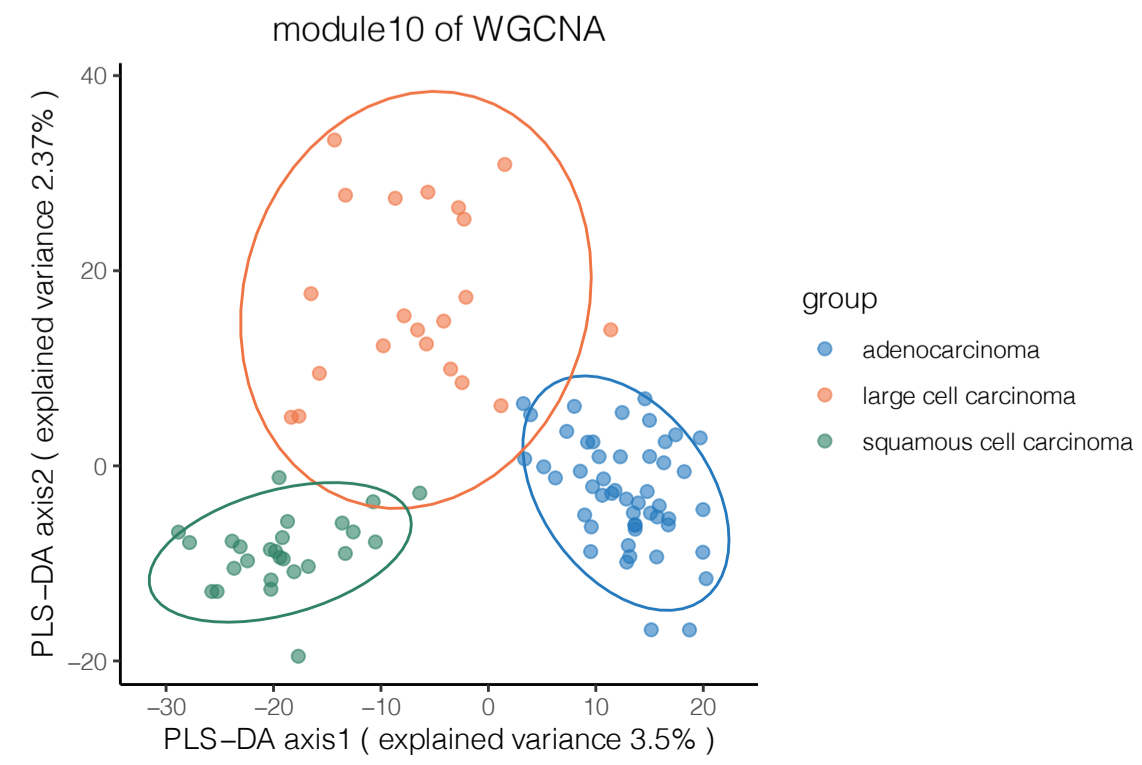

Supplement: Supplementary file 1 [file DataSheet1.ZIP › SI-figure/Figure S12.pdf]

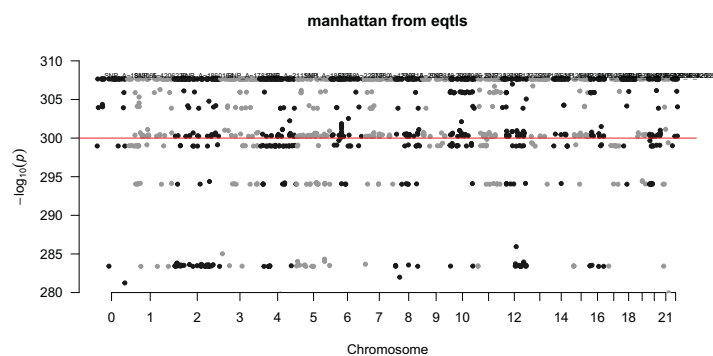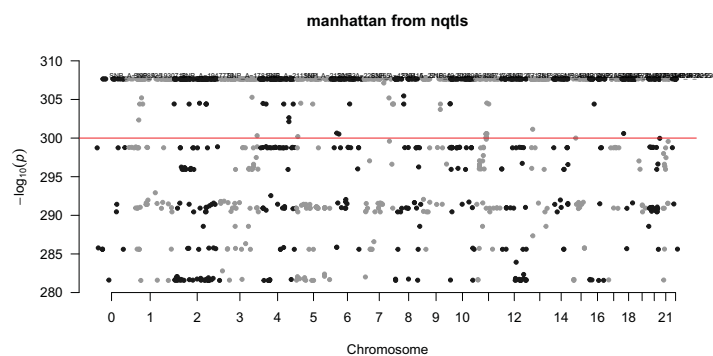

Supplement: Supplementary file 1 [file DataSheet1.ZIP › SI-figure/Figure S16.pdf]

nqtl-eqtl snps venn plot

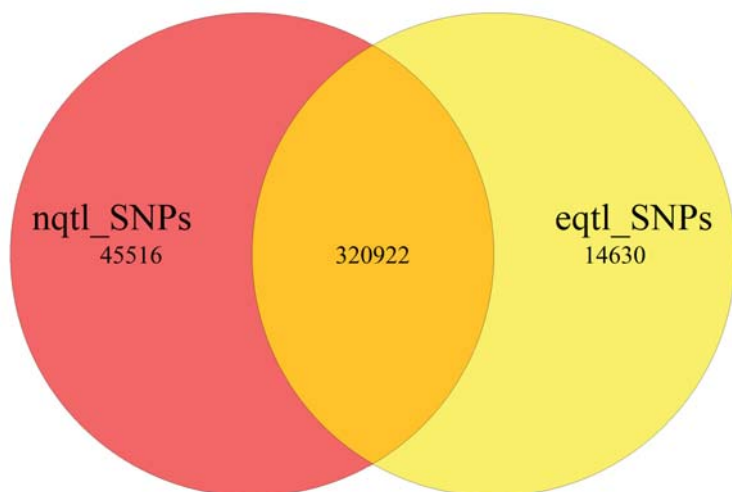

nqtl-eqtl genes venn plot

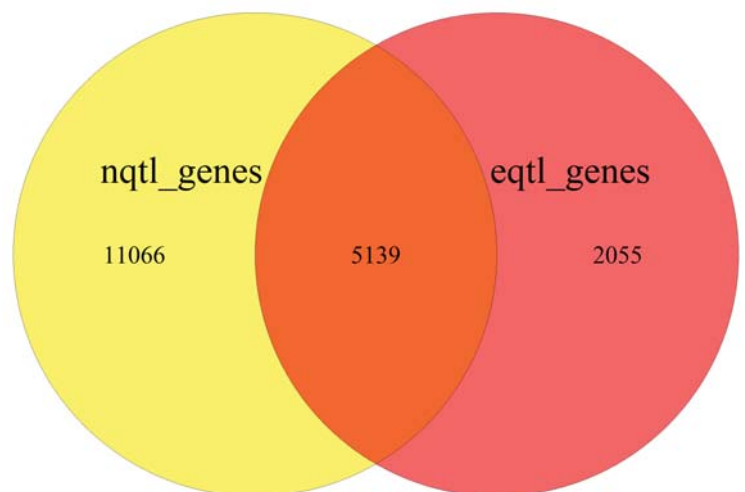

Supplement: Supplementary file 1 [file DataSheet1.ZIP › SI-figure/Figure S17.pdf]

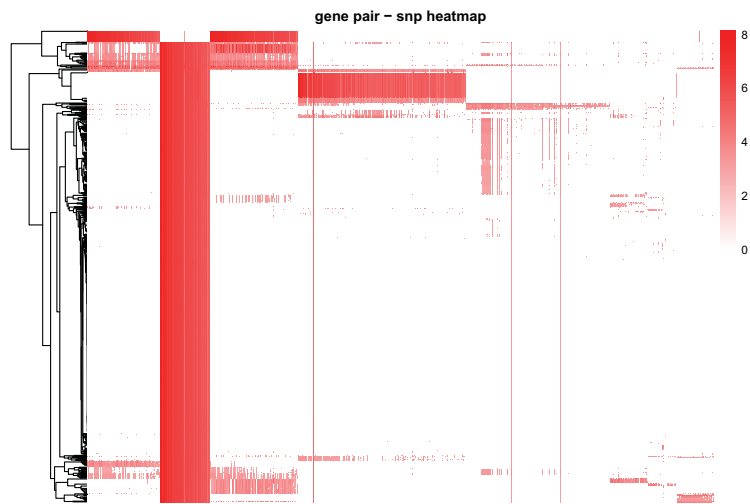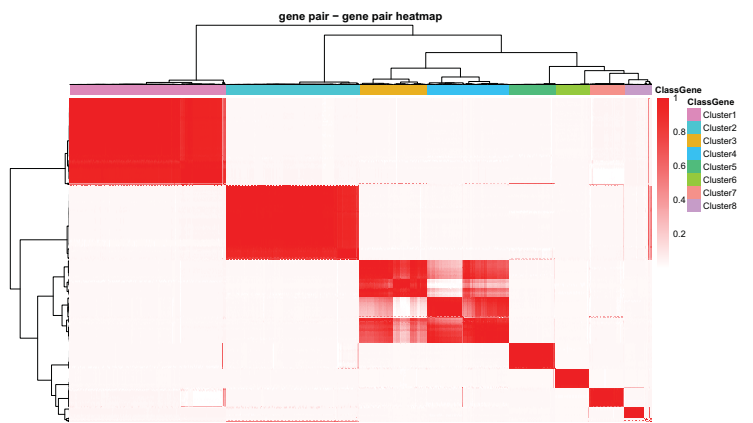

Supplement: Supplementary file 1 [file DataSheet1.ZIP › SI-figure/Figure S18.pdf]

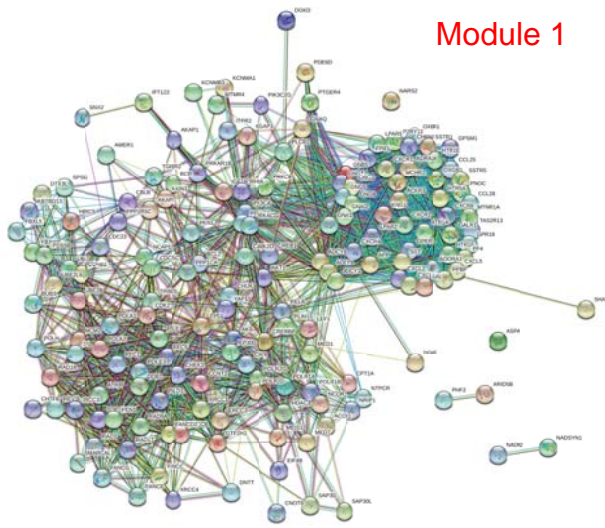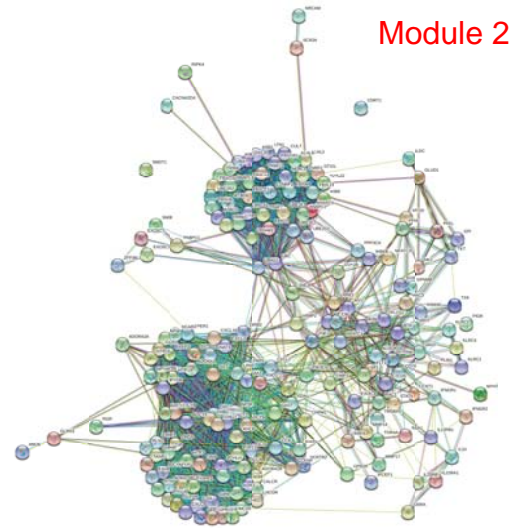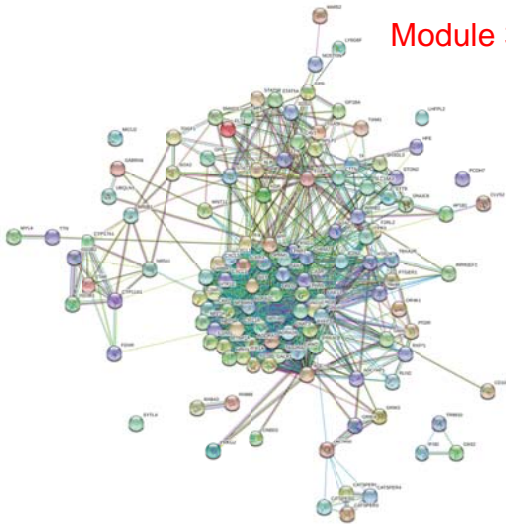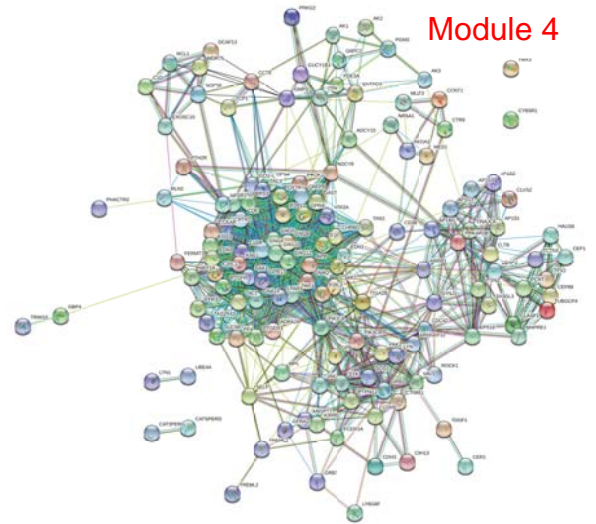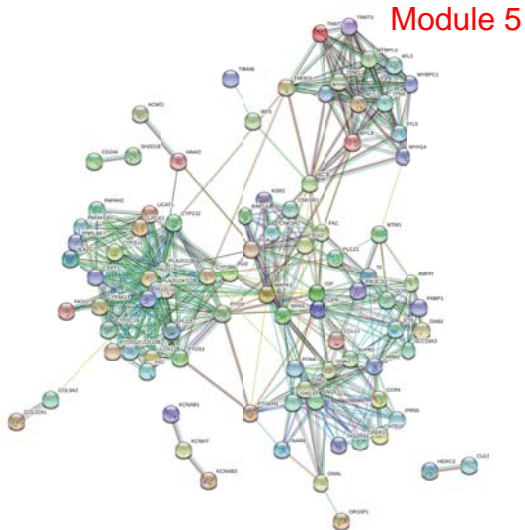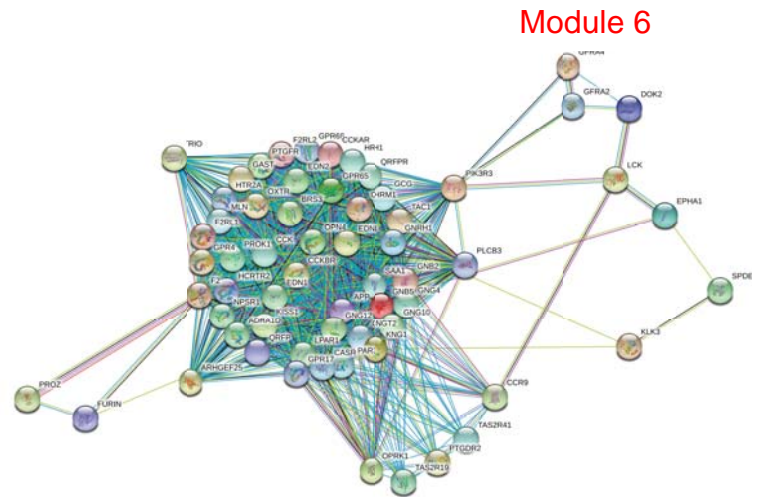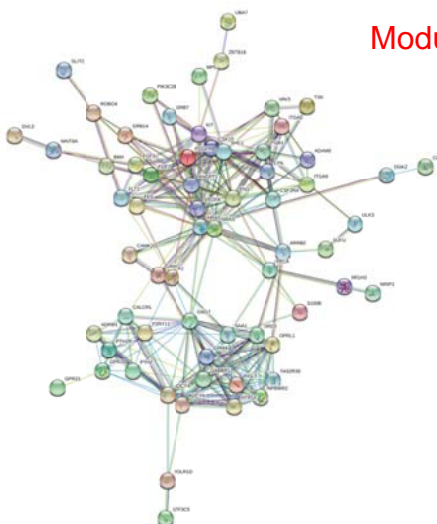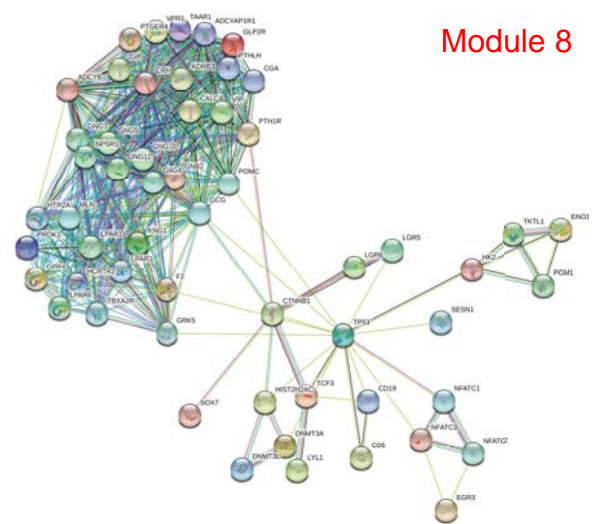

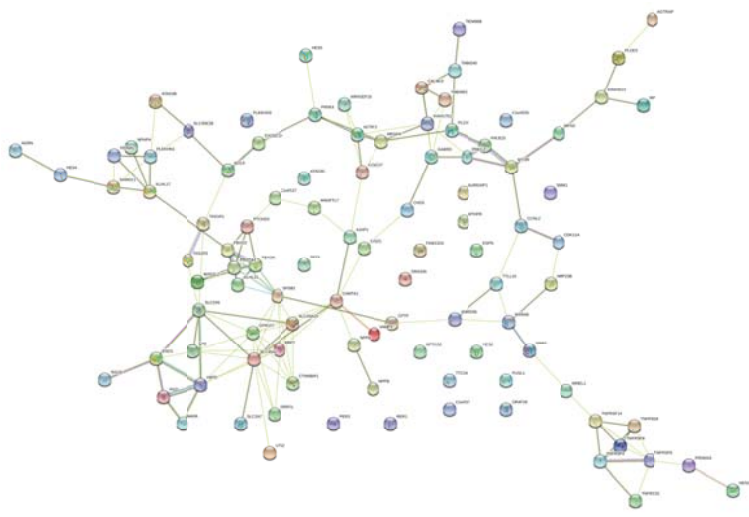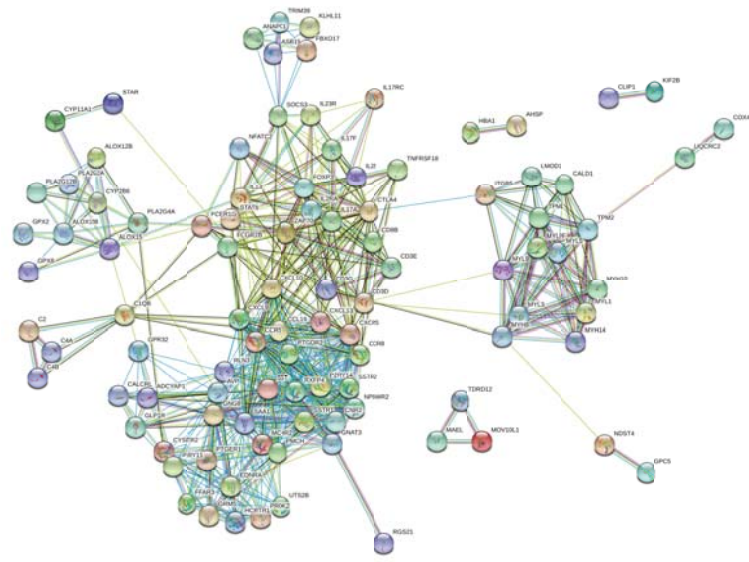

Supplement: Supplementary file 1 [file DataSheet1.ZIP › SI-figure/Figure S19.pdf]

**(A)****eQTL QQ-plot on GSE28571**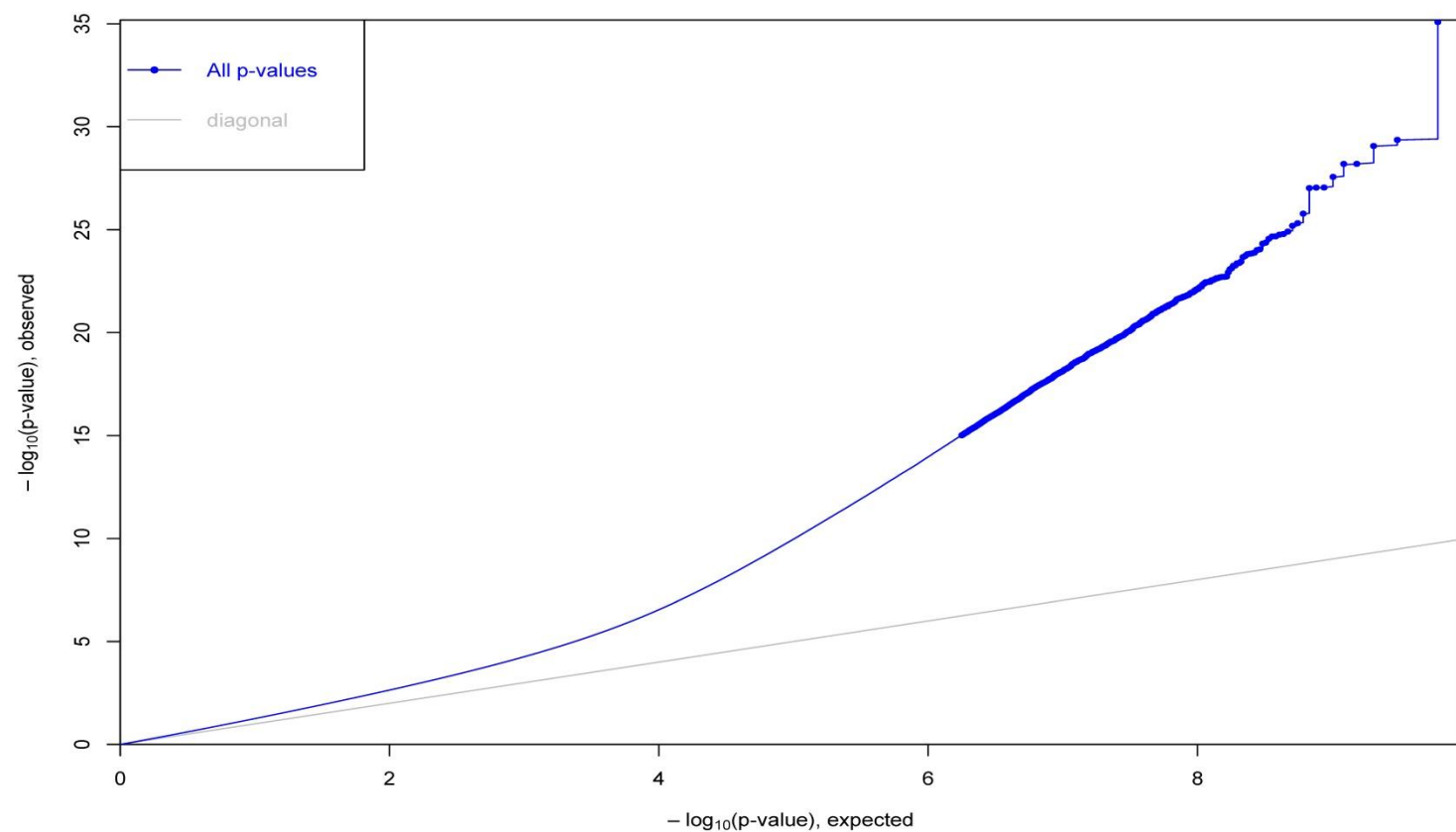**(B)****nQTL QQ-plot on GSE28571**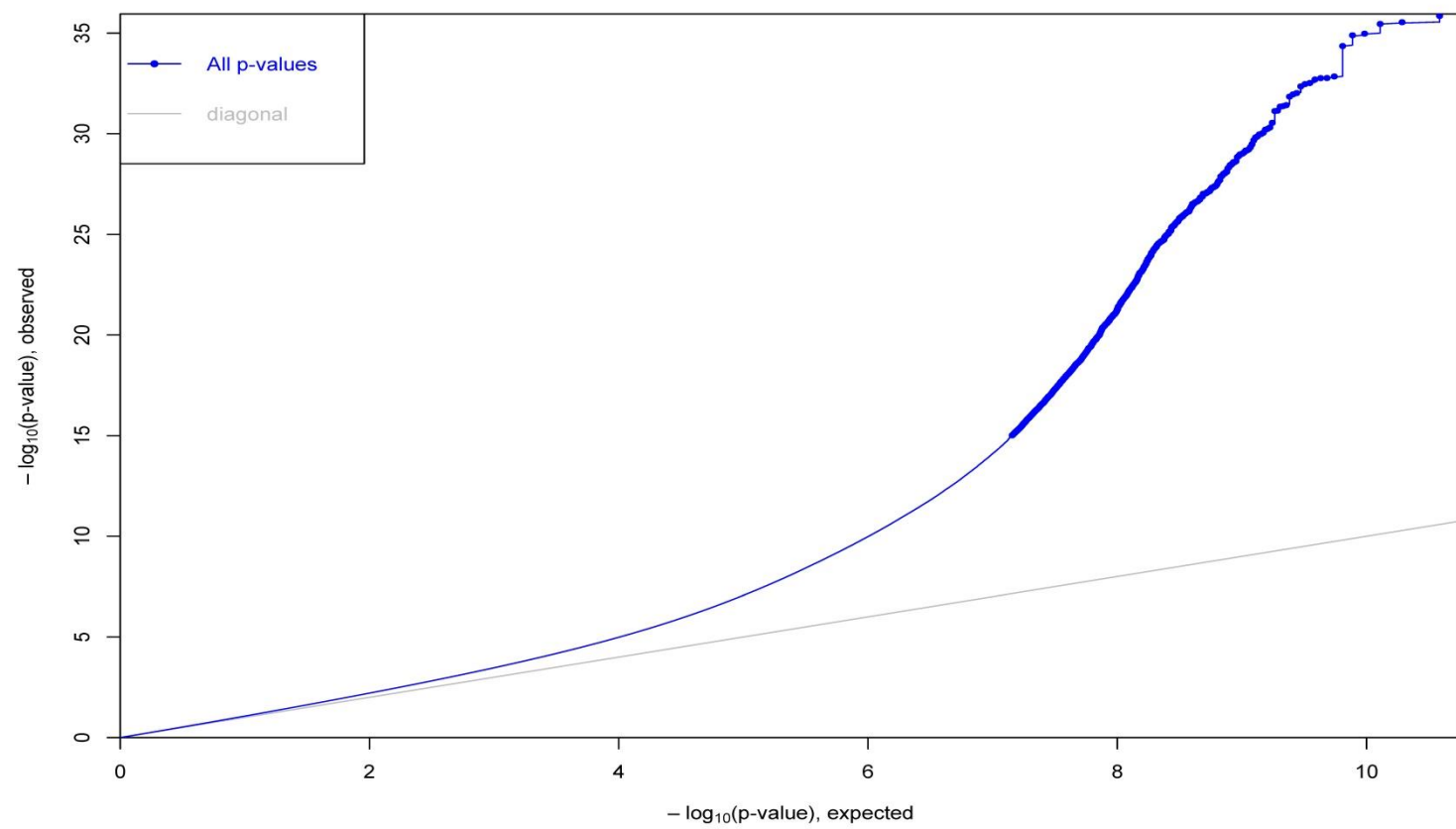

Supplement: Supplementary file 1 [file DataSheet1.ZIP › SI-figure/Figure S2.pdf]

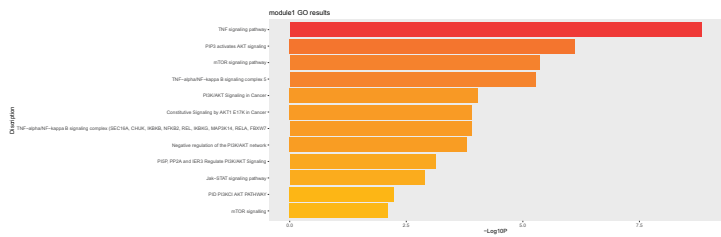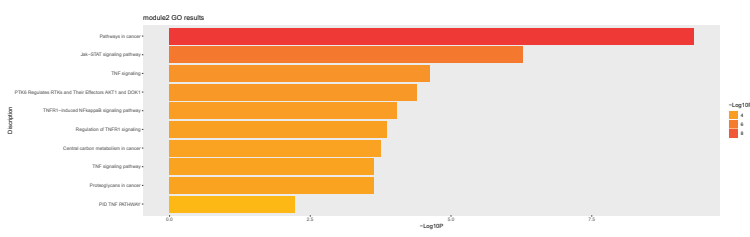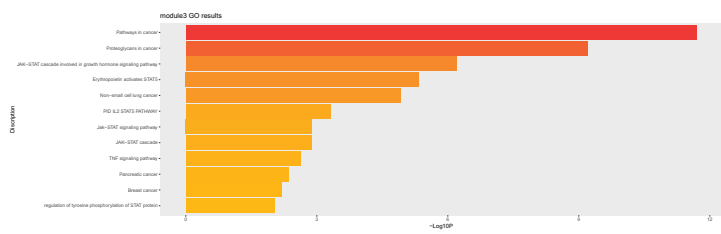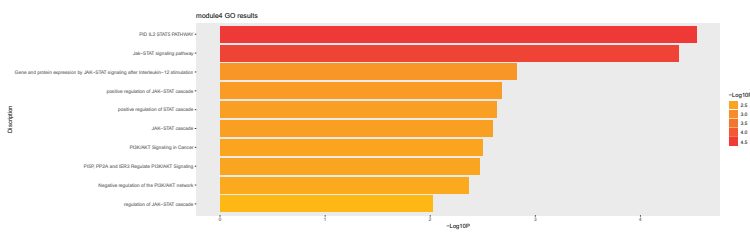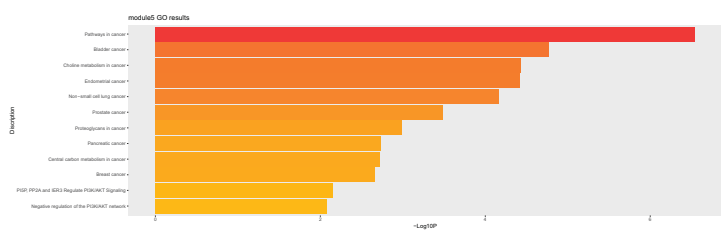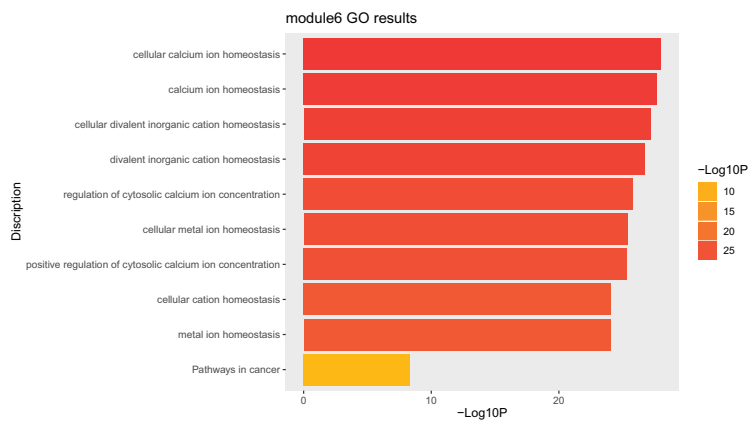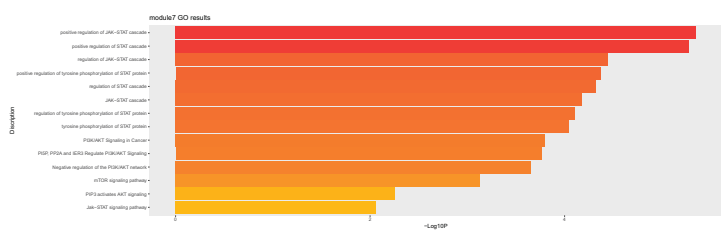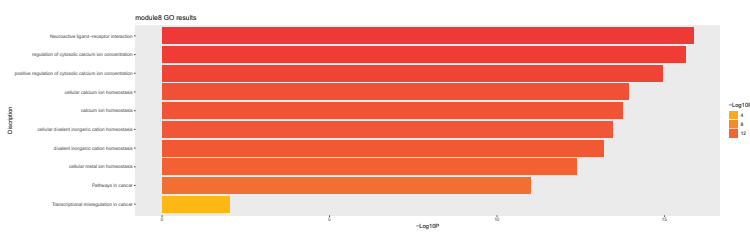

Supplement: Supplementary file 1 [file DataSheet1.ZIP › SI-figure/Figure S20.pdf]

**(A)****eQTL QQ-plot on scRNA-seq induced data**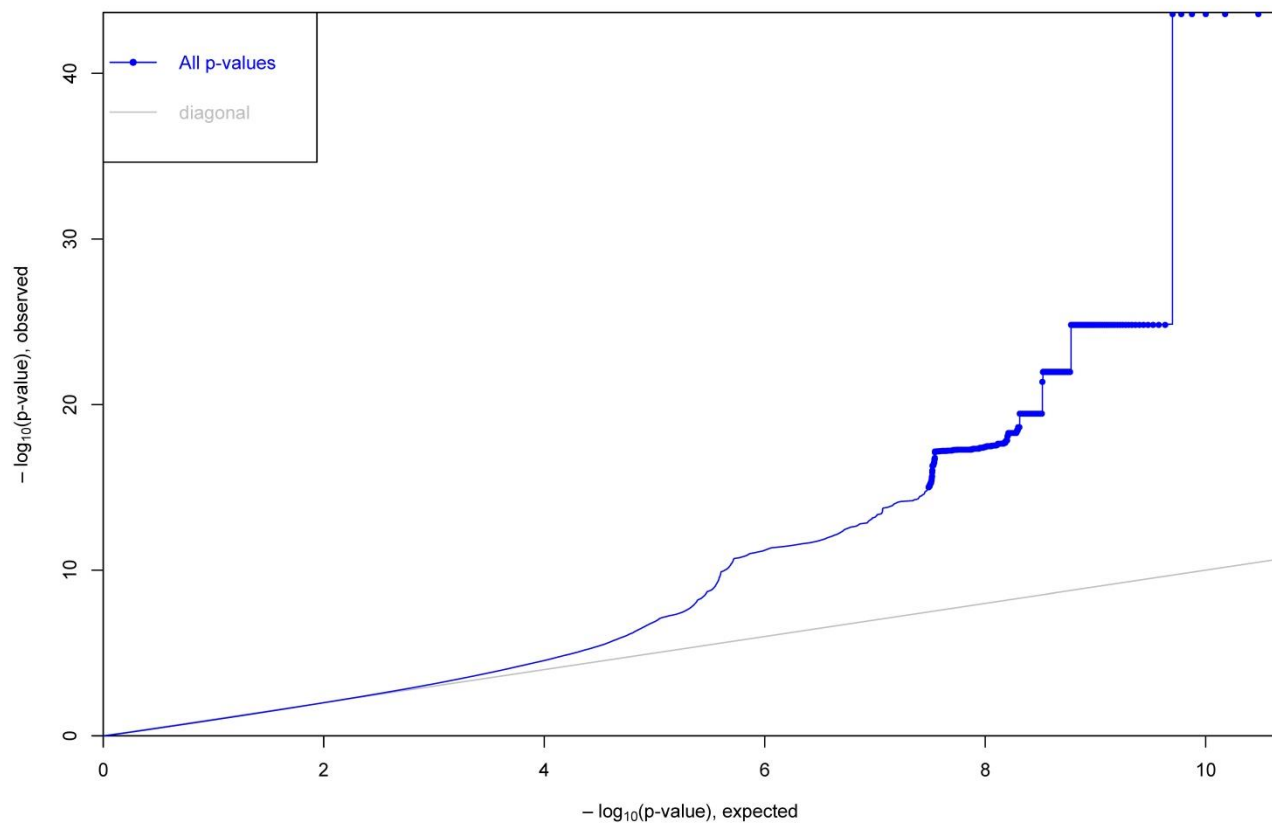**(B)****nQTL QQ-plot on scRNA-seq induced data**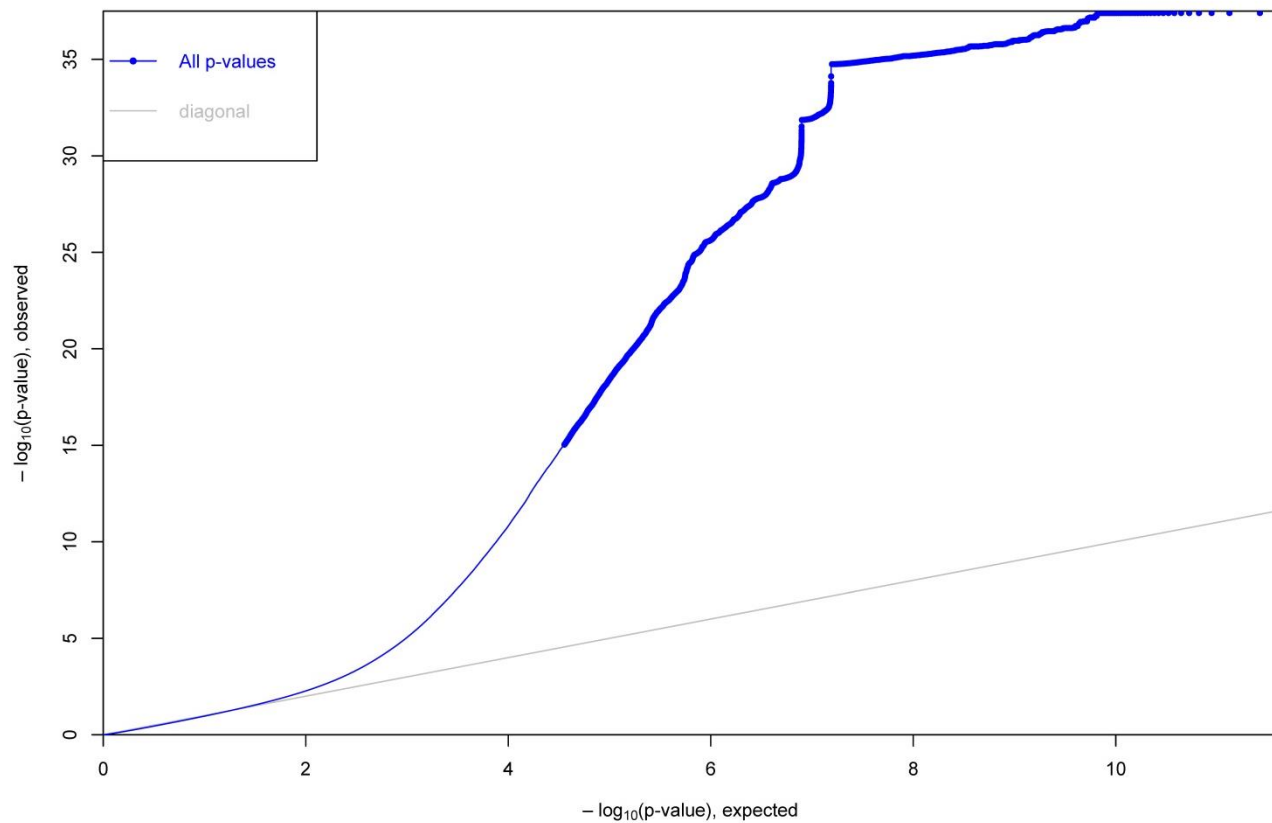

Supplement: Supplementary file 1 [file DataSheet1.ZIP › SI-figure/Figure S23.pdf]

## eQTL Manhattan plot on GSE28571

(A)

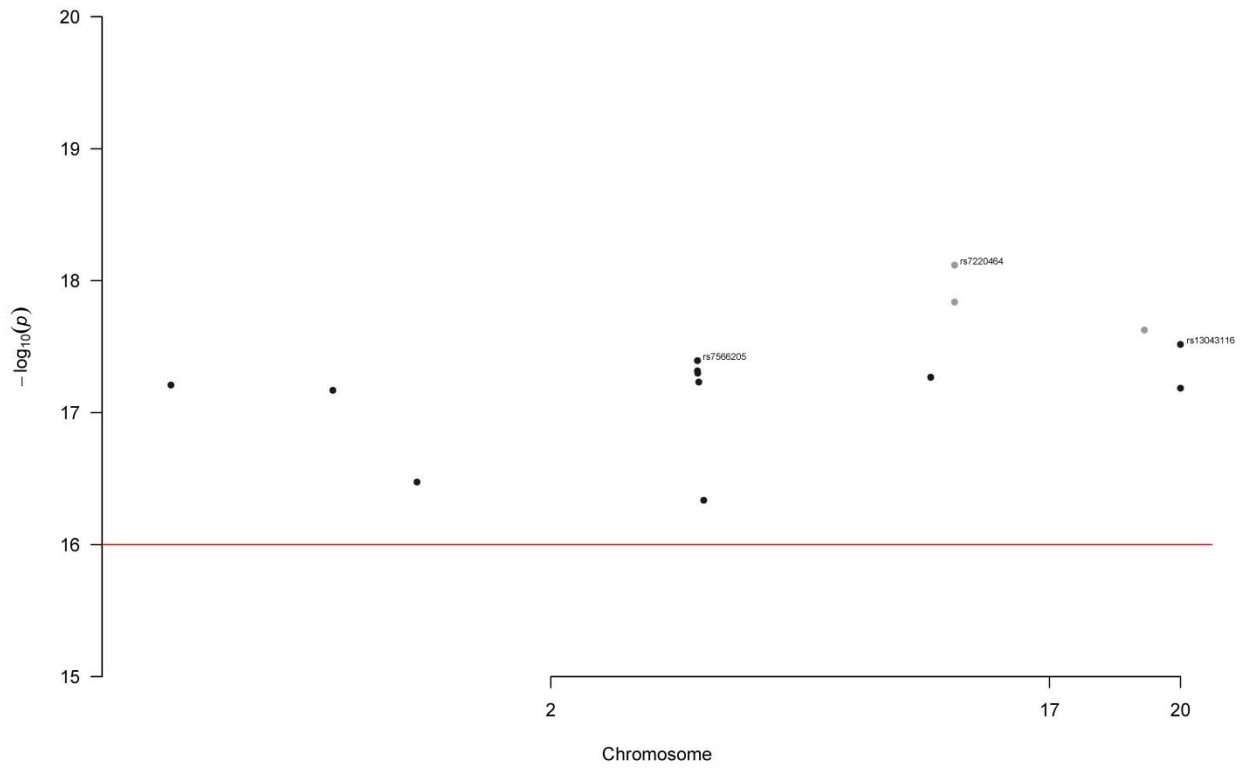

## nQTL Manhattan plot on GSE28571

(B)

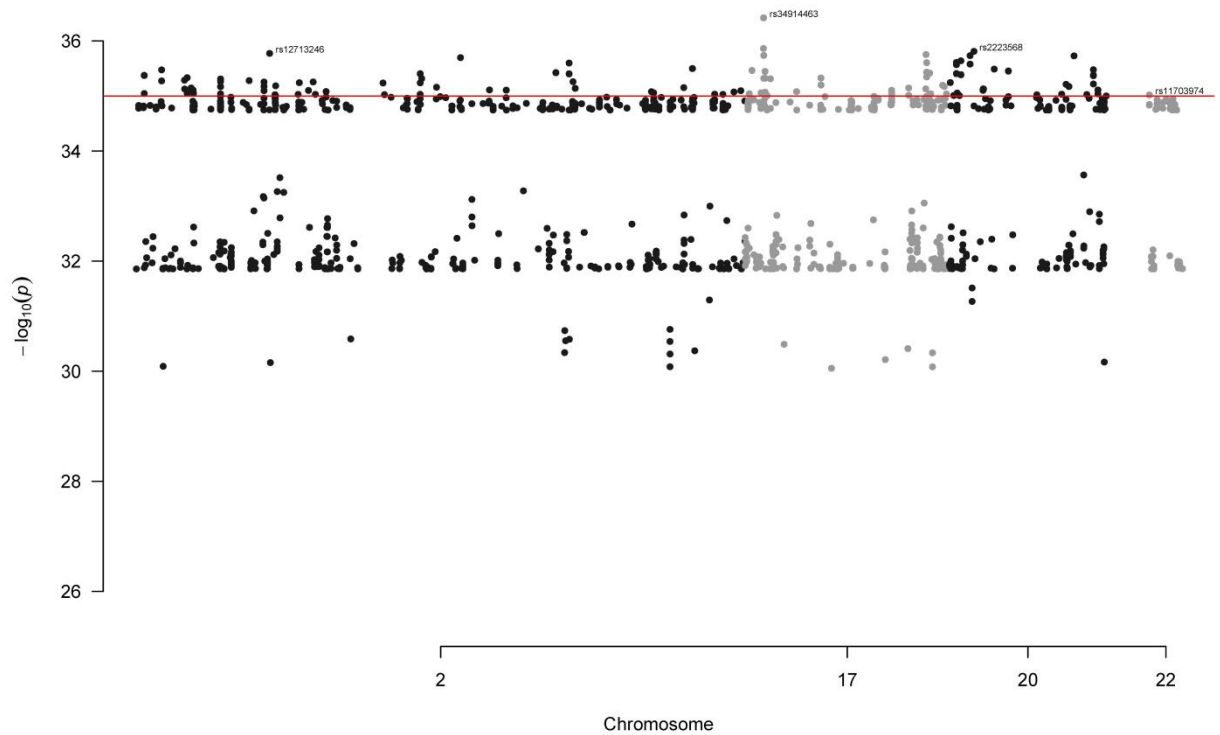

Supplement: Supplementary file 1 [file DataSheet1.ZIP › SI-figure/Figure S24.pdf]

nqtl-eqtl snps venn plot

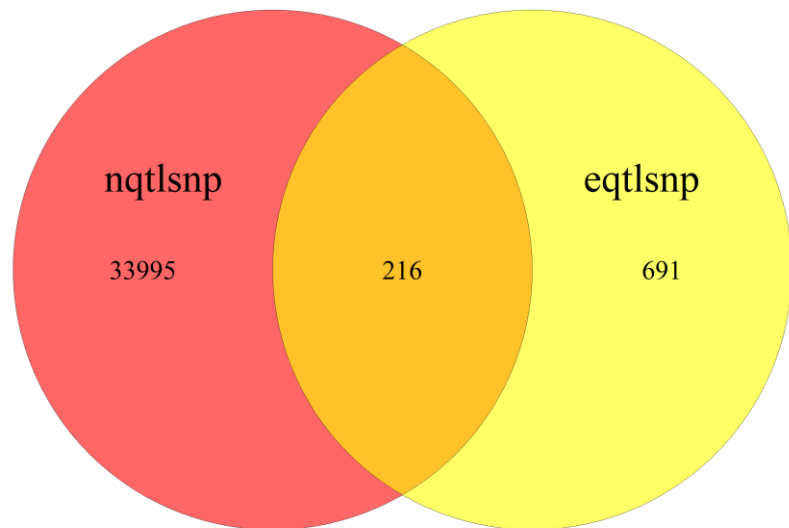

**(A)**

nqtl-eqtl genes venn plot

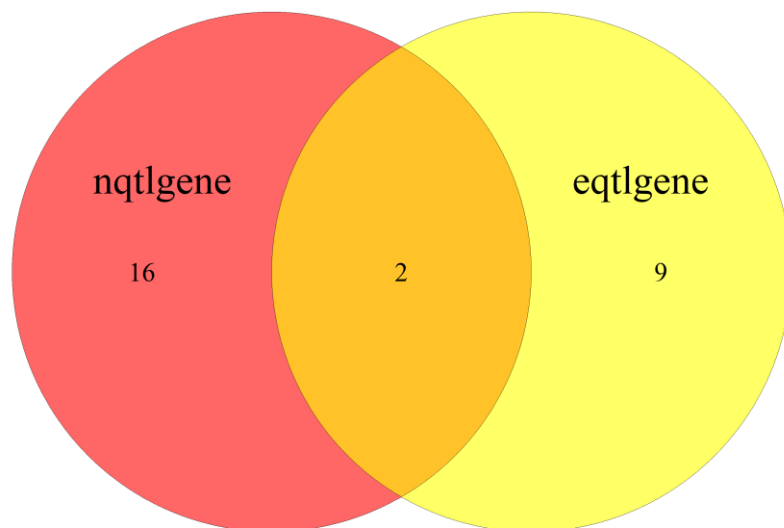

**(B)**

Supplement: Supplementary file 1 [file DataSheet1.ZIP › SI-figure/Figure S25.pdf]

Gene-pair & Snp association matrix

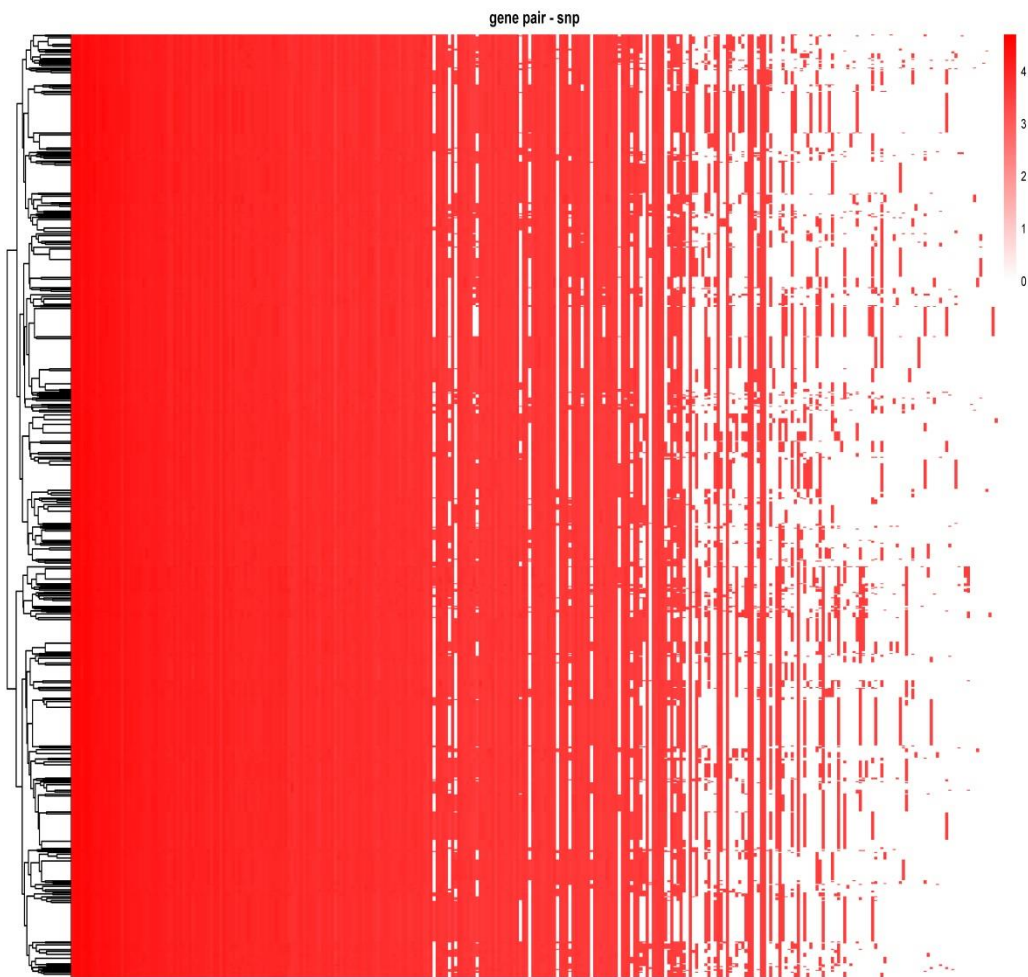

(A)

Gene-pair association matrix

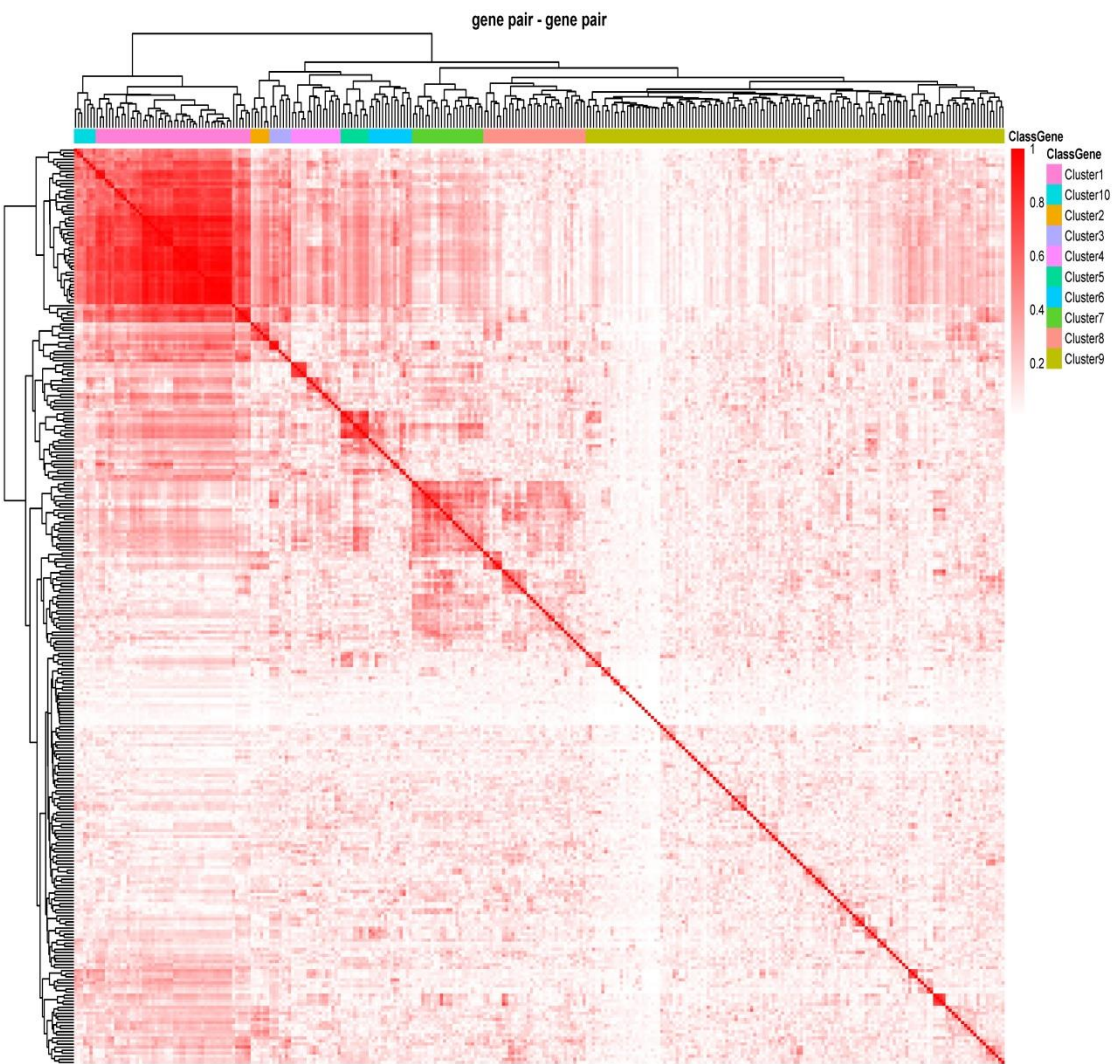

(B)

Supplement: Supplementary file 1 [file DataSheet1.ZIP › SI-figure/Figure S26.pdf]

**(A)** **Module-1**

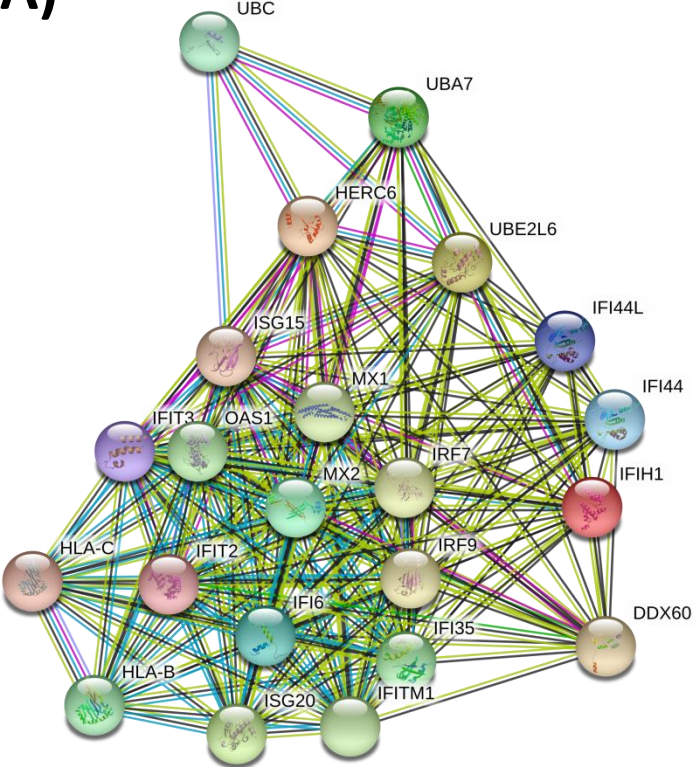

**(B)** **Module-4**

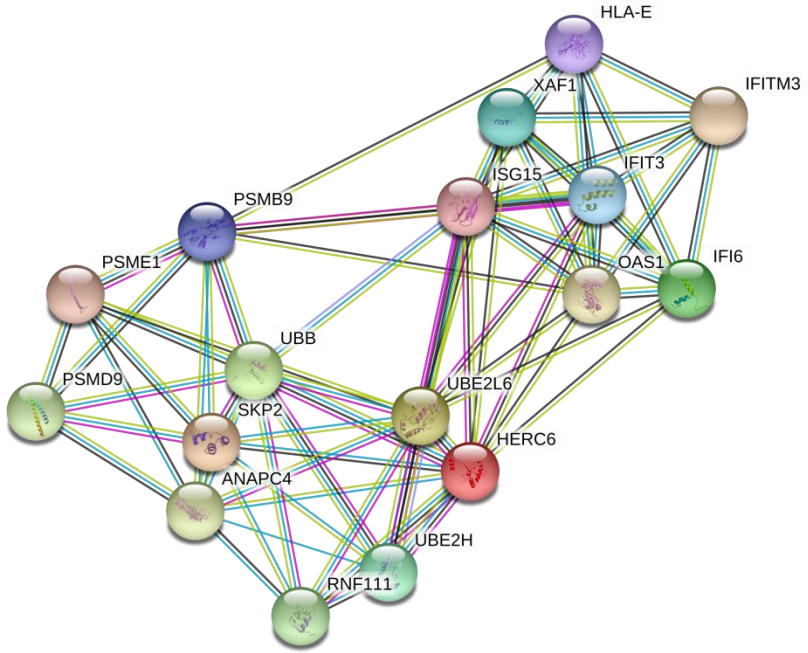

**(C)** **Module-7**

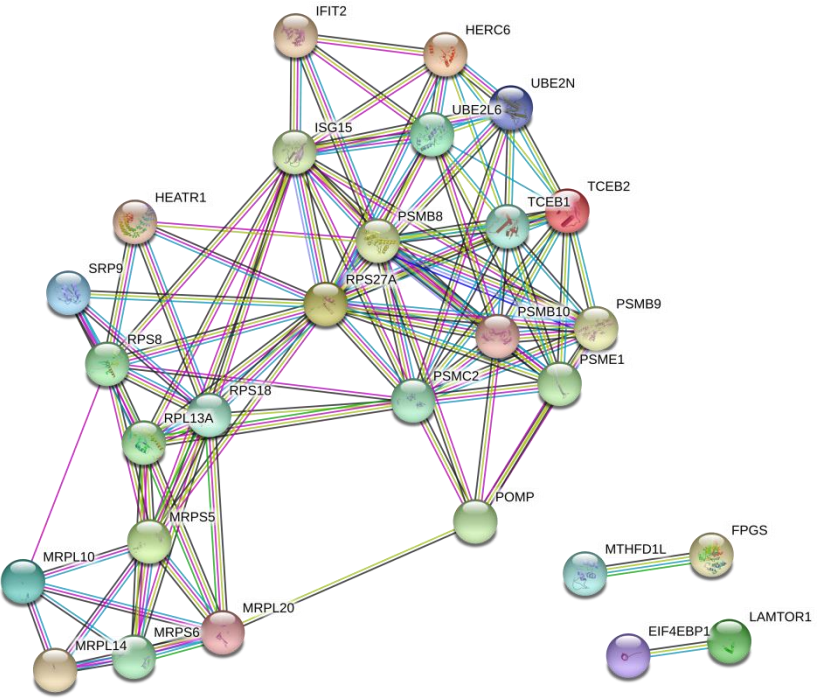

**(D)** **Module-9**

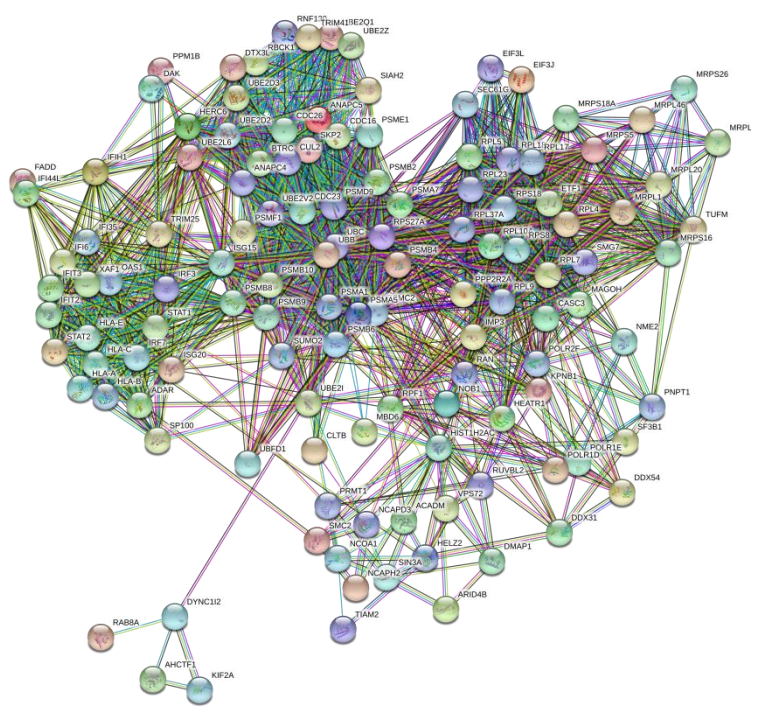

Supplement: Supplementary file 1 [file DataSheet1.ZIP › SI-figure/Figure S27.pdf]

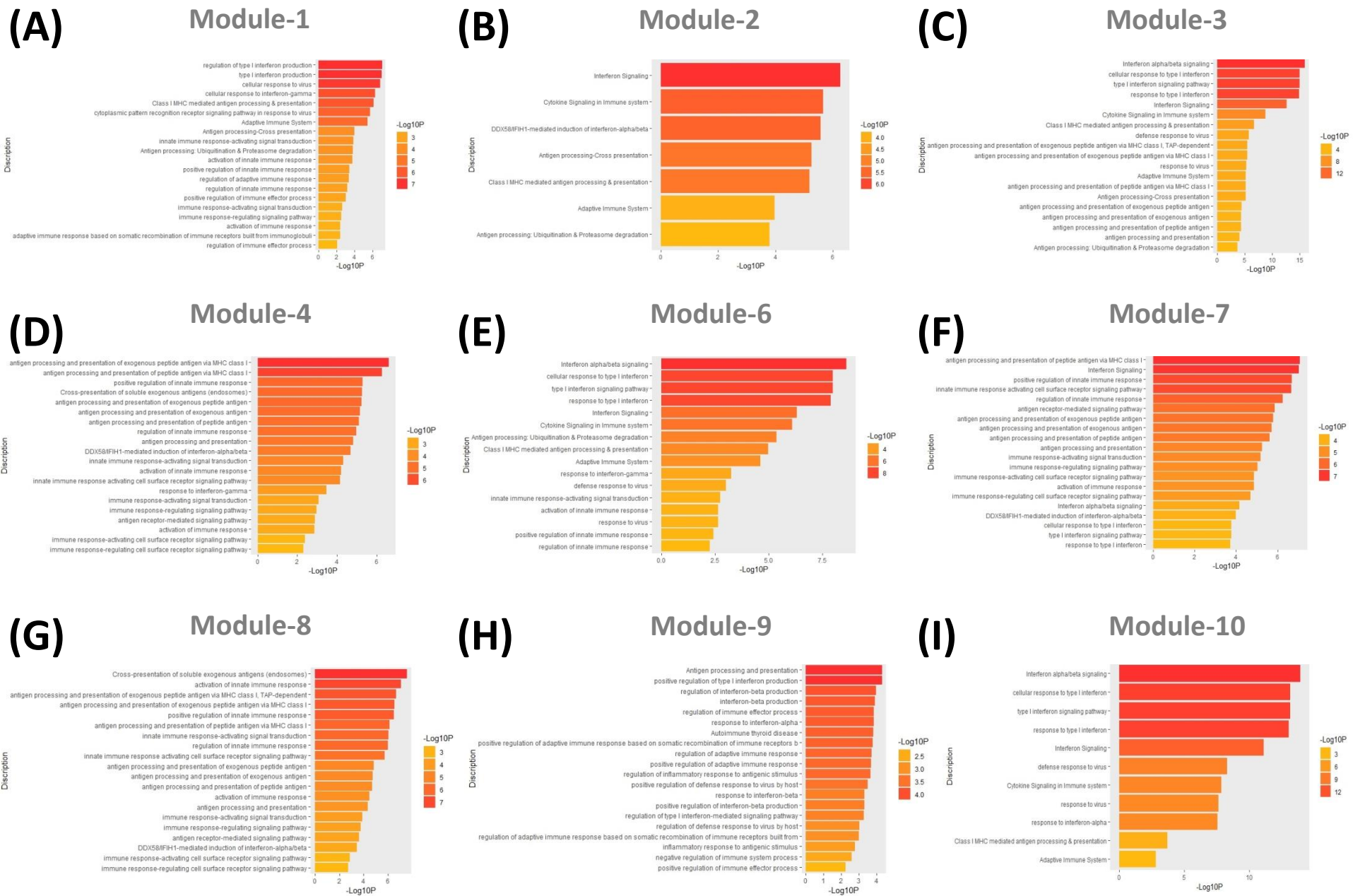

Supplement: Supplementary file 1 [file DataSheet1.ZIP › SI-figure/Figure S29.pdf]

# eQTL Manhattan plot on GSE28571

(A)

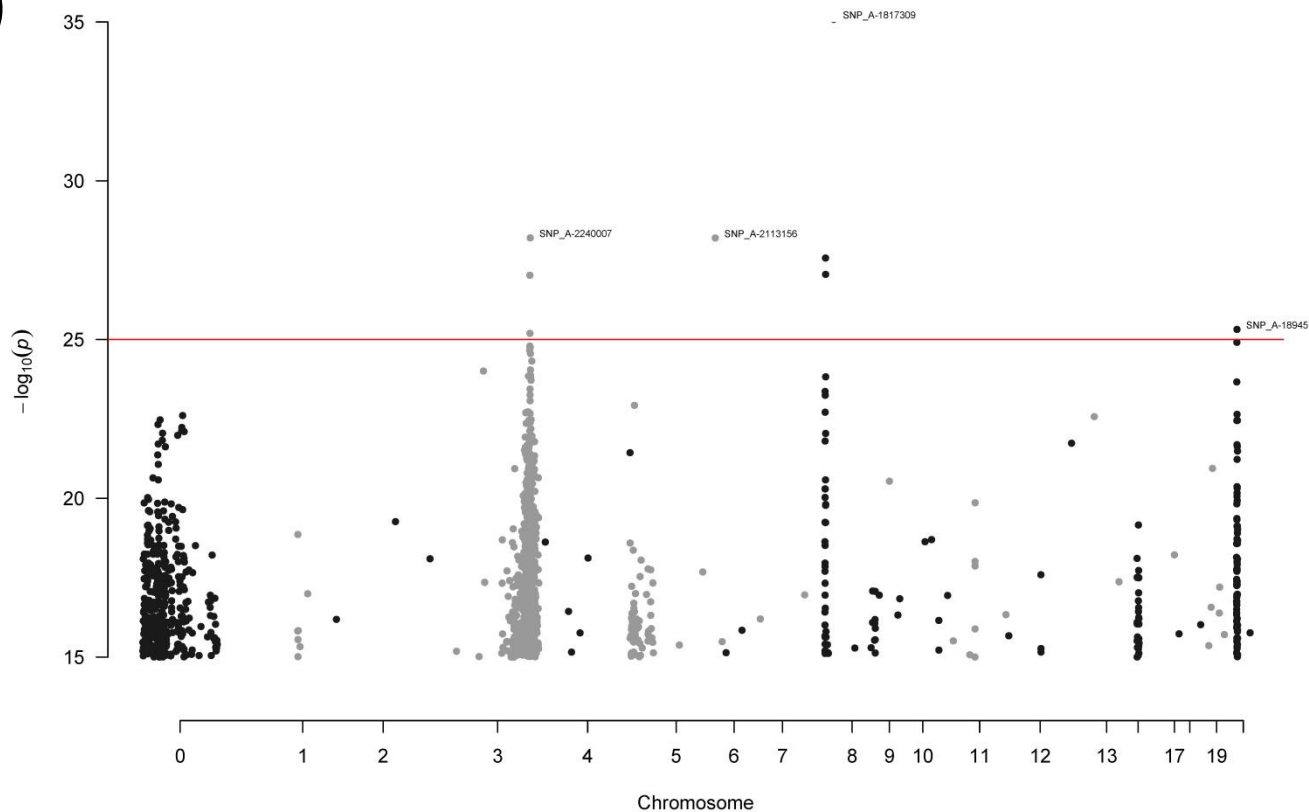

# nQTL Manhattan plot on GSE28571

(B)

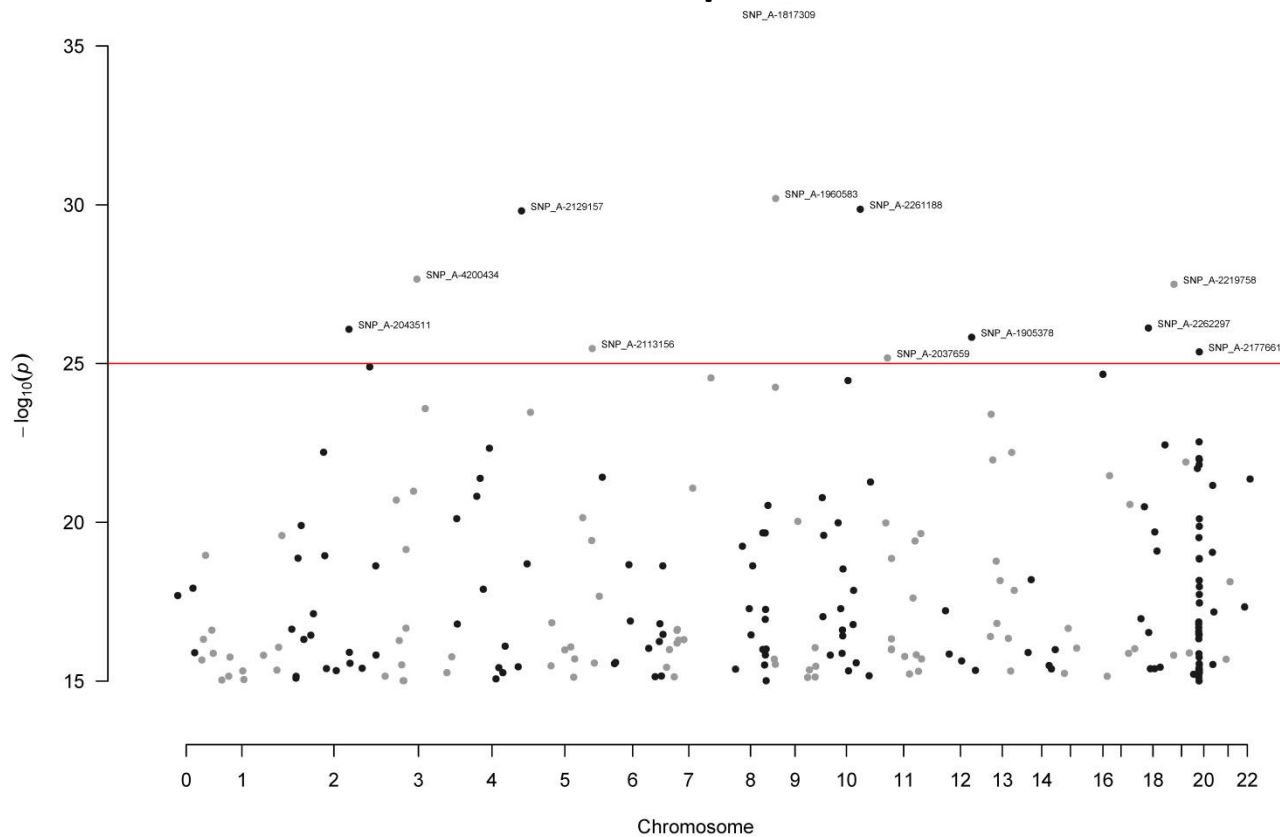

Supplement: Supplementary file 1 [file DataSheet1.ZIP › SI-figure/Figure S3.pdf]

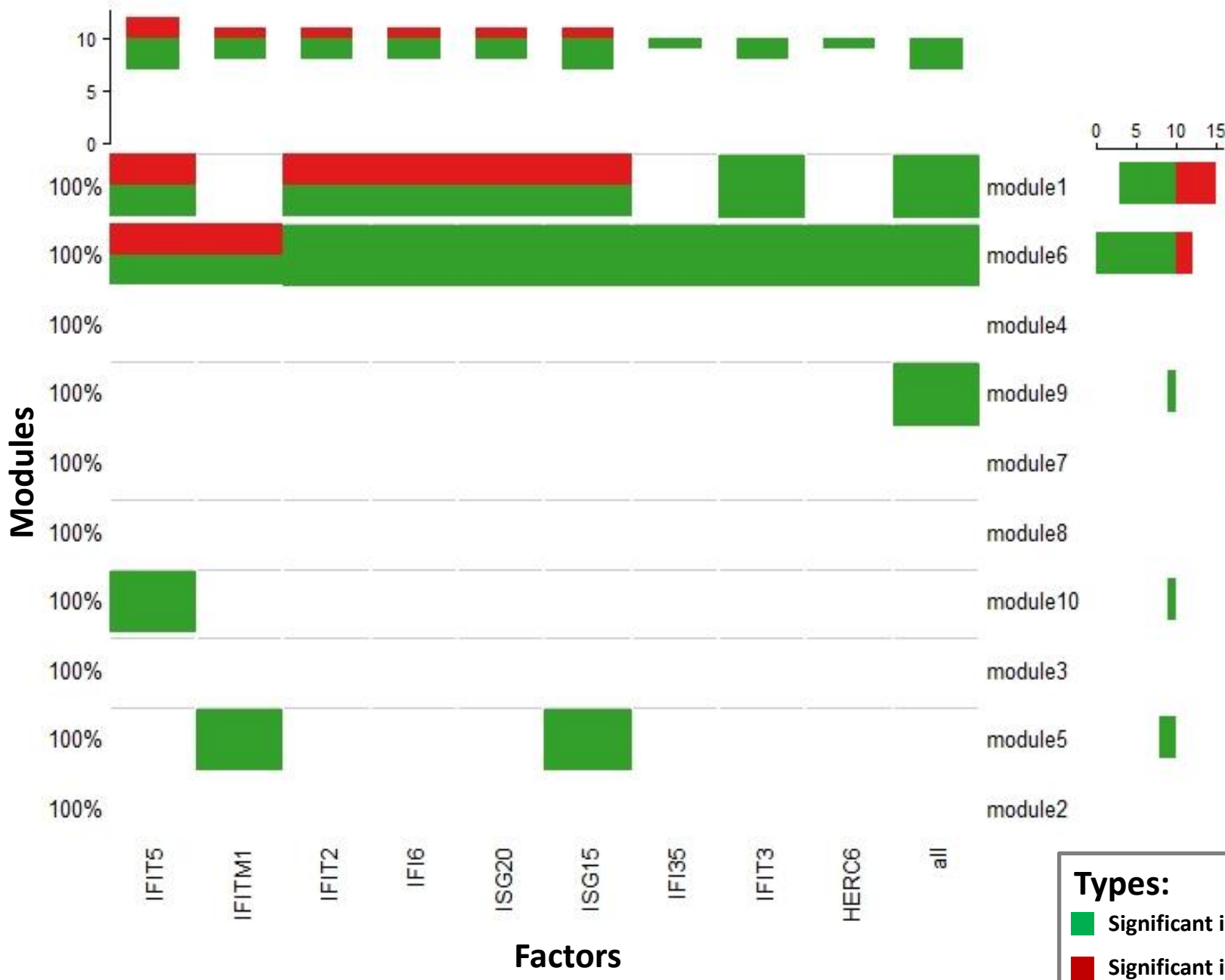

Supplement: Supplementary file 1 [file DataSheet1.ZIP › SI-figure/Figure S30.pdf]

(A)

Module-1

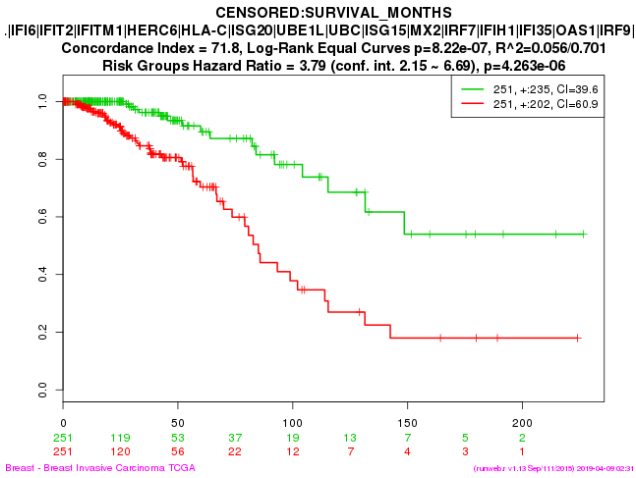

(B)

Module-2

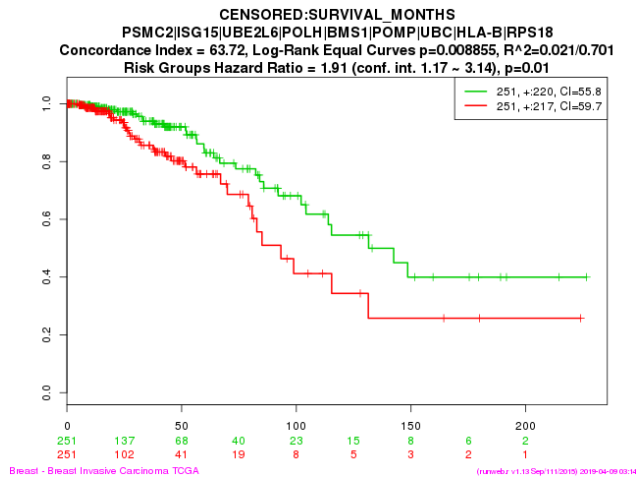

(C)

Module-3

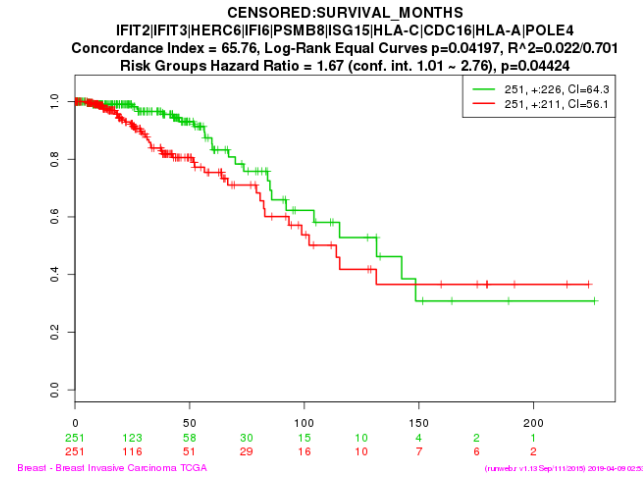

(D)

Module-4

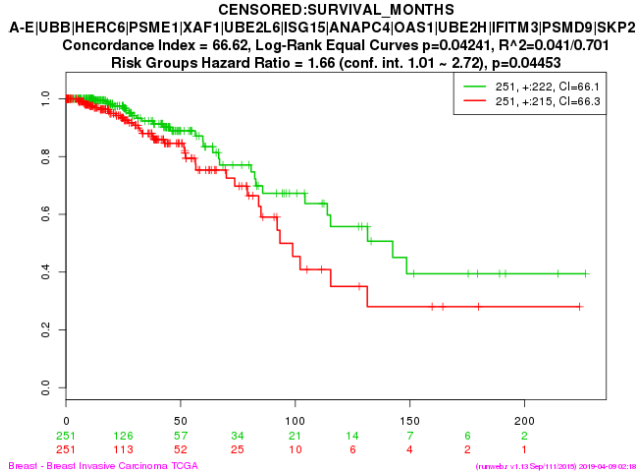

(E)

Module-5

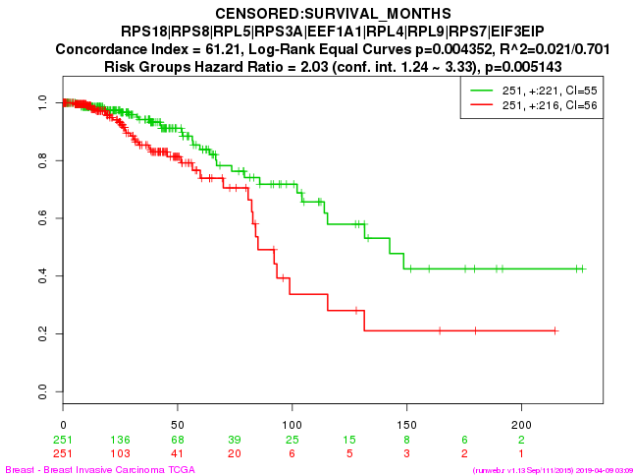

(F)

Module-6

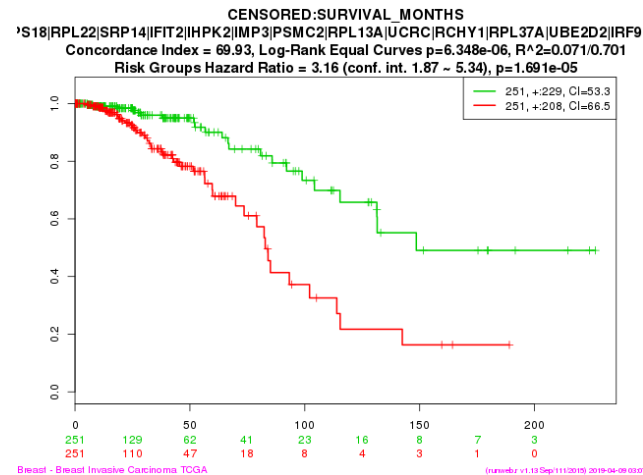

(G)

Module-7

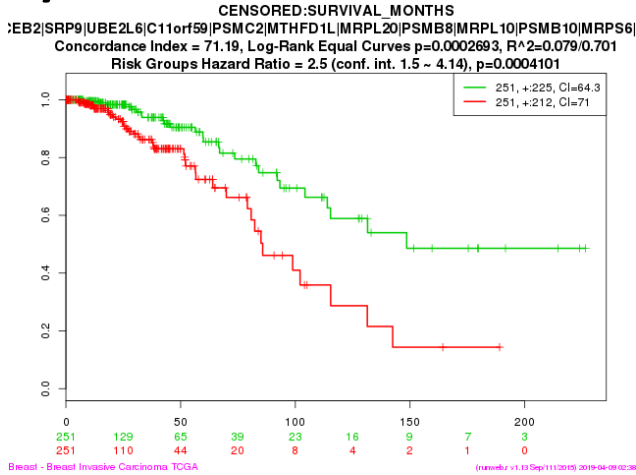

(H)

Module-8

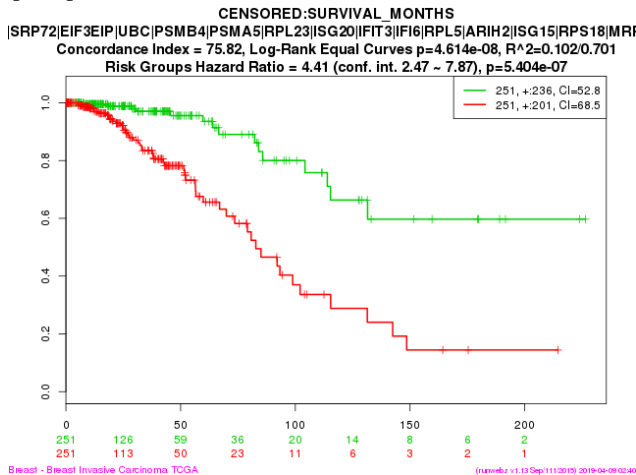

(I)

Module-9

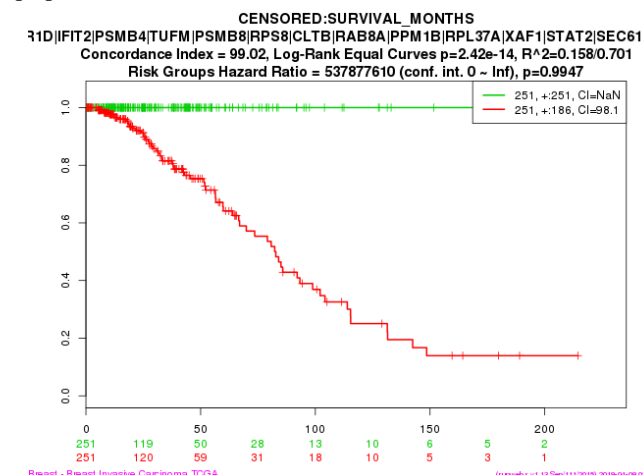

(J)

Module-10

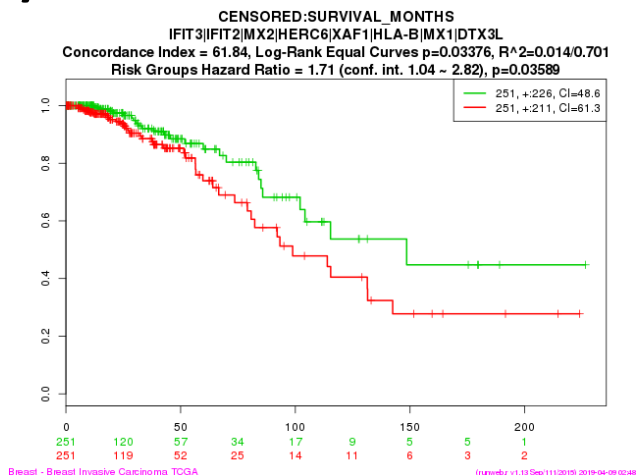

Supplement: Supplementary file 1 [file DataSheet1.ZIP › SI-figure/Figure S32.pdf]

(A)

Module-1

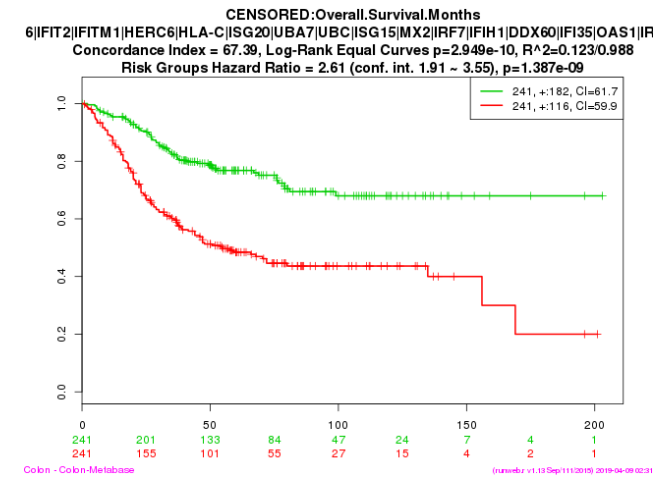

(B)

Module-2

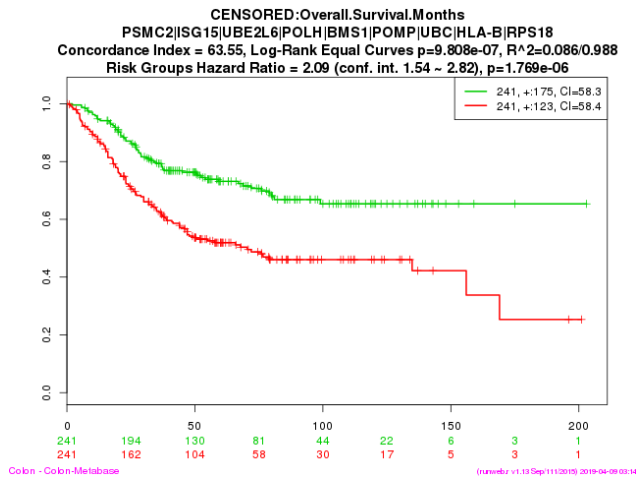

(C)

Module-3

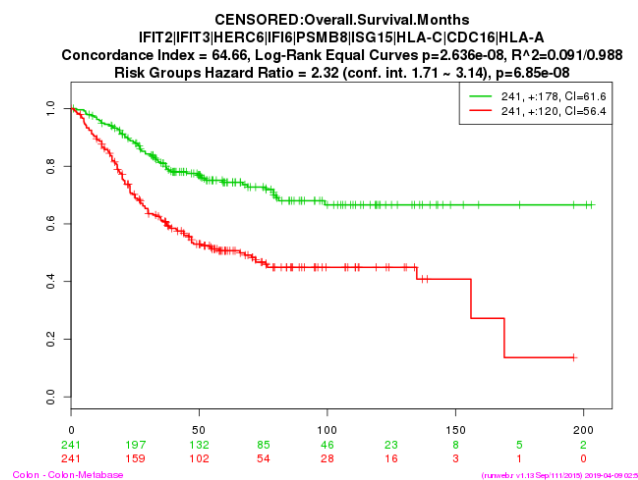

(D)

Module-4

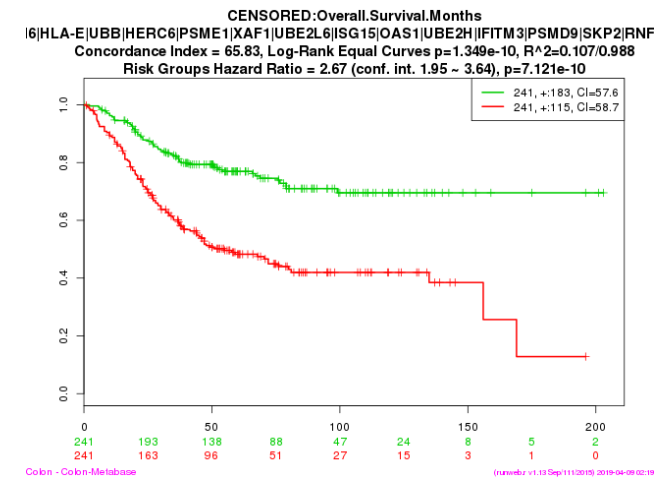

(E)

Module-5

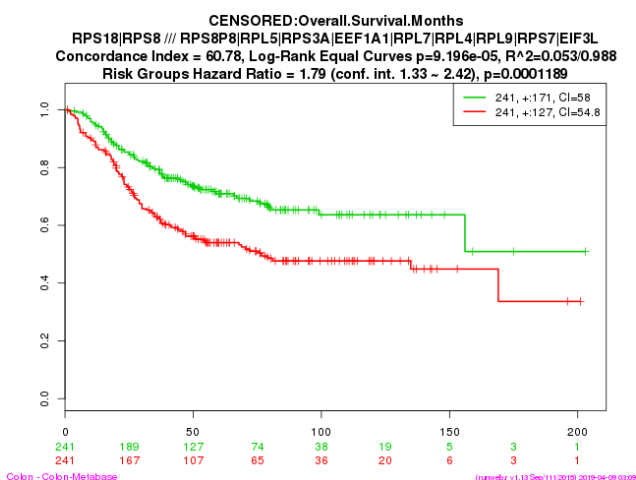

(F)

Module-6

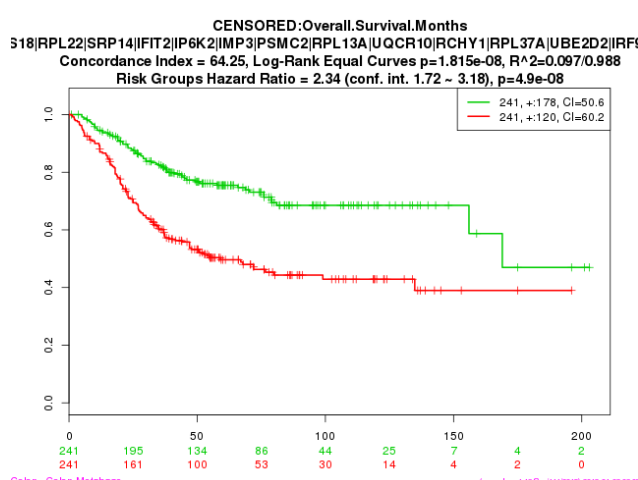

(G)

Module-7

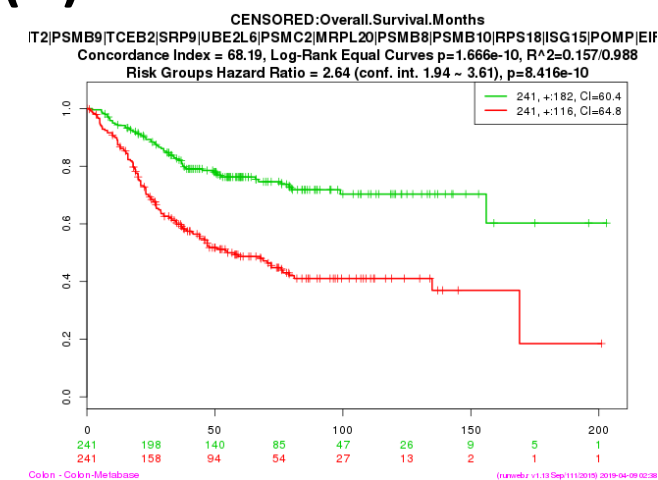

(H)

Module-8

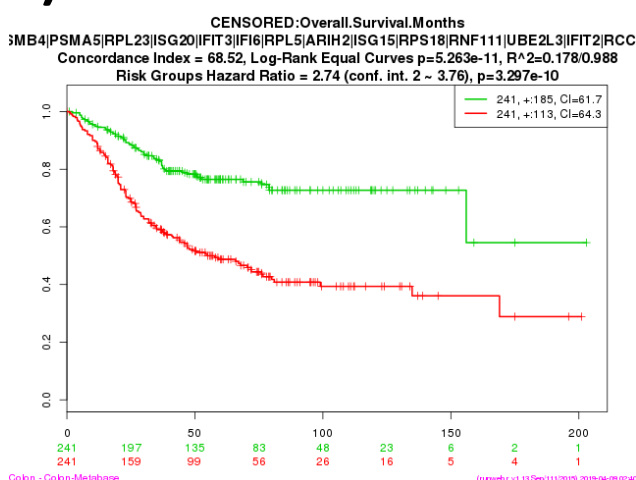

(I)

Module-9

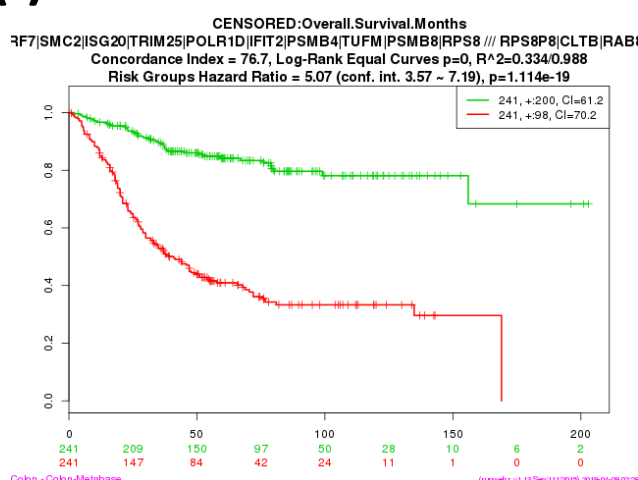

(J)

Module-10

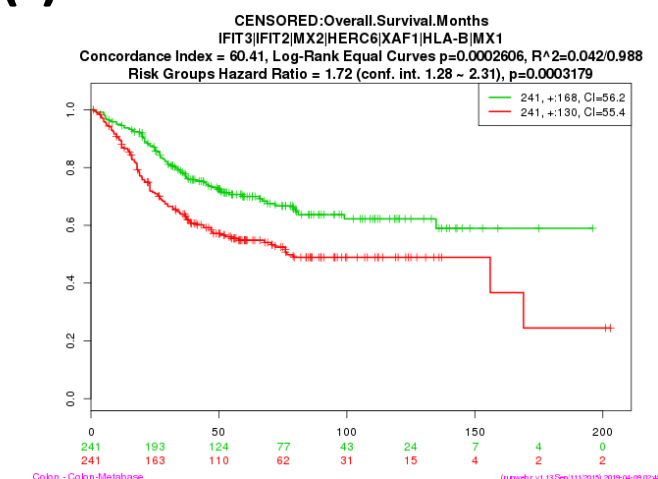

Supplement: Supplementary file 1 [file DataSheet1.ZIP › SI-figure/Figure S33.pdf]

(A)

### Module-1

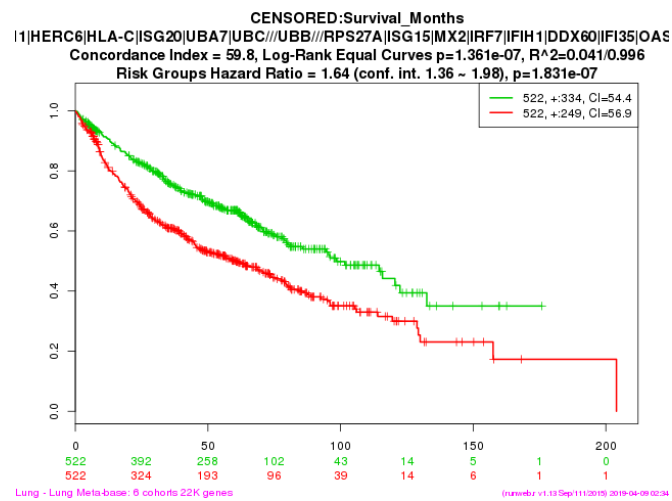

(B)

### Module-2

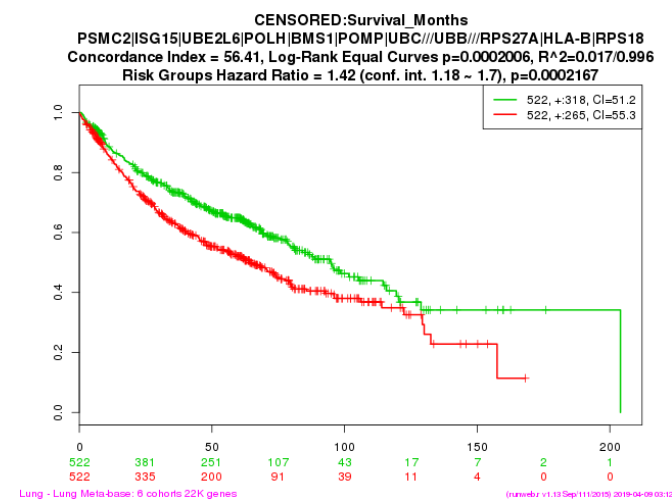

(C)

### Module-3

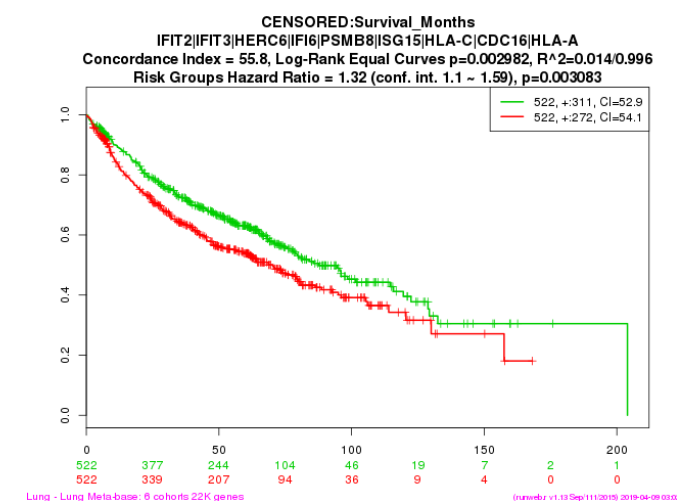

(D)

### Module-4

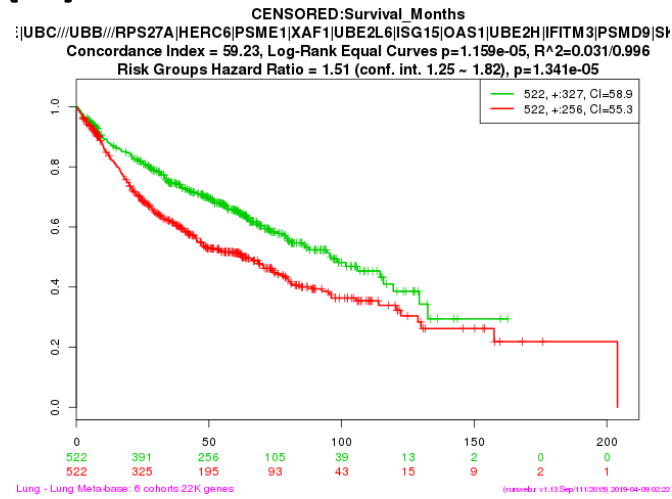

(E)

### Module-5

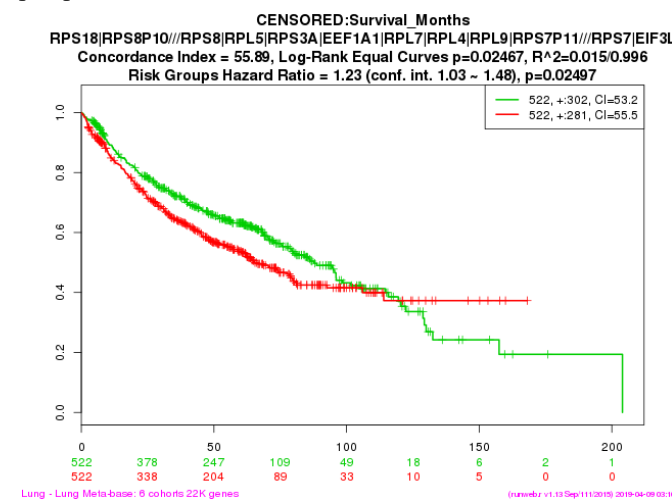

(F)

### Module-6

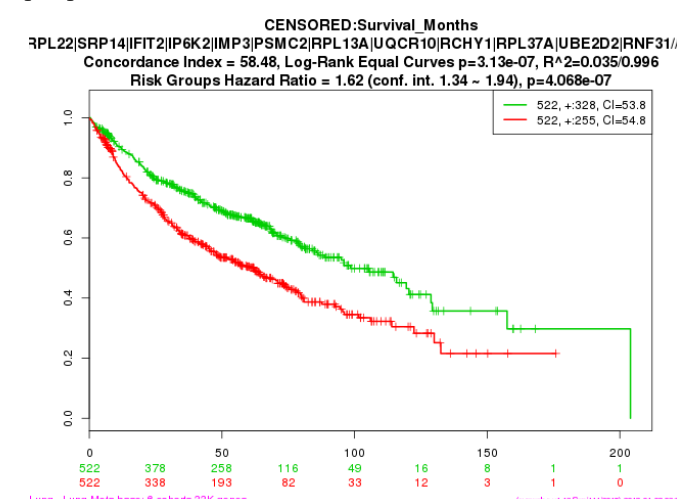

(G)

### Module-7

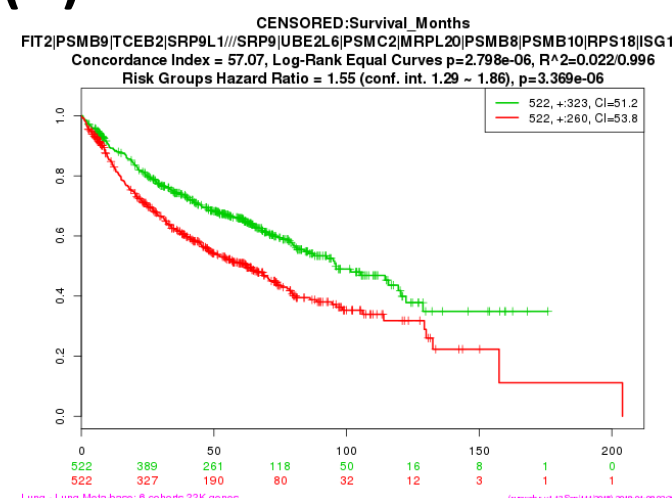

(H)

### Module-8

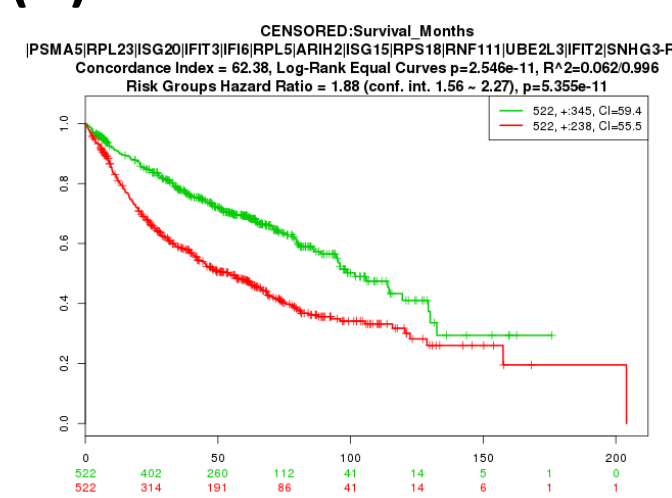

(I)

### Module-9

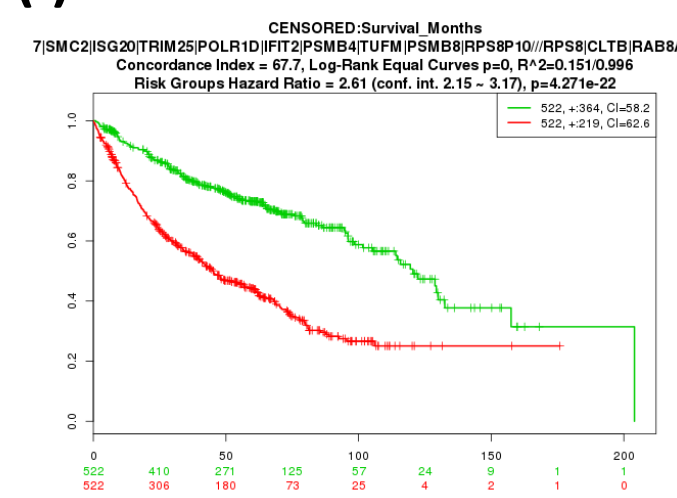

(J)

### Module-10

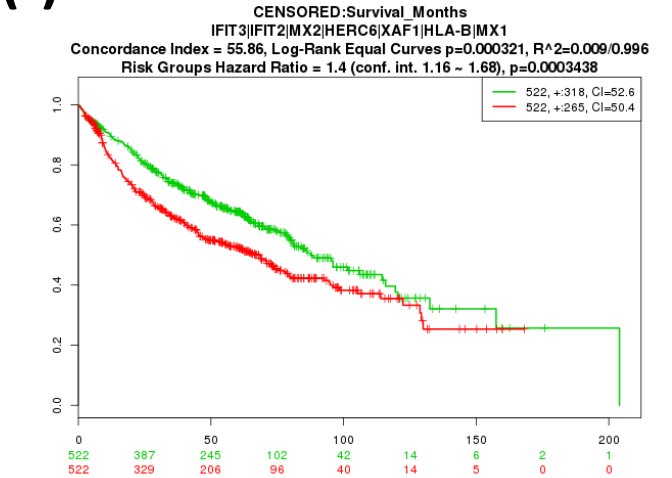

Supplement: Supplementary file 1 [file DataSheet1.ZIP › SI-figure/Figure S35.pdf]

(A)

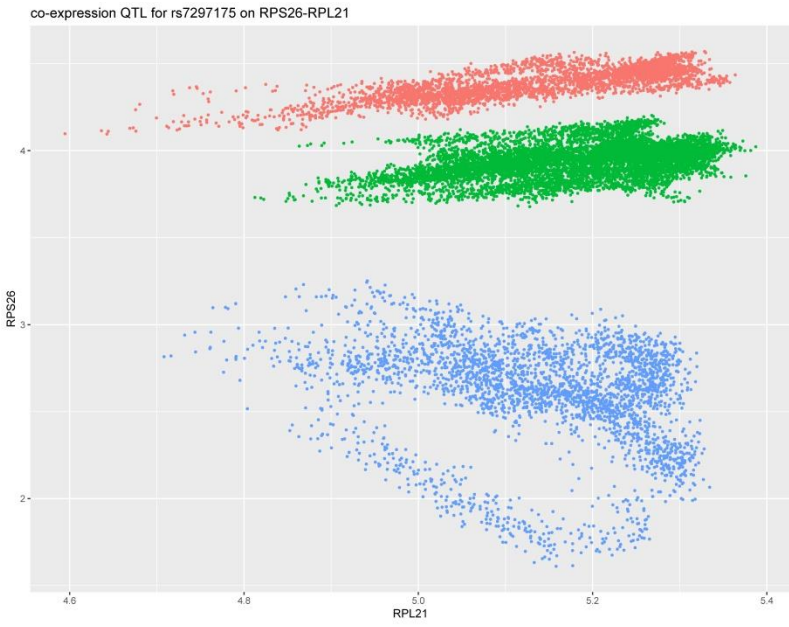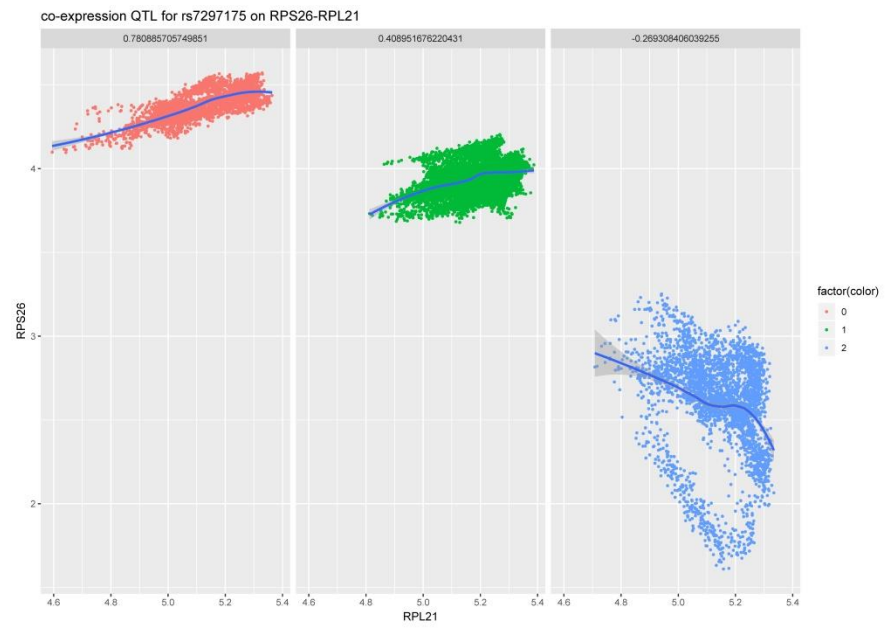

(B)

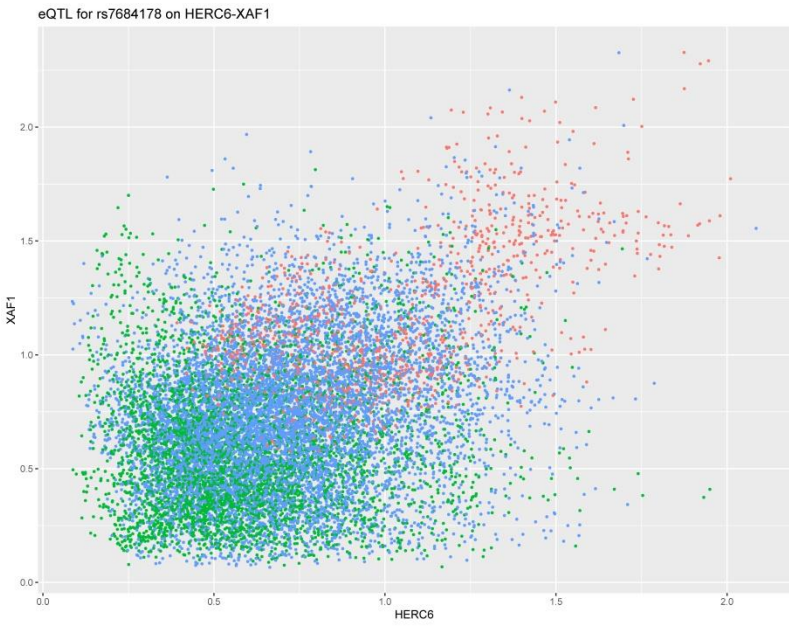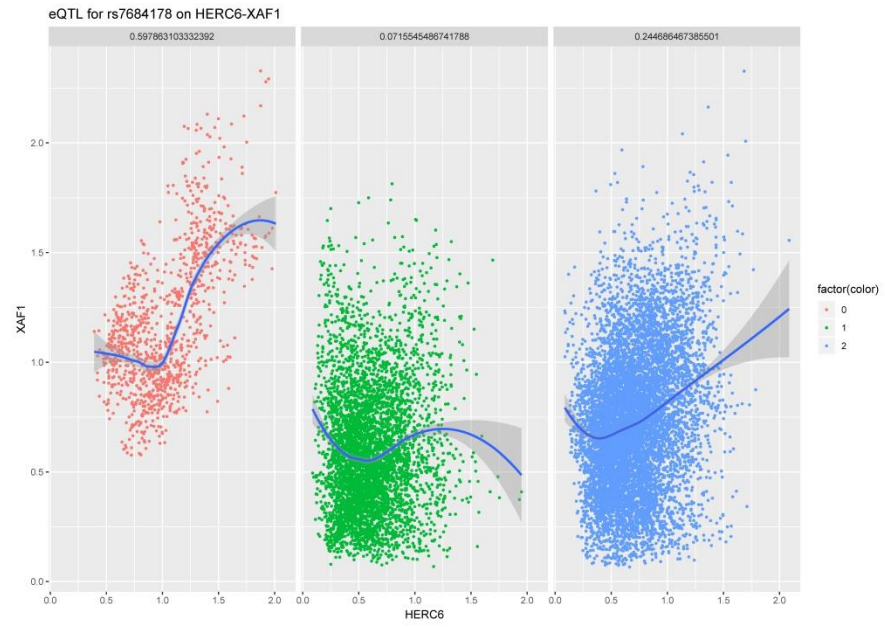

Supplement: Supplementary file 1 [file DataSheet1.ZIP › SI-figure/Figure S37.pdf]

nqtl-eqtl snps venn plot

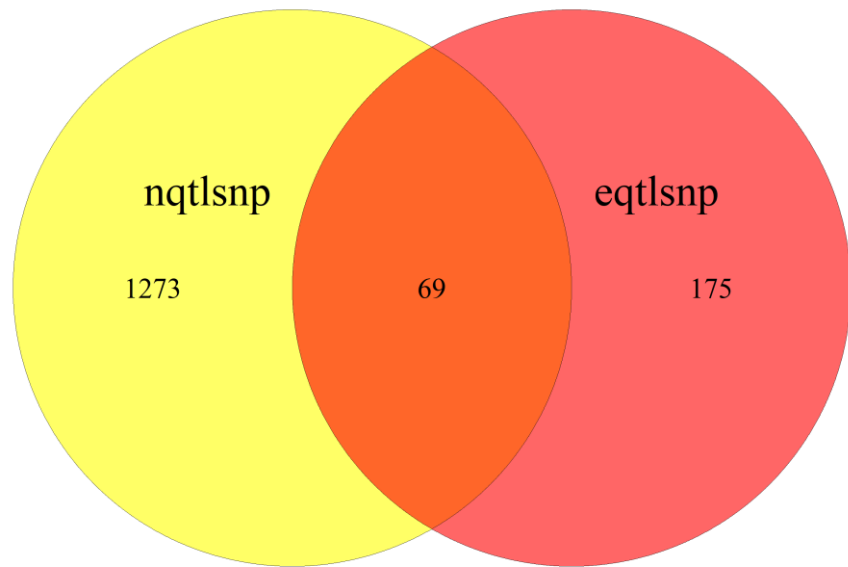

**(A)**

nqtl-eqtl genes venn plot

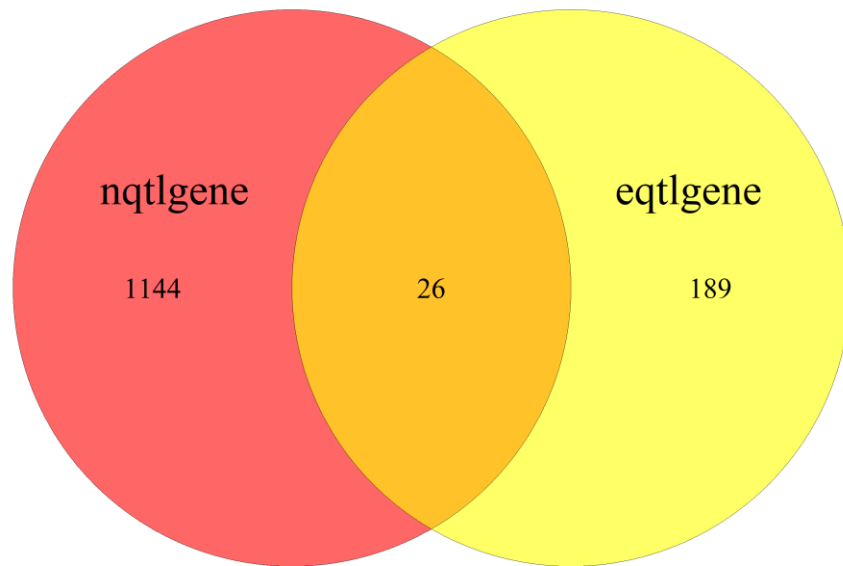

**(B)**

Supplement: Supplementary file 1 [file DataSheet1.ZIP › SI-figure/Figure S4.pdf]

Gene-pair & Snp association matrix

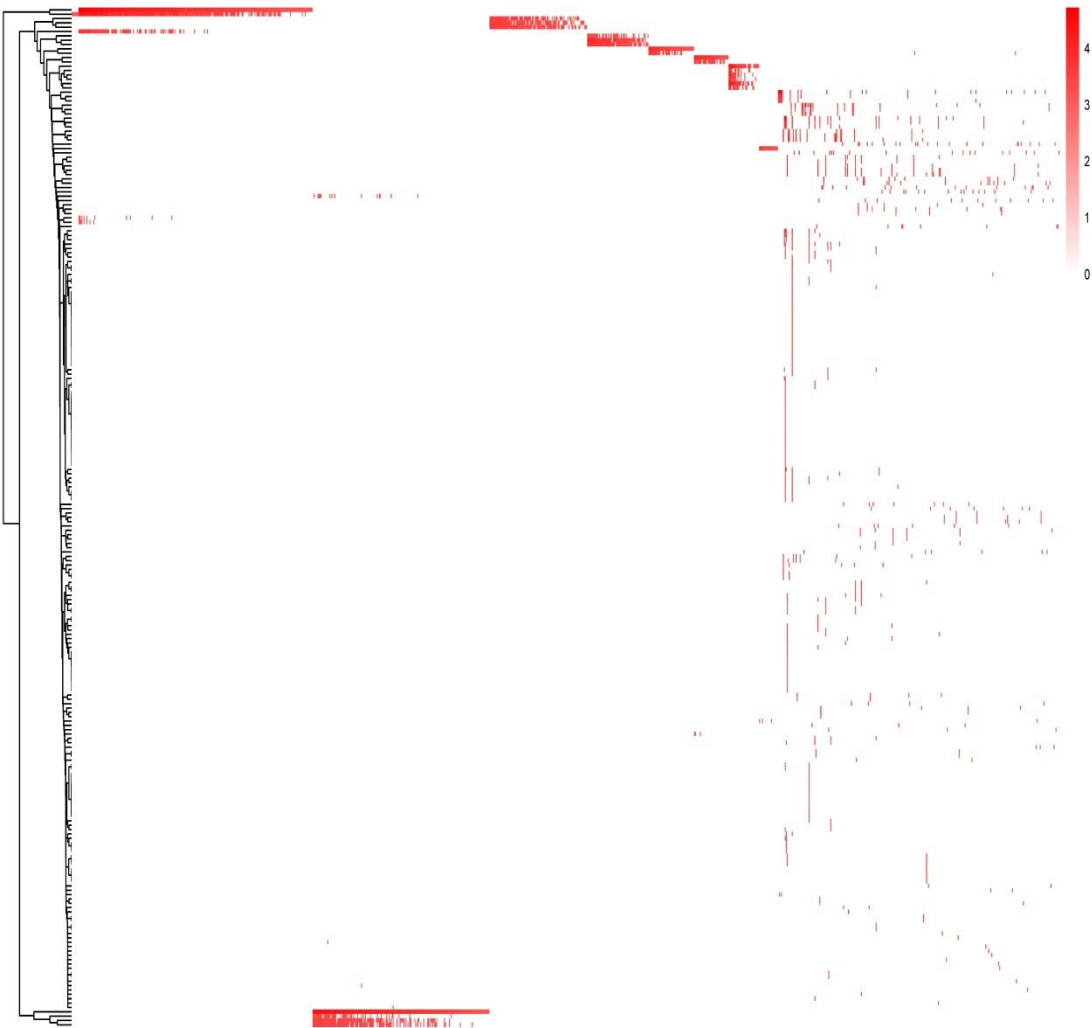

(A)

Gene-pair association matrix

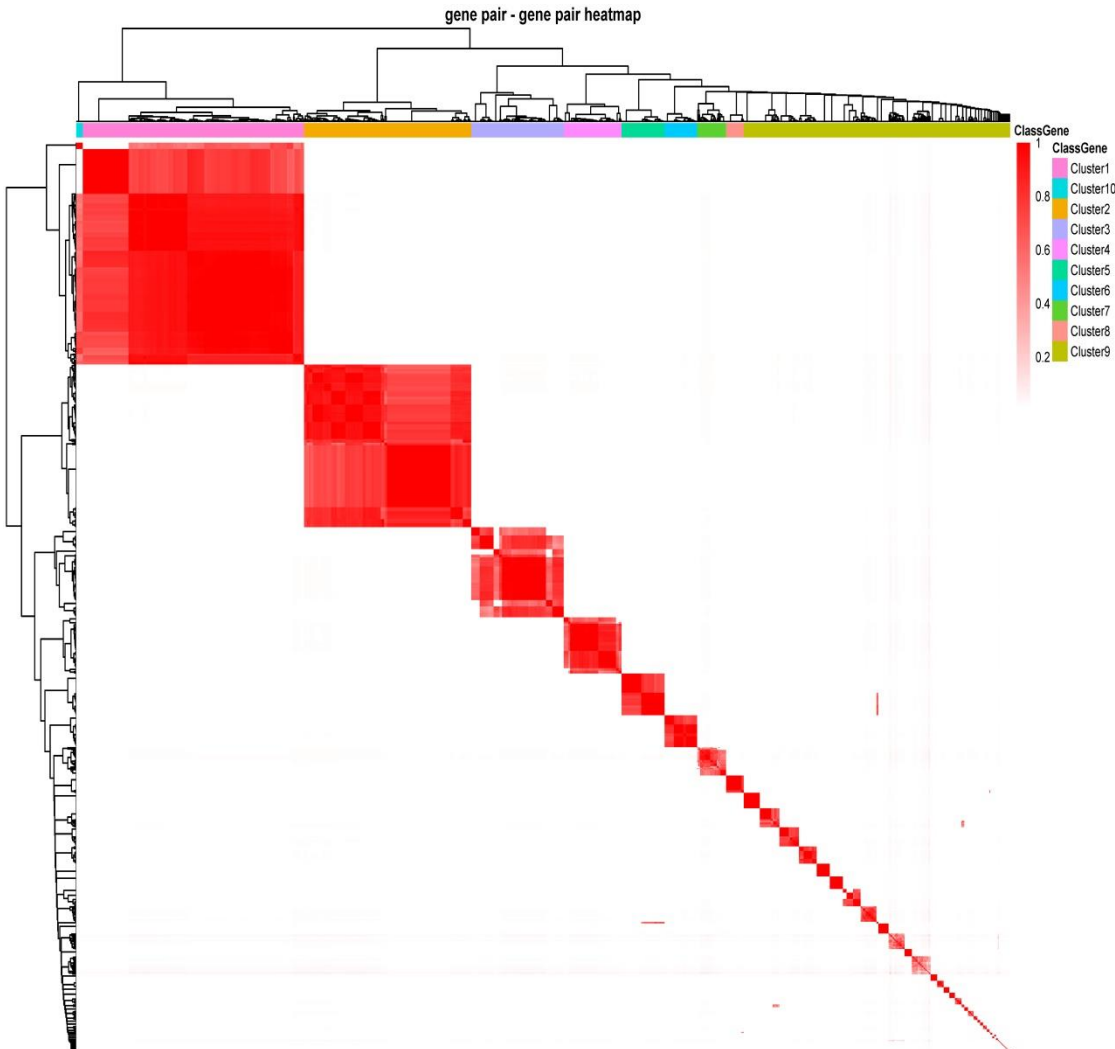

(B)

Supplement: Supplementary file 1 [file DataSheet1.ZIP › SI-figure/Figure S5.pdf]

(A)

Module-1

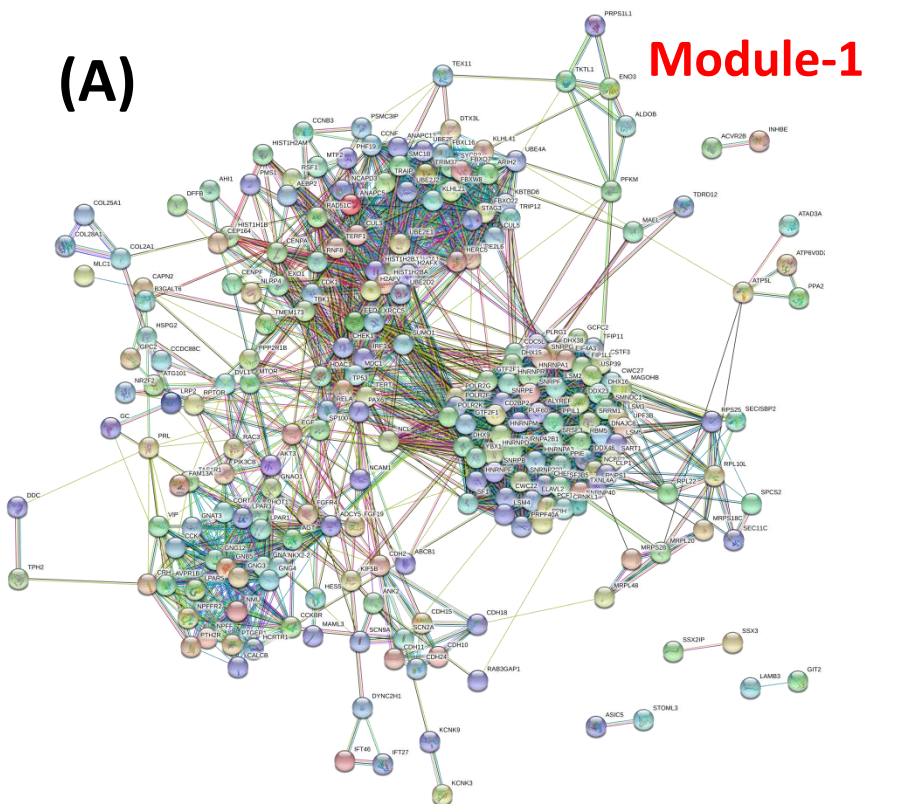

(B)

Module-2

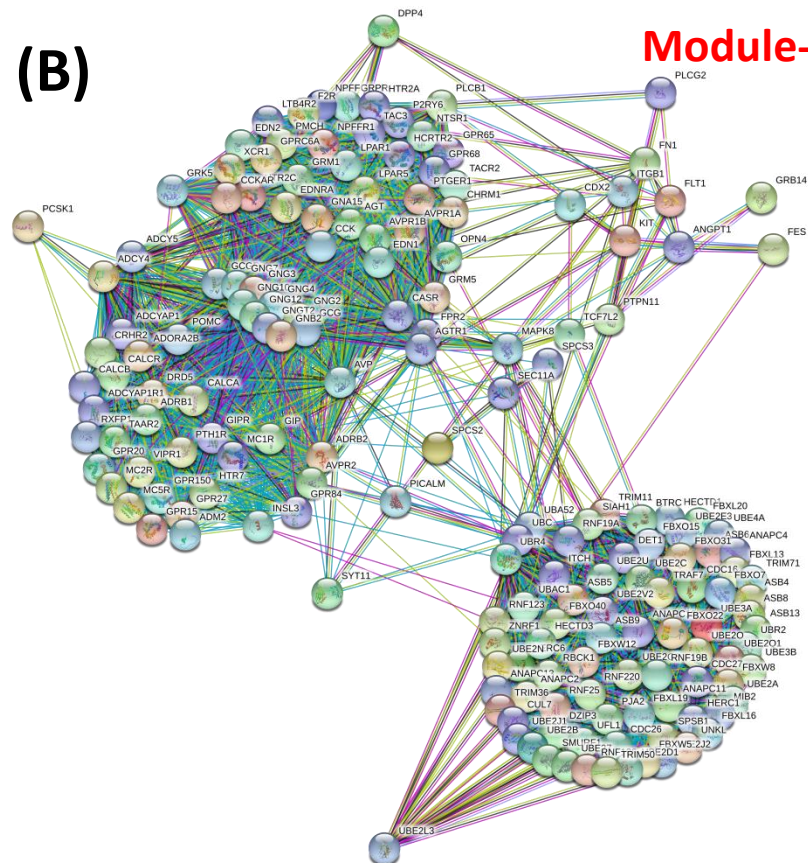

(C)

Module-3

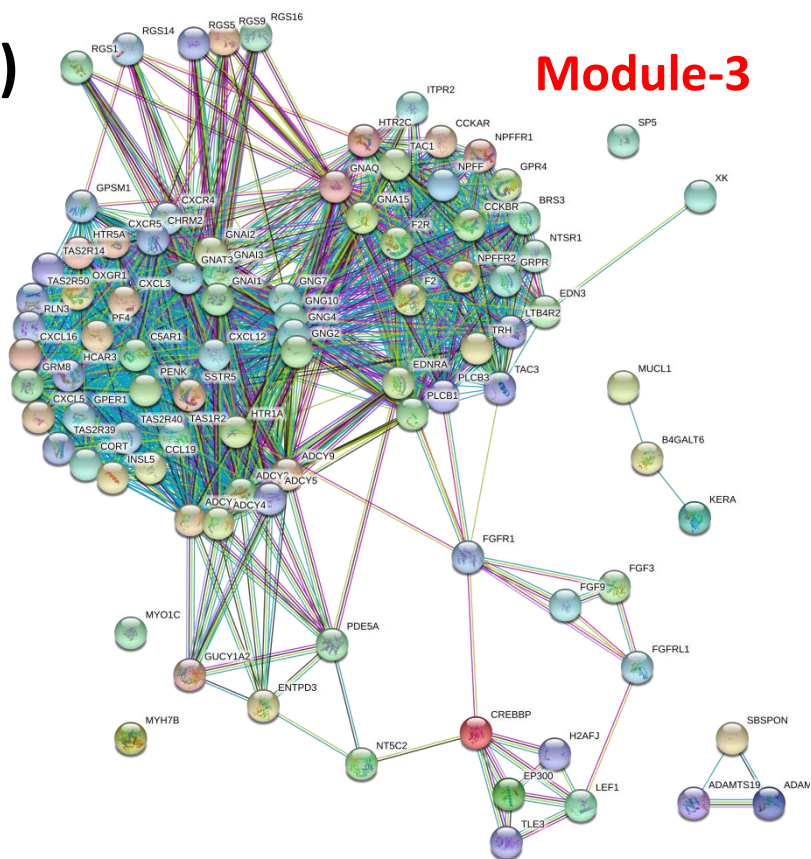

(D)

Module-10

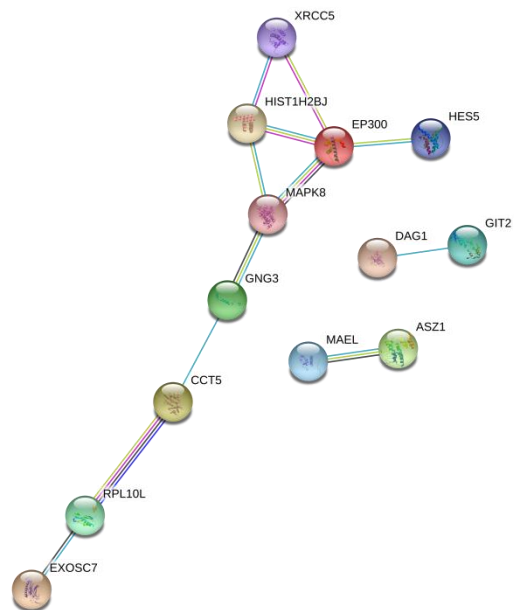

Supplement: Supplementary file 1 [file DataSheet1.ZIP › SI-figure/Figure S6.pdf]

**(A)****Module-1**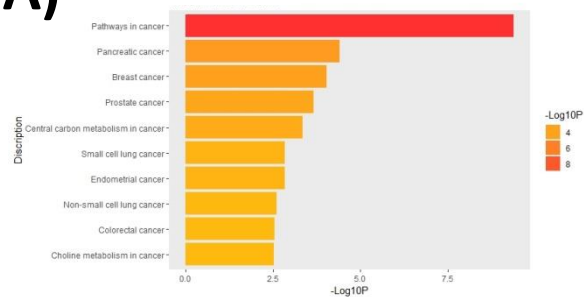**(B)****Module-2**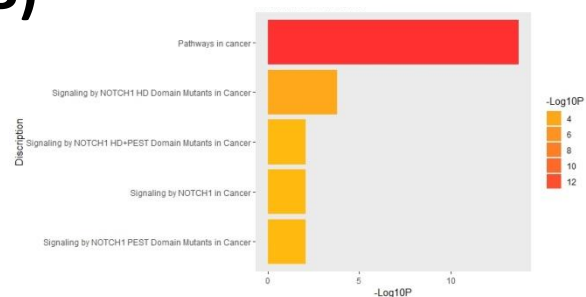**(C)****Module-3**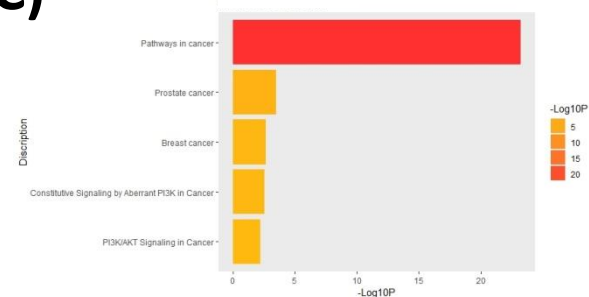**(D)****Module-4**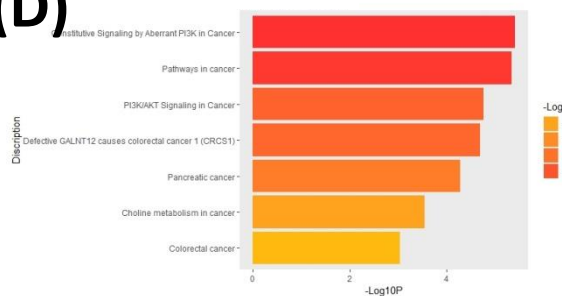**(E)****Module-5**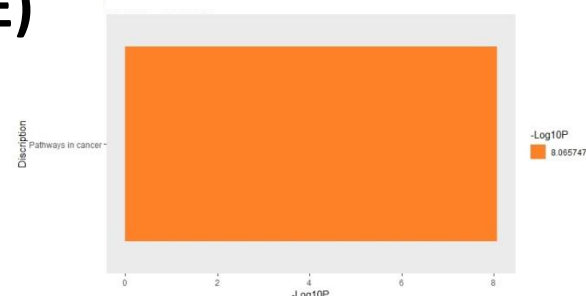**(F)****Module-7**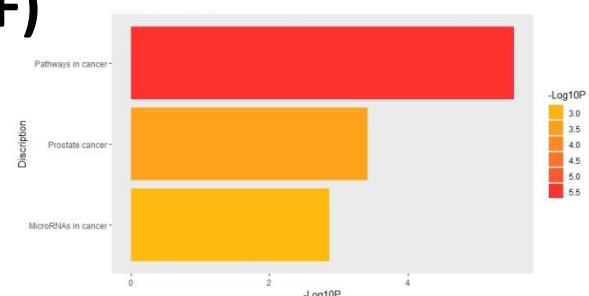**(G)****Module-8**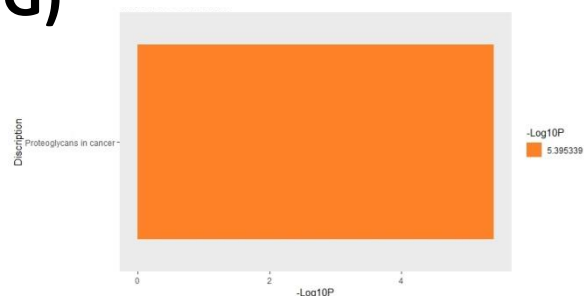**(H)****Module-9**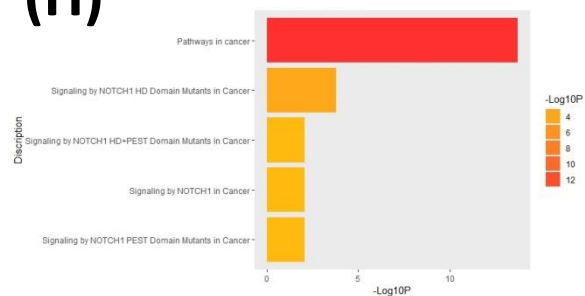**(I)****Module-10**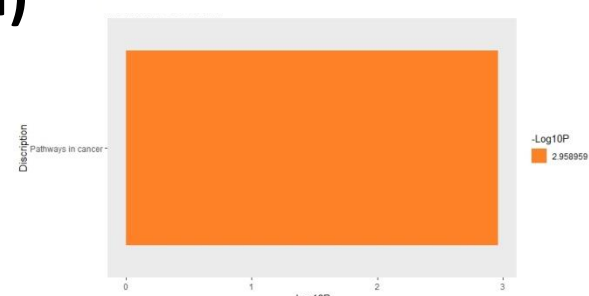

Supplement: Supplementary file 1 [file DataSheet1.ZIP › SI-figure/Figure S8.pdf]

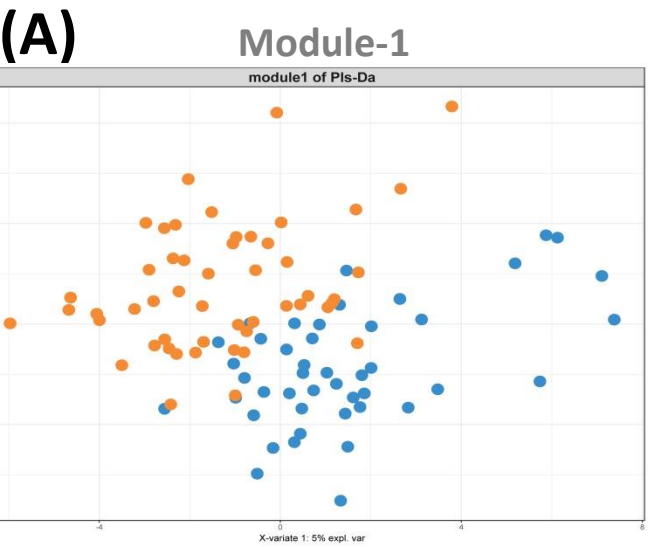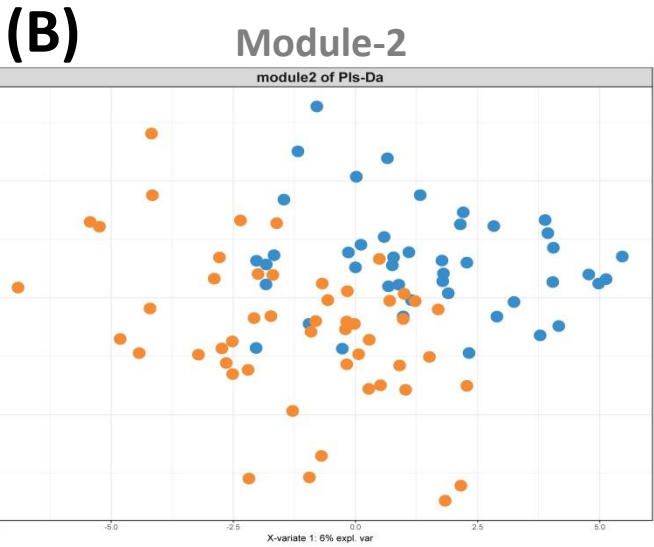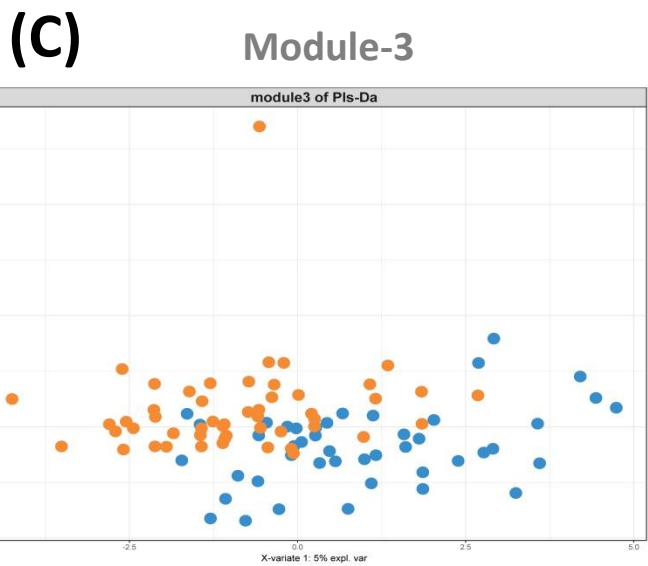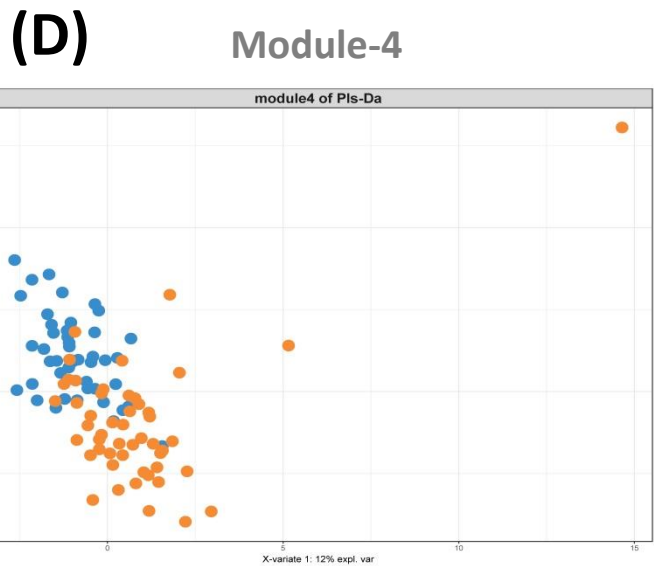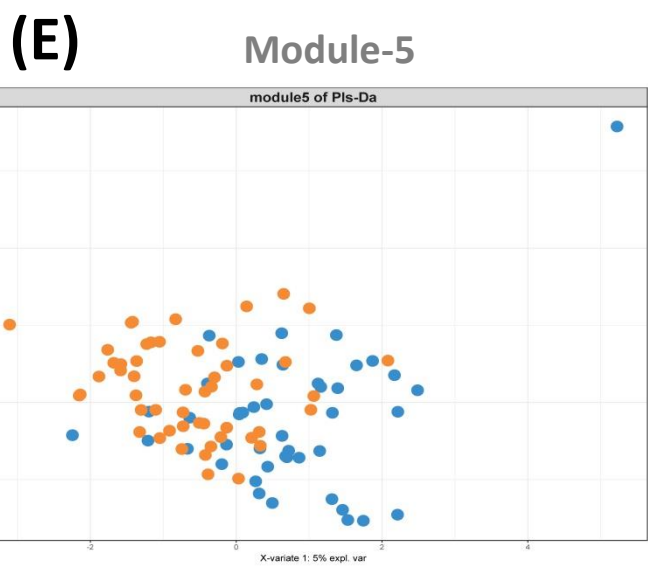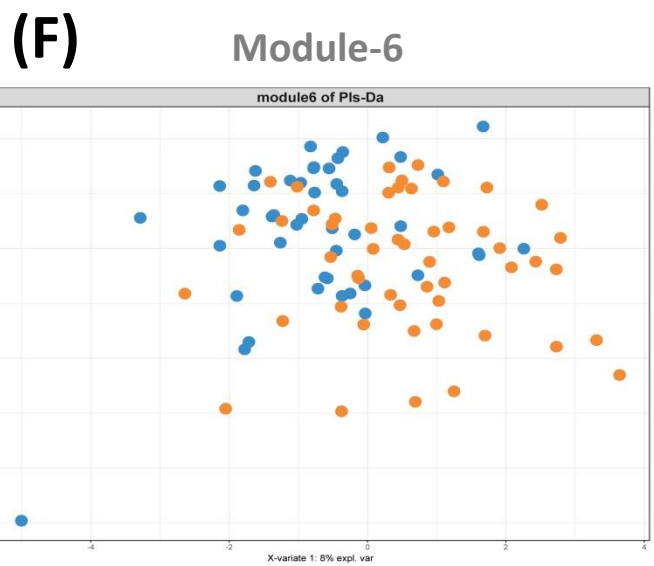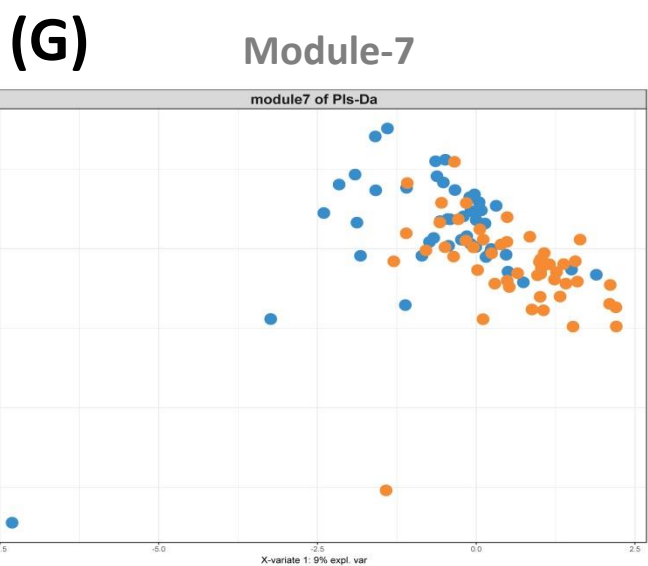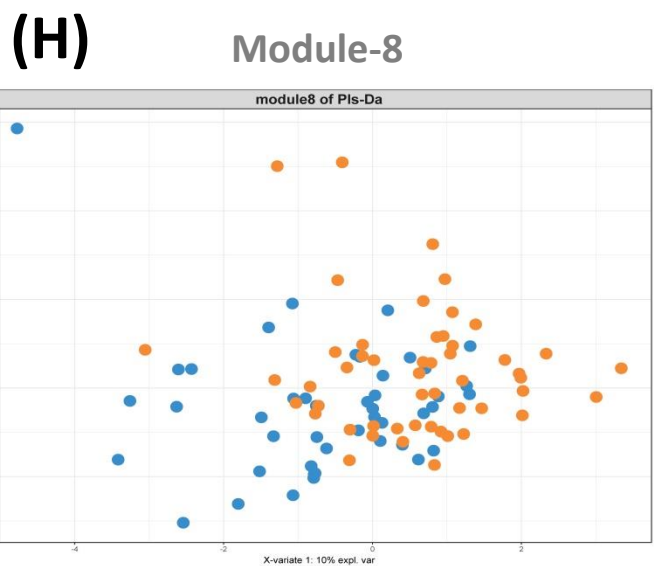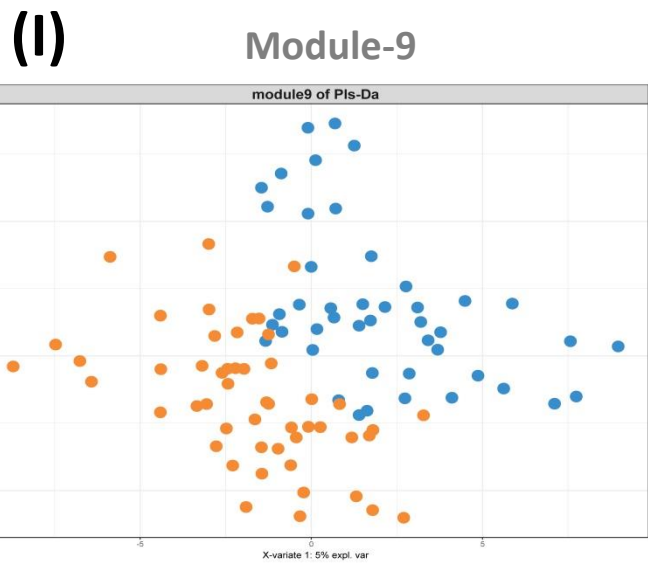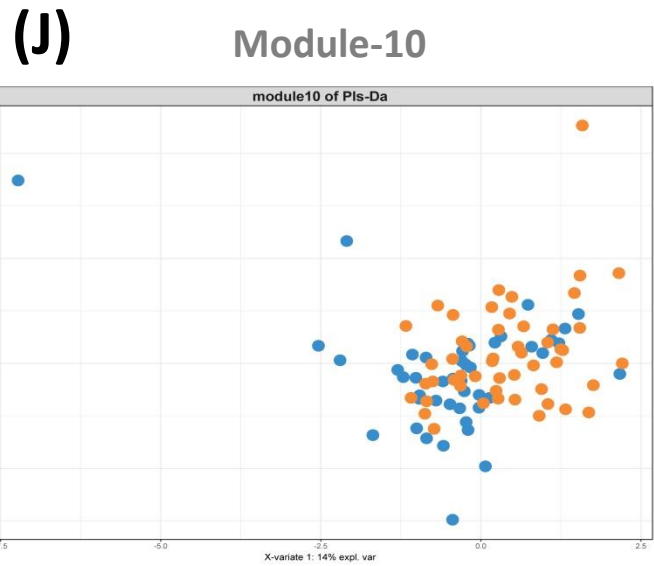

Supplement: Supplementary file 1 [file DataSheet1.ZIP › SI-figure/Figure S9.pdf]
